# Marine-derived phlorotannins: sustainable inhibitors of multiple virulence factors in *Pseudomonas aeruginosa*

**DOI:** 10.1186/s13568-025-01963-w

**Published:** 2025-10-31

**Authors:** Abirami Karthikeyan, Aqib Javaid, Nazia Tabassum, Tae-Hee Kim, Young-Mog Kim, Won-Kyo Jung, Fazlurrahman Khan

**Affiliations:** 1 Industry 4.0 Convergence Bionics Engineering, Pukyong National University, Busan, 48513 Republic of Korea; 2 Interdisciplinary Program of Marine and Fisheries Sciences and Convergent Technology, Pukyong National University, Busan, 48513 Republic of Korea; 3 Marine Integrated Biomedical Technology Center, The National Key Research Institutes in Universities, Pukyong National University, Busan, 48513 Republic of Korea; 4 Research Center for Marine Integrated Bionics Technology, Pukyong National University, Busan, 48513 Republic of Korea; 5 Department of Food Science and Technology, Pukyong National University, Busan, 48513 Republic of Korea; 6 Major of Biomedical Engineering, Division of Smart Healthcare, College of Information Technology and Convergence and New-Senior Healthcare Innovation Center (BK21 Plus), Pukyong National University, Busan, Republic of Korea; 7 Ocean and Fisheries Development International Cooperation Institute, Pukyong National University, Busan, 48513 Republic of Korea; 8 International Graduate Program of Fisheries Science, Pukyong National University, Busan, 48513 Republic of Korea

**Keywords:** *Pseudomonas aeruginosa*, Phlorotannins, Antivirulence properties, Molecular docking and dynamics simulation, Mutagenicity and ADMET profiling

## Abstract

*Pseudomonas aeruginosa* is a highly adaptable opportunistic pathogen in diverse environments, causing plant, animal, and human infections. Its remarkable ability to resist antibiotics and deploy multiple virulence strategies is attributed to its large genome, horizontal gene transfer, and complex regulatory networks. In this study, we comprehensively investigated 15 structurally distinct phlorotannins against 18 major virulence-associated proteins, such as quorum-sensing proteins, adhesion proteins, exotoxins, siderophore receptors, secretion system components, proteases, motility, and biofilm formation. Molecular docking and 50-ns molecular dynamics simulations revealed that compounds such as 2-phloroeckol, 7-phloroeckol, phlorofucofuroeckol A, and phlorofucofuroeckol B formed strong and stable interactions with critical targets, type IV pilus biogenesis factor PilY1, ferripyoverdine receptor, and phenazine-1-carboxylate-methyltransferase, with binding free energies as low as − 12.24 kcal/mol. These compounds exhibited a wide range of non-covalent interactions, including hydrogen bonding and π-π stacking, with essential active site residues in target proteins. Drug-likeness and environmental safety assessments utilizing the pkCSM and VEGA (Q)SAR models revealed high oral bioavailability, low toxicity, minimal cytochrome P450 interactions, and mostly non-mutagenic profiles. This study reveals phlorotannins as prospective eco-friendly alternatives for reducing *P. aeruginosa* infection by addressing a broad spectrum of virulence factors with ecologically benign and biodegradable natural compounds.

**Graphical abstract:**

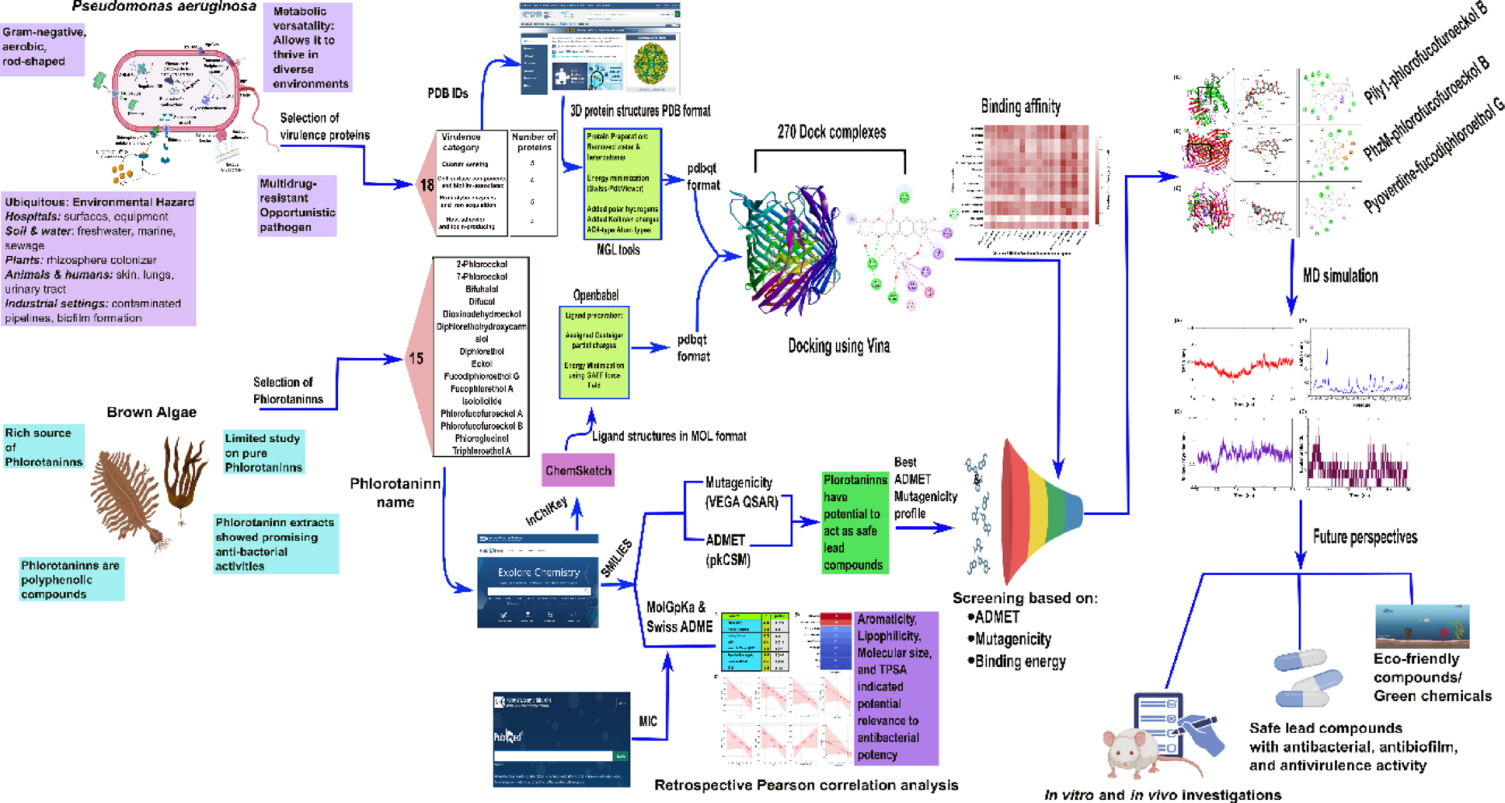

**Supplementary Information:**

The online version contains supplementary material available at 10.1186/s13568-025-01963-w.

## Introduction

The alarming rise of multidrug-resistant bacterial infections presents an urgent global health concern. Among these, *Pseudomonas aeruginosa* stands out as a particularly challenging gram-negative opportunistic pathogen due to its intrinsic resistance mechanisms and remarkable ability to acquire additional resistance traits (Behzadi et al. 2021). Frequently associated with nosocomial infections such as ventilator-associated pneumonia, urinary tract infections, and chronic wounds, *P. aeruginosa* poses a serious threat to immunocompromised individuals and patients with cystic fibrosis. Its resistance to multiple antibiotics, capacity for biofilm formation, and production of virulence factors underscore the urgent need for novel and effective antimicrobial agents (Streeter and Katouli 2016). Natural products, especially those derived from marine organisms, have emerged as promising sources of new bioactive compounds with therapeutic potential. Brown seaweeds, such as *Undaria pinnatifida*, *Laminaria japonica*, *Sargassum fulvellum*, and *Hizikia fusiforme*, have exhibited anti-inflammatory and antidiabetic effects in high-fat diet-induced mice. Specifically, *L. japonica* enhanced insulin sensitivity and decreased fasting glucose level (Oh et al. 2016). *Saccharina latissima* has an anti-obesity and hepatoprotective effect in a diet-induced mouse model. Furthermore, *Saccharina japonica* contains a low-molecular-weight sulfated polysaccharide that exhibits strong antioxidant activity (Jang et al. 2024). Marine brown algae are particularly rich in a unique class of polyphenols known as phlorotannins (Vo et al. 2019). These compounds exhibit diverse biological activities, including antioxidant, anticancer, anti-inflammatory, and antimicrobial effects. Notably, phlorotannins are exclusive to brown seaweeds and represent a structurally diverse group of secondary metabolites with potential pharmaceutical applications (R R et al., 2022). Phlorotannins were specifically selected in this study due to their previously reported activity against *P. aeruginosa*, a pathogen known for its complex virulence mechanism, including quorum sensing, biofilm formation, and multidrug efflux pumps (Echave et al. 2022; Fothergill et al. 2012). Additionally, the brown seaweed *Hizikia fusiforme* has shown specific inhibitory effects on *P. aeruginosa* communication and biofilm development, supporting the selection of this pathogen as a primary target over other pathogens (Tang et al. 2020). *P. aeruginosa* regulates the virulence and biofilm formation through a quorum-sensing network comprising Las, Rhl, and PQS circuits and uses signaling molecules to regulate gene expression in response to cell density (Vadakkan et al. 2024). This study of eighteen virulence-associated proteins was selected based on their experimentally confirmed role in pathogenicity. It encompasses quorum-sensing regulators, biofilm-associated proteins, proteases, adhesion proteins, and iron acquisition factors (Qin et al. 2022).

Previous studies on the antimicrobial activity of phlorotannins against *P. aeruginosa* have primarily relied on crude or semi-purified extracts from brown algae. While many of these extracts have demonstrated promising activity, the precise compounds responsible remain largely unidentified due to the heterogeneous nature of the mixtures (Shrestha et al. 2021). For instance, *Fucus vesiculosus* L. and *Pelvetia canaliculata* extract exhibit antibacterial activity against *P. aeruginosa* (Meshalkina et al. 2023). Numerous studies showed that phlorotannin extracts exhibit antimicrobial, anti-inflammatory, antidiabetic, anti-allergenic, and antioxidant effects as confirmed through protein-based assays and animal models. For example, *Fucus vesiculosus* extract significantly decreased the expression of inflammatory proteins in Raw 264.7 macrophages. Similarly, *Eisenia bicyclis* altered the expression of cytokines in THP-1 monocytes (Duan et al. 2025). However, lack of pure compound-specific data has hindered efforts to establish clear structure–activity relationships and mechanistic insights (Heffernan et al. 2015). To address this limitation, the present study focuses on a detailed* in silico* examination of phlorotannin compounds produced by brown algae. These compounds were selected based on the availability of their 3D structural data, their representation of the chemical diversity within the phlorotannin class, and previous studies on their biological importance.

A multi-tiered computational approach that integrates molecular docking, molecular dynamics (MD) simulations, *in silico* ADMET, and AMES toxicity predictions to evaluate the antibacterial potential of these phlorotannins against *P. aeruginosa* (Singothu and Bhandari 2024). Molecular docking and molecular dynamics (MD) simulations are crucial computational tools to investigate the interactions between small molecules and biological targets, aiding drug discovery and optimization (Naqvi et al. 2018). Docking enables virtual screening by modeling how compounds bind to target proteins, providing insights into binding affinity and potential inhibitory effects, thereby helping identify promising candidates before experimental validation (Ardala et al. 2008). MD simulations complement docking by analyzing the stability and flexibility of protein-ligand complexes over time, allowing researchers to assess structural changes, energy fluctuations, and interactions in a dynamic biological environment. Together, these approaches enhance precision in drug design, ensuring that selected molecules exhibit strong and sustained binding properties under physiological conditions (Hernández-Rodríguez et al. 2016). Key bacterial targets involved in virulence regulation and quorum sensing were selected for molecular docking to assess binding affinities and interaction profiles (Bhardwaj and Gupta 2022). The selection of virulence factors for molecular docking was based on their critical roles in quorum sensing, iron acquisition, and bacterial pathogenesis in *P. aeruginosa*, such as pyocyanin, pyoverdine, and elastase proteins. These proteins are essential for bacterial survival, host invasion, and antibiotic resistance (Soheili et al. 2015).

The present study employs a comprehensive computational approach to examine the relationship between phlorotannins and the virulence factors of *P. aeruginosa*. The following points are specifically addressed: (1) identification of key bacterial proteins associated with quorum sensing, motility, and biofilm formation for molecular docking analysis; (2) assessment of phlorotannin–protein interactions via molecular docking and molecular dynamics (MD) simulations to determine binding affinity and structural stability; (3) prediction of pharmacokinetics and mutagenicity through ADMET and AMES profiling to evaluate safety; and (4) future implications of phlorotannin-based anti-virulence strategies for addressing multidrug-resistant *P. aeruginosa*. The findings elucidate phlorotannin–protein interactions at the molecular level and support the development of marine-derived, sustainable antibacterial agents to combat drug-resistant *P. aeruginosa* infections.

## Materials and methods

### Selection of virulence factors/proteins of Pseudomonas aeruginosa and phlorotannins

A total of 18 virulence-associated proteins from *P. aeruginosa* were selected based on their experimentally validated roles reported in previous studies (**Supplementary Table ****S1**). These targets were identified through literature mining and the Uniprot database. The selected proteins were categorized into quorum-sensing regulators, structural and biofilm-associated proteins, proteolytic enzymes, iron acquisition proteins, host adhesion factors, and toxin-producing proteins. These virulence factors significantly influence the bacterial infection process (Xu et al. 2019). Fifteen phlorotannins were selected based on their promising antibacterial activity, which had been demonstrated in previous studies (**Table S2**  ). For example, phlorotannins from the brown alga *Ecklonia kurome* exhibited a broad-spectrum bactericidal effect against food-borne pathogens, methicillin-resistant *Staphylococcus aureus* (MRSA), and *Streptococcus pyogenes* (Nagayama et al. 2002). Phlorotannins from the brown algae *E. bicyclis* expressed antibacterial activity against streptomycin-resistant *Listeria monocytogenes* (Kim et al. 2018).

### Preparation of virulence factors/proteins and phlorotannins for Docking

The three-dimensional (3D) structure of all 18 virulence proteins was retrieved from the RCSB Protein Data Bank (https://www.rcsb.org) in the PDB format using their corresponding PDB IDs. Heteroatoms and water molecules were removed from the 3D protein structures (Manu et al. 2025; Manu, Nketia, Manu et al. 2025a, b). The resulting protein structures were subjected to energy minimization using SPDBV 4.10 to enhance structural stability (Johansson et al. 2012). Energy-minimized structures were then imported into AutoDock Tool 1.5.6, where polar hydrogens and Kollman charges were added, and atom types were assigned to the AD4-type. The final protein structures were saved in PDBQT format (Morris et al. 2009).

The InChIKey of selected phlorotannin compounds, including 2-phloroeckol, 7-phloroeckol, bifuhalol, difucol, dioxinodehydroeckol, diphloroethohydroxycarmalol, diphlorethol, eckol, fucodiphloroethol G, fucophlorethol A, isoliolide, phlorofucofuroeckol A, phlorofucofuroeckol B, phloroglucinol, and triphloroethol A, were obtained from PubChem. ChemSketch 2024.2.0 was used to generate the chemical structures of phlorotannins using InChIKey, and the structures were saved in Mol file format (Hunter 1997). Open Babel Software 3.1.1 was then employed to assign Gasteiger charges to each ligand, followed by energy minimization using the GAFF force field, and the resulting compounds were converted from Mol to PDB file format, and subsequently to PDBQT format to ensure compatibility with docking analysis (O’Boyle et al. 2011).

### Correlation of physicochemical properties of phlorotannins with the previously reported MIC values

To evaluate the relationship between antibacterial activity and the physicochemical properties of phlorotannins, Pearson correlation of various physicochemical properties of phlorotannins with previously reported minimum inhibitory concentration (MIC) values was evaluated. The physicochemical properties of all phlorotannin compounds used in this study were predicted using MolGpKa and Swiss ADME. The pKa values were predicted using MolGpKa, which utilizes a graph-convolutional neural network model (Pan et al. 2021). Additionally, LogP, topological polar surface area (TPSA), molecular weight (MW), hydrogen bond acceptors and donors, rotatable bonds, and aromaticity were predicted using Swiss ADME (Daina et al. 2017).

### Molecular docking of phlorotannins into the active site of virulence factors/proteins

Following the preparation of the protein and ligand, the grid box center coordinates were manually adjusted based on the active binding site of the virulence proteins obtained from existing literature. For two PDB structures, 5TCB and 6ESZ, active binding sites were not reported; therefore web-based tool CASTp was used to make the prediction (Tian et al. 2018). The pockets were ranked based on solvent-accessible (Richards) surface area and volume, with the largest and most solvent-exposed cavities prioritized for further docking analyses. The grid box dimensions were set to 40 × 40 × 40 Å, with a default spacing of 0.5 Å. Upon acquiring the PDBQT files for all proteins and ligands, molecular docking analysis was performed using AutoDock Vina 1.2.0, and the results were exported in text and PDBQT file formats. Docking was carried out with an exhaustiveness value of 32, the number of modes was set to 10, and the energy range was set to 4 (Eberhardt et al. 2021). The PDBQT files, containing the ligand docking poses, were converted into PDB file format using Open Babel Software 3.1.1 (O’Boyle et al. 2011). The docked complexes are generated using the coordinates of the lowest binding free energy (kcal/mol) ligand docked poses. The selected ligand coordinates were incorporated into the protein PDB files to create the final docked complexes. These docked complexes were visualized and analyzed using BIOVIA Discovery Studio 2021, offering 2D and 3D visualizations of molecular interactions between the protein and ligand (BIOVIA Discovery Studio | Dassault Systèmes). A heat map was constructed to visualize the variation in binding free energy across all the phlorotannin compounds using the seaborn and matplotlib packages based on a custom Python script. This graphical representation facilitated comparison and helped identify the poses with the lowest binding energy for further investigation.

### Mutagenicity evaluation of phlorotannins using VEGA QSAR

The mutagenic potential of the phlorotannins was analyzed using the VEGA QSAR tool to determine their safety profile. VEGA has four different models, such as CAESAR- v. 2.1.14, ISS- v. 1.0.3, SarPy-IRFMN- v. 1.0.8, and KNN-Read-Across- v.1.0.1. Additionally, a CONSENSUS model - v. 1.0.4 provides an overall evaluation depending on the outcome and reliability of individual models. ISS and SarPy are expert systems, and KSS and CAESAR are statistical systems. The CAESAR system includes an expert system to reduce false negative predictions. In contrast, the CONSENSUS model shows reliability in the form of a consensus score between 0 and 1, where 0 indicates poor reliability and 1 indicates high reliability. The four models show reliability in the form of low, moderate, and high. A reliability score below 0.7 indicates low reliability, a score from 0.7 to 0.9 indicates moderate reliability, and a score between 0.9 and 1 indicates high reliability (Benfenati et al. 2013; Fischer et al. 2023).

### Pharmacokinetic analysis of phlorotannins using PkCSM

The pharmacokinetic properties of the phlorotannins were evaluated using the pkCSM-pharmacokinetics tool, which includes absorption, distribution, metabolism, excretion, and toxicity (ADMET). The investigation prioritized phlorotannins with effective absorption, distribution, metabolism, and excretion in the body. Additionally, the toxicity evaluation facilitates the exclusion of phlorotannins with adverse effects, thereby ensuring the safety of the compounds for further studies. The SMILES corresponding to each phlorotannin were retrieved from PubChem and entered into the pkCSM platform. The evaluation includes water solubility, fraction unbound, cytochrome P450 interactions, renal clearance, and toxicity risks (Pires et al. 2015).

### Molecular dynamics (MD) simulation of the best docking complex

Molecular dynamics simulations were carried out using GROMACS software version 2024.1 to understand how the protein-ligand complexes will behave under physiological conditions (Abraham et al. 2015). The Charmm 36 force field was used for parameterization. MD simulation was performed for the *P. aeruginosa* virulence proteins with phlorotannin structures, with a time of 50 ns to understand the ligand-induced structural stability. Three protein-ligand complexes were selected for the MD simulation based on docking score, non-mutagenic AMES profile, and the pharmacokinetic properties (Manu et al. 2024; Nketia et al. 2025). Starting with protein PDB and ligand PDB files, we generated GRO, topology (TOP), and positional restraint (POSRE) files for type IV pilus biogenesis factor PilY1-phlorofucofuroeckol B, phenazine-1-carboxylate N-methyltransferase -biosynthetic protein PhzM-phlorofucofuroeckol B, and ferripyoverdine receptor-fucodiphloroethol G docking complexes. A cubic box was generated around each complex separately and solvated with Single Point Charge (SPCE) water molecules. If the system did not attain the neutral charge, the counter-ions (Na^+^ or Cl^−^) were added to neutralize the system. Once the system achieved neutral charges, energy minimization was followed using the steepest descent approach, with 50,000 steps performed. Equilibration steps were performed at two different phases (NVT: Constant Number of particles, Volume, and Temperature, followed by NPT: Constant Number of particles, Pressure, and Temperature) with a time period of 100 ps, and then the final production MD simulation was run with a time period of 50 ns for each protein-phlorotannin complex. To evaluate structural dynamics and ligand-induced effects on protein stability, root mean square deviation (RMSD), root mean square fluctuation (RMSF), radius of gyration (Rg), and H-bond interactions were analyzed.

## Results

### Selection of Pseudomonas aeruginosa virulence proteins and phlorotannin

**Fig. 1:**
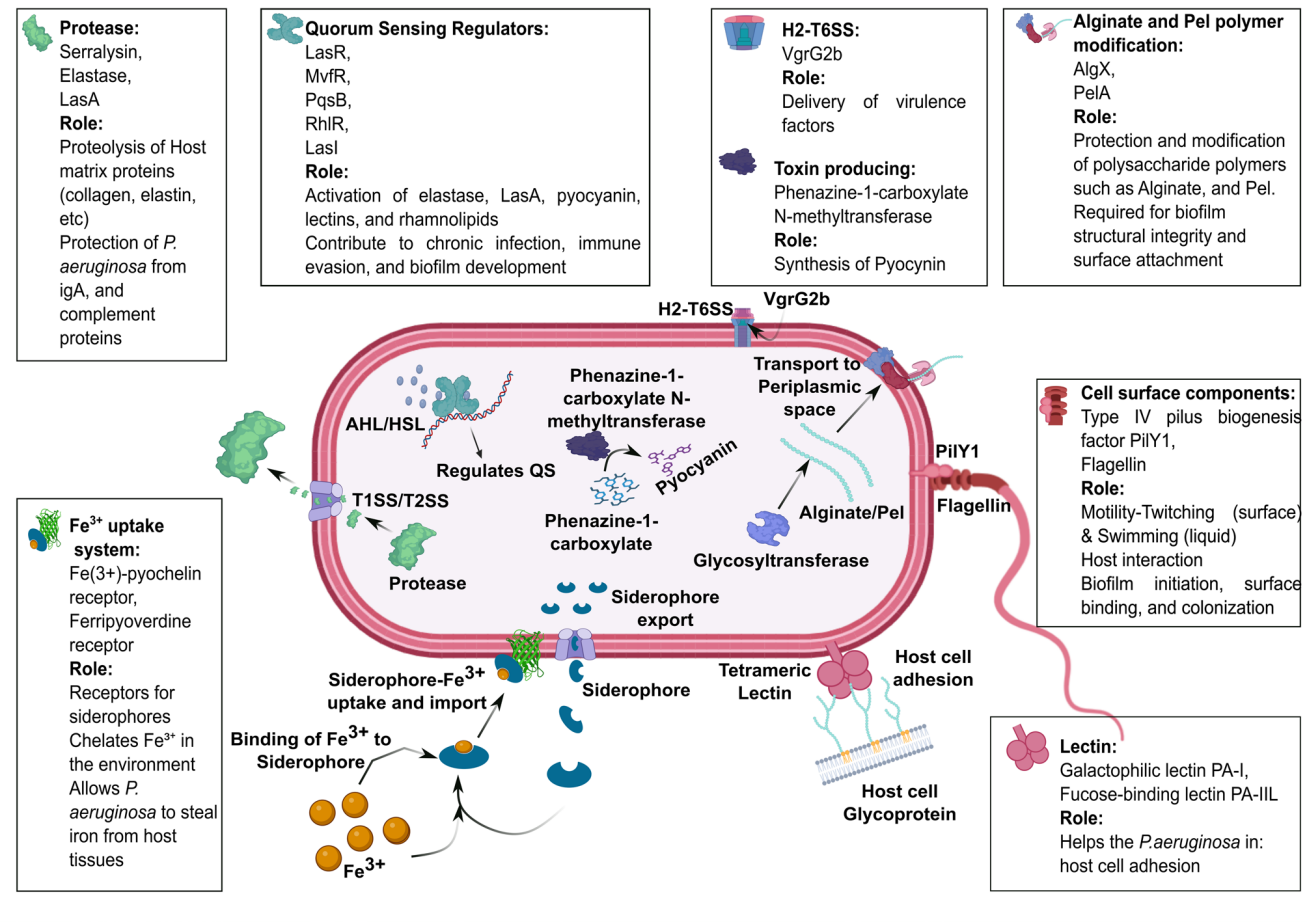
Selection of *P. aeruginosa* virulence proteins/factors and schematic illustration of their role in various kinds of virulence mechanisms and pathogenicity

In this study, eighteen virulence-associated proteins of *P. aeruginosa* were selected for molecular docking based on their previously reported functional roles in pathogenicity (**Table ****S1**). The selected proteins include quorum-sensing regulatory proteins, structural and biofilm-associated proteins, proteolytic enzymes, iron acquisition proteins, host interaction and toxin-producing proteins, which play a significant role in bacterial pathogenicity (Fig. 1). For instance, the quorum-sensing regulatory protein HTH-type quorum-sensing regulator RhlR controls the expression of multiple virulence factors. The biofilm-associated alginate biosynthesis protein AlgX protects alginate and enhances its adhesion to the host lung epithelium. The proteolytic enzyme elastase cleaves the host’s elastin and collagen. The host adhesion protein, a PA-I galactophilic lectin, facilitates the adherence of *P. aeruginosa* to the tissues of cystic fibrosis patients. All the proteins have been experimentally confirmed with evidence at the protein level in the UniProt database.

**Fig. 2:**
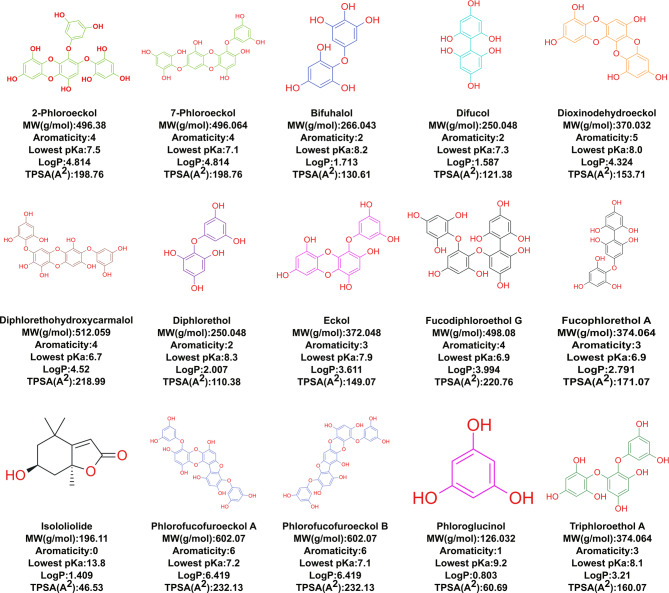
Chemical structures and physicochemical properties of selected phlorotannin compounds. Each compound is presented with its molecular weight (MW, g/mol), aromaticity score, lowest predicted pKa, calculated logP, and topological polar surface area (TPSA, Å2). These properties influence compound solubility, membrane permeability, and potential bioactivity. Structurally related compounds are shown in the same color, and all the electronegative centers, such as –OH groups, are highlighted in red

Similarly, fifteen phlorotannin compounds were selected as ligands based on their known antimicrobial activity. For example, phlorotannins from *E. bicyclis* and *Ecklonia cava* exhibited strong antibacterial activity against acne-related pathogens, MRSA, and *Salmonella* species, making them a promising candidate for natural antimicrobial treatment (Choi et al. 2010; Lee et al. 2014). Phlorotannin isolated from *E. bicyclis*, including eckol, 7-phloroeckol, dioxinodehydroeckol, phlorofucofuroeckol, exhibited antibacterial activity against antibiotic-resistant strains like *L. monocytogenes* and *S. aureus* through a multifaceted approach combining gene suppression, protein inhibition, and membrane disruption, highlighting their potential to mitigate antibiotic resistance (Eom et al. 2014; Kim et al. 2018). Furthermore, diphlorethohydroxycarmalol and phloroglucinol hindered the production of *P. aeruginosa* virulence factors such as pyocyanin, pyoverdine, and rhamnolipid, indicating their role in attenuating the pathogenicity (Khan et al. 2021; Kim et al. 2020). Detailed information on the selected phlorotannins, including their chemical and structural characteristics such as hydrophobicity, pKa, TPSA, and aromaticity, is presented in Fig. 2.

### Pearson correlation analysis of physicochemical properties of phlorotannins with the previously reported MIC values

**Fig. 3:**
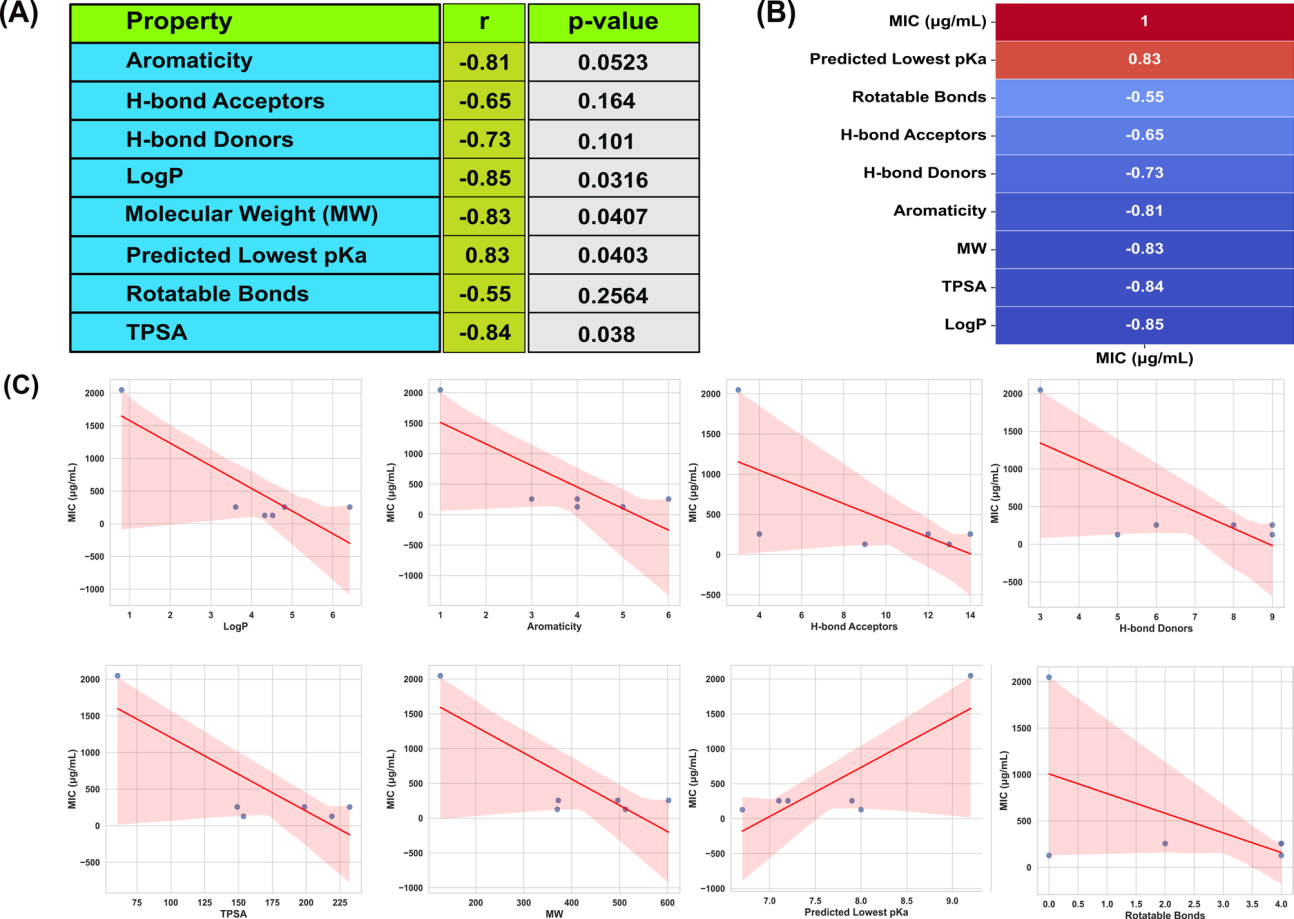
Correlation between physicochemical properties and antimicrobial activity (MIC). **A** Pearson correlation coefficients (r) and corresponding p-values between the highest previously reported MIC values and selected physicochemical properties of phlorotannin compounds (*n* = 6). Negative correlations indicate that higher values of the respective property are associated with lower MICs (i.e., stronger antibacterial activity), while positive correlations suggest the opposite. Statistically significant correlations (*p* < 0.05) are observed for LogP, molecular weight (MW), total polar surface area (TPSA), and predicted lowest pKa. **B** Correlation heatmap showing the Pearson correlation coefficients between the highest reported MIC values and physicochemical properties of phlorotannin compounds. Blue to red color gradient indicates the direction and strength of correlation, with red representing stronger negative correlations and blue representing stronger positive correlations. Higher absolute r-values indicate stronger linear relationships. Notably, LogP, TPSA, molecular weight, and aromaticity exhibit strong negative correlations with MIC, suggesting potential relevance to antibacterial potency. **C** Scatter plots with linear regression lines showing the relationship between MIC values and individual physicochemical properties of phlorotannin compounds. Each subplot illustrates one descriptor (e.g., LogP, TPSA, MW, etc.) against MIC, with a fitted trendline (in red) indicating the linear association with MIC

To assess the relationship between antibacterial potency and physicochemical features of the compounds, Pearson correlation coefficients (r) were calculated to evaluate the linear relationship between the highest reported minimum inhibitory concentration (MIC) values and various physicochemical properties of the tested compounds (**Table S2**). Using the highest MIC value per compound ensures a conservative estimate of activity, reflecting the least sensitive bacterial species tested. Although the sample size for known MIC values was small (*n* = 6), we found several physicochemical properties demonstrated strong correlations with MIC (Fig. 3). Aromaticity showed a strong negative correlation with MIC (*r* = -0.81, *p* = 0.052), approaching statistical significance. Significant negative correlations were observed for LogP (*r* = -0.85, *p* = 0.032), molecular weight (MW) (*r* = -0.83, *p* = 0.041), and total polar surface area (TPSA) (*r* = -0.84, *p* = 0.038), indicating that compounds with higher lipophilicity, larger molecular size, and greater polar surface area tended to have lower MIC values (i.e., higher antibacterial activity). Conversely, the predicted lowest pKa exhibited a strong positive correlation with MIC (*r* = 0.83, *p* = 0.040), suggesting that compounds with higher pKa values generally had higher MICs (lower activity). Other properties, such as hydrogen bond acceptors (*r* = -0.65, *p* = 0.16), hydrogen bond donors (*r* = -0.73, *p* = 0.10), and rotatable bonds (*r* = -0.55, *p* = 0.26), showed moderate but non-significant correlations. These findings suggest that aromaticity, lipophilicity, molecular size, and polar surface area might act as key physicochemical determinants of antibacterial activity in phlorotannins. However, given the limited sample size (non-availability of MIC data), these results need further validation with a larger sample size.

### Molecular docking of phlorotannins into the active site of virulence proteins/factors

Molecular docking analysis evaluated the binding affinities and molecular interactions between *P. aeruginosa* virulence proteins and the phlorotannins. The docking complexes were analyzed by evaluating their binding affinity, interaction type, and key amino acid residues. The overall binding energy for each protein-ligand complex was summarized in (**Table S3-S6**), which provides a comprehensive overview of binding free energy with values ranging from − 4.067 kcal/mol to -12,24 kcal/mol. Molecular docking studies showed that phlorotannins interact with *P. aeruginosa* virulence proteins by many non-covalent interactions, including conventional hydrogen bonds, van der Waals forces, π-π stacking, π-alkyl, π-anion, π-cation, π-sigma, π-π T-shaped, and salt bridge interactions, indicating significant affinity and rich interaction profiles. Here, we selected eighteen virulence-associated proteins that were categorized based on their role in bacterial pathogenicity. These categories include quorum-sensing regulating proteins, structural and biofilm-associated proteins, proteolytic enzymes, iron acquisition proteins, host adhesion, and toxin-producing virulence proteins.

### Molecular interactions of phlorotannins with quorum-sensing regulatory proteins

To evaluate the anti-virulence potential of phlorotannins against the QS-regulating proteins (LasR, MvfR, PqsB, RhlR, and LasI) (Manu et al. 2025; Manu, Nketia, Manu et al. 2025a, b), all 15 phlorotannin compounds were docked into the active site of these QS-regulating proteins, and interactions were summarized in **Table S3**.

Phlorotannin compounds exhibited binding energies with LasR ranging from − 6.296 to -9.865 kcal/mol, stabilized by diverse non-covalent interactions including conventional hydrogen bonds, van der Waals, π-anion, π-cation, π-sigma, π-π T-shaped, π-alkyl, π-π stacked, π-sulfur, amide π-stacked, alkyl, and π-donor hydrogen bonds, carbon-hydrogen bonds. Among the fifteen phlorotannins, difucol, diphlorethol, and fucophloroethol A exhibited low binding free energies, ranging from − 9.4 to -9.865 kcal/mol. Other compounds 2-phloroeckol, 7-phloroeckol, bifuhalol, dioxinodehydroeckol, diphloroethohydroxycarmalol, eckol, fucodiphloroethol G, isoliolide, phlorofucofuroeckol A, phlorofucofuroeckol B, and triphloroethol A showed moderate binding free energies (-7.072 to − 8.951 kcal/mol), and in contrast, phloroglucinol exhibited the weakest interaction profile with a binding free energy of -6.296 kcal/mol. Notably, five phlorotannins, difucol, dioxinodehydroeckol, phlorofucofuroeckol A, phlorofucofuroeckol B, and triphloroethol A, were involved in making π-π contacts with Tyr56, Phe101, Phe87, Phe87, and Phe101 residues of LasR, respectively. Difucol formed four conventional hydrogen bonds with Tyr93, Leu110, Thr115, Thr75, one π-π stacked and π-π T-shaped interaction with Tyr56, one π-anion interaction with Asp73, two π-donor hydrogen bonds with Trp88, Tyr64, three π-alkyl interactions with Leu36, Ala105, Leu110 residues. Dioxinodehydroeckol demonstrated five conventional hydrogen bonds with Thr75, Thr115, Trp88, Leu110, Arg61, π-π stacked, π-π T-shaped, amide-π stacked interactions with Gly38, Phe101, Trp88, van der Waals interaction with Trp60, π-anion interaction with Asp73, three π-donor hydrogen bonds with Trp88, Tyr56, Tyr64, two π-sigma interactions with Leu36, Val76, π-sulfur interaction with Cys79, three π-alkyl interactions with Leu110, Ala105, Ala127 interactions. Phlorofucofuroeckol A exhibited six conventional bond interactions with Gln94, Pro85, Glu145, Gln81, His78, Ser77, a π-π stacked interaction with Phe 87, and a π-sigma interaction with Ile86. Similarly, phlorofucofuroeckol B formed two conventional hydrogen bonds with Gln81, Ser77, a π-π stacked interaction with Phe 87, and van der Waals interaction with Gln94. Triphloroethol A formed six conventional hydrogen bonds with Thr75, Thr115, Trp88, Leu110, Trp60, Arg61, and π-π stacked, π-π T-shaped, amide-π stacked interactions with Gly101, Trp88, Gly 38, three π-donor hydrogen bonds with Trp88, Tyr56, Tyr64, three π-sigma interactions with Leu36, Ala127, Val76, π-sulfur interactions with Cys79, π-alkyl interactions with Leu110, Ala105. In contrast, phloroglucinol exhibited five conventional hydrogen bonds with Thr75, Thr115, Ser129, Trp60, and Tyr93, π-anion interaction with Asp73, and two π-alkyl interactions with Leu110 and Ala105 residues.

Docking analysis between MvfR and phlorotannins revealed binding free energies ranging from − 4.898 to -9.292 kcal/mol. The interactions involved included hydrogen bonding, van der Waals forces, and diverse π-interactions such as π-sigma, π-alkyl, π-π stacked, and amide-π stacked interactions. 2-Phloroeckol demonstrated a strong binding affinity of -9.292 kcal/mol. Compounds, such as 7-phloroeckol, bifuhalol, difucol, dioxinodehydroeckol, diphlorethohydroxycarmalol, diphlorethol, eckol, fucodiphloroethol G, fucophlorethol A, phlorofucofuroeckol A, phlorofucofuroeckol B, and triphloroethol A, exhibited moderate binding free energies ranging from − 7.488 to -8.788 kcal/mol. However, phloroglucinol and isoliolide showed weak binding affinities of -4.898 and − 6.416 kcal/mol, respectively. 2-phloroeckol exhibited four conventional hydrogen bonds with the Leu207, Ala102, Asp264, and Thr265, van der Waals interaction with Leu197, carbon-hydrogen bond with Thr265, four π-sigma interactions with Leu208, Ile236, Ala168, Ile149, amide-π stacked with Lys167, three π-alkyl interactions with Leu207, Ile236, Pro238 residues. Meanwhile, phloroglucinol formed a conventional hydrogen bond with Leu254, π-π stacked interactions with the Tyr258, π-donor hydrogen bond with Tyr258, π-sigma interaction with Leu189, two π-alkyl interactions with Val170 and Leu183 residues.

The PqsB and phlorotannin exhibited binding free energies between − 4.994 and − 10.2 kcal/mol. These complexes were stabilized by multiple interactions, including conventional hydrogen bonds, π-sigma, π-π stacked, π-alkyl, van der Waals, carbon-hydrogen bonds, π-π T-shaped, alkyl, and π-donor hydrogen bonds. Compared to other phlorotannins, 2-phloroeckol, 7-phloroeckol, diphlorethohydroxycarmalol, fucodiphloroethol G, fucophlorethol A, phlorofucofuroeckol A, and phlorofucofuroeckol B showed low binding free energies ranging from − 9.627 to -10.2 kcal/mol. Bifuhalol, difucol, dioxinodehydroeckol, diphlorethol, eckol, and triphloroethol A exhibited moderate binding free energies between − 7.05 and − 8.765 kcal/mol. Phloroglucinol and isoliolide again demonstrated the weakest binding interactions with PqsB (-4.994 and − 6.398 kcal/mol). Significantly, two phlorotannins formed π-π stacked interactions, specifically 2-phloroeckol and phlorofucofuroeckol B. 2-phloroeckol exhibited a conventional hydrogen bond with the Ala238 residue and π-π stacked interactions with the Phe274, π-sigma with Leu229, π-alkyl with Phe274 residue. Phlorofucofuroeckol B demonstrated four conventional hydrogen bonds with Pro271, Ser34, Ser32, and Met237, π-π stacked interaction with the Phe38, π-alkyl with the Met237 residue. However, phloroglucinol exhibited three conventional hydrogen bonds with Thr171, Arg168, and Leu172, a π-π T-shaped interaction with phe173, and a π-sigma interaction with the Leu172 residue.

RhlR interacted with different phlorotannins, and the complexes showed binding free energy ranging from − 4.28 to -7.752 kcal/mol. The complexes were found to be involved in many non-covalent interactions such as van der Waals, conventional hydrogen bonds, π-alkyl, carbon-hydrogen bonds, π-donor hydrogen bonds, alkyl, π-sigma, amide-π stacked, and π-cation interactions. 2-phloroeckol, 7-phloroeckol, diphlorethohydroxycarmalol, fucodiphloroethol G, fucophlorethol A, phlorofucofuroeckol A, and phlorofucofuroeckol B revealed relatively stronger binding affinities with RhlR (-7.185 to -7.752 kcal/mol). Other phlorotannins, bifuhalol, difucol, dioxinodehydroeckol, diphlorethol, eckol, isoliolide, and triphloroethol A exhibited moderate binding free energies ranging from − 5.015 to -6.377 kcal/mol. In contrast, phloroglucinol presented a weaker interaction (-4.28 kcal/mol). Phlorofucofuroeckol B formed two conventional hydrogen bonds with Lys228, Ser198, π-cation with Lys228, π-sigma with Thr229, alkyl and π-alkyl interaction with Ala232 residues. while phloroglucinol created four conventional hydrogen bonds with the Arg55, Thr58, Asn76, and Ala79 residues, respectively.

The binding interaction of acyl-homoserine-lactone synthase with phlorotannins exhibited binding free energy ranging from − 4.211 to -8.638 kcal/mol. These acyl-homoserine-lactone synthase-phlorotannins complexes formed various interactions, including conventional hydrogen bonds, π-cation, carbon-hydrogen bonds, π-donor hydrogen bonds, π-alkyl, π-sigma, π-anion, alkyl, van der Waals, and π-π stacked interactions. Notably, 2-phloroeckol, fucodiphloroethol G, and phlorofucofuroeckol B demonstrated low binding free energy values from − 8.232 to -8.638 kcal/mol. 7-phloroeckol, bifuhalol, difucol, dioxinodehydroeckol, diphlorethohydroxycarmalol, diphlorethol, eckol, fucophlorethol A, isoliolide, phlorofucofuroeckol A, and triphloroethol A exhibited moderate energies ranging from − 6.049 to -7.922 kcal/mol. However, phloroglucinol presented a high binding energy of -4.211 kcal/mol. Only fucodiphloroethol G phlorotannin formed π-π stacked interactions. Fucodiphloroethol G formed six conventional hydrogen bonds with Ser109, Ile107, Arg30, Phe105, Thr144, and Thr145, π-π stacked interactions with Phe105, π-cation and π-anion interaction with Arg30, Glu171, π-alkyl interaction with Val26, two π-donor hydrogen bonds with Phe27, and Phe105. In contrast, phloroglucinol formed two conventional hydrogen bonds with Ile107 and Arg30 and a π-alkyl interaction with Val26.

### Molecular interactions of phlorotannins with cell surface components and biofilm-associated proteins

The docking result of phlorotannins against the cell surface components (PilY1 and flagellin) and biofilm-associated alginate biosynthesis protein (AlgX and PelA) was summarized in **Table S4**.

PilY1 interacted with phlorotannins, with the binding free energies ranging from − 5.28 to -10.24 kcal/mol. These complexes were stabilized by multiple non-covalent interactions such as conventional hydrogen bonds, carbon-hydrogen bonds, π-anion, van der Waals, alkyl, π-sigma, π-alkyl, π-π stacked, π-donor hydrogen bond, π-π T-shaped interactions. Among them, 2-phloroeckol, 7-phloroeckol, diphlorethohydroxycarmalol, fucodiphloroethol G, phlorofucofuroeckol A, and phlorofucofuroeckol B showed stronger binding affinity with binding free energy values ranging from − 9.522 to -10.24 kcal/mol. Bifuhalol, difucol, diphlorethol, eckol, fucophlorethol A, and triphloroethol A exhibited moderate binding (-6.551 to -8.125 kcal/mol), and phloroglucinol and isoliolide showed weaker binding with binding free energy as low as -5.28 kcal/mol. Significantly, three phlorotannins formed π-π stacked interactions, such as phlorofucofuroeckol A, phlorofucofuroeckol B, and difucol. Phlorofucofuroeckol A formed seven conventional bonds with Gly856, Leu849, Thr792, Pro791, Lys790, Leu1123, Arg848, π-π stacked interaction with Tyr653, π-sigma with Ile661, alkyl interaction with Ala794, carbon-hydrogen, and π-donor hydrogen bonds with Tyr653 residues. Phlorofucofuroeckol B interacted via four conventional hydrogen bonds with Val793, Tyr653, Thr792, Lys790, π-π stacked interaction with Tyr653, van der Waals interaction with Asp1045, carbon-hydrogen bond with Gly856, and two π-alkyl interactions with Leu657, Val734 residues. Difucol exhibited π-π stacking interactions with Tyr653, π-sigma interactions with Ile661, and two π-alkyl interactions with the Ala794 and Ala858 residues. While phloroglucinol showed five conventional hydrogen bonds with Asp891, His797, Asn890, Lys879, and Ala877, π-sigma with Ala877, π-π T-shaped with His797, π-alkyl with Ile876 residue.

The interactions of flagellin with phlorotannins exhibited binding free energies between − 4.441 and − 8.942 kcal/mol. These complexes were stabilized by various non-covalent interactions, including van der Waals, conventional hydrogen bonds, π-donor hydrogen bonds, π-sigma, π-alkyl, carbon-hydrogen bonds, amide-π stacked, π-anion, alkyl, and π-cation interactions. Notably, 2-phloroeckol showed a low binding free energy of -8.942 kcal/mol. Other phlorotannins, 7-phloroeckol, difucol, dioxinodehydroeckol, diphlorethohydroxycarmalol, diphlorethol, eckol, fucodiphloroethol G, fucophlorethol A, phlorofucofuroeckol A, phlorofucofuroeckol B, triphloroethol A, exhibited moderate binding free energies in the range of -6.157 to -7.795 kcal/mol. However, phloroglucinol, isoliolide, and bifuhalol showed relatively weaker interaction with binding free energies of -4.441, -5.459, and − 5.746 kcal/mol, respectively. 2-phloroeckol interacted via five conventional hydrogen bonds with Gln82, Gln83, Arg124, Thr128, Gln75, van der Waals interactions with Gly79, Thr130, π-donor hydrogen bond with Thr129, π-sigma with Thr76, π-alkyl with Ala80 residue. However, phloroglucinol formed four conventional hydrogen bonds with Thr171, Leu301, Ala247, and Asp304, two π-alkyl interactions with Lys246, Ala247, π-anion and π-cation interactions with Lys308 and Glu305 residues.

AlgX with phlorotannins showed binding free energies ranging from − 6.101 to -9.192 kcal/mol. These complexes exhibited various interactions such as van der Waals, conventional hydrogen bond, π-π stacking, and various other π-interactions. Significantly, 2-phloroeckol, diphlorethohydroxycarmalol, fucodiphloroethol G, phlorofucofuroeckol A, and phlorofucofuroeckol B exhibited the strongest binding interactions (-9 to -9.192 kcal/mol), and 7-phloroeckol, bifuhalol, difucol, dioxinodehydroeckol, diphlorethol, eckol, fucophlorethol A, and triphloroethol A showed moderate (-7.014 to -8.808 kcal/mol). In contrast, isoliolide and phloroglucinol had relatively weaker binding (-5.855 and − 6.101 kcal/mol). Notably, 2-phloroeckol, phlorofucofuroeckol A, 7-phloroeckol, and dioxinodehydroeckol formed π-π stacked interactions. 2-phloroeckol formed five conventional hydrogen bonds with Lys217, Lys223, Leu218, Lys56, Tyr57, π-π stacked and π-π T-shaped interactions with His175, Phe60, van der Waals interactions with Gly298, two carbon hydrogen bonds with Thr268, His176, π-sigma interaction with Gly296 residue. Phlorofucofuroeckol A demonstrated six conventional hydrogen bonds with Tyr77, Lys56, Phe299, Tyr328, Asp300, Asp329, π-π stacked and π-π T-shaped interactions with Phe60, Tyr328, a carbon-hydrogen bond with His327, two π-alkyl interactions with His327, and Phe60 residues. 7-phloroeckol exhibited six conventional hydrogen bonds with Tyr328, His327, Ser269, His175, Lys217, Lys223, π-π stacked interactions with Phe60, van der Waals with Ser216 residue. Dioxinodehydroeckol interacted via seven conventional hydrogen bonds, each with Ser269, Gly296, Tyr328, Phe299, Gly297, Asp300, Gln332, π-π stacked and π-π T-shaped interactions with His327 and Phe60, four carbon-hydrogen and π-donor hydrogen bonds with His175, Phe60, Thr268, and Gly298 residues. In contrast, phloroglucinol formed six conventional hydrogen bonds with His327, Arg74, His175, Tyr170, Gln112, Thr326, and a π-sigma interaction with the Ala325 residue.

The binding free energies between PelA and phlorotannins were found in the range of -4.489 to -9.515 kcal/mol, with multiple interactions stabilizing the complexes, including conventional hydrogen bonds, π-π stacked, π-alkyl, van der Waals, alkyl, and π-cation. Notably, 2-phloroeckol, 7-phloroeckol, diphlorethohydroxycarmalol, and phlorofucofuroeckol B had higher binding affinities (-9.012 to -9.515 kcal/mol). Dioxinodehydroeckol, diphlorethol, eckol, fucodiphloroethol G, fucophlorethol A, phlorofucofuroeckol A, and triphloroethol A exhibited moderate binding free energy values between − 7.713 and − 8.99 kcal/mol. Phloroglucinol, isoliolide, bifuhalol, and difucol exhibited weaker binding interactions (-4.489 to -6.839 kcal/mol). Notably, two phlorotannins formed π-π stacked interactions, including 2-phloroeckol and difucol. 2-phloroeckol exhibited five conventional hydrogen bonds with Glu233, Arg232, Ser219, Trp224, Pro289, π-π stacked interaction with Tyr261, two carbon hydrogen bonds with Pro235, Trp224, two π-anion interactions with Asp238, Glu218, π-alkyl with Val234. Difucol formed three conventional hydrogen bonds with Asp238, Asp237, and Glu203, and a π-π stacked interaction with the Trp241 residue. However, phloroglucinol formed three conventional hydrogen bonds with Arg232, Asp225, and Trp224 residues, π-anion with Asp238, and π-alkyl interaction with Pro235 residue.

### Molecular interactions of phlorotannins with proteolytic enzymes and iron acquisition proteins

Similarly, proteolytic enzymes (serralysin, elastase, and LasA) and iron acquisition proteins (Fe (3+)-pyochelin receptor and ferripyoverdine receptor) were docked with fifteen phlorotannins (**Table S5**).

The docking analysis of serralysin with fifteen phlorotannins revealed binding free energies ranging from − 4.686 to -9.765 kcal/mol. Notably, 2-phloroeckol, 7-phloroeckol, diphlorethohydroxycarmalol, fucodiphloroethol G, phlorofucofuroeckol A, and phlorofucofuroeckol B exhibit the lowest binding free energy (ranging from − 9.156 to -9.765 kcal/mol), stabilized by a rich network of interactions such as hydrogen bonds, π-cation, π-donor hydrogen bonds, π-anion, alkyl, and π-π stacking. Phlorotannins like Bifuhalol, difucol, dioxinodehydroeckol, eckol, fucophlorethol A, and triphloroethol A showed moderate binding energies (between − 7.23 and − 8.412 kcal/mol), and phloroglucinol, isoliolide, and diphlorethol had relatively weaker interactions with higher binding energies (-4.686 and − 6.753 kcal/mol). In-depth interaction analysis showed fucodiphloroethol G formed six conventional hydrogen bonds (with Ser104, Phe111, Ile109, Thr251, Asn249, and Thr252), π-alkyl interaction with Val54, and π-π stacked interaction with His110. In contrast, phloroglucinol engaged in π-π stacked, π-π T-shaped, and amide-π stacked interactions with Trp217, Gly188, and His186, along with conventional hydrogen bonds with Met214, Tyr216, and Asp 207.

Elastase displayed binding affinities with phlorotannins in the range of −  4.067 to −  9.939 kcal/mol. The strongest interactions were observed for 2-phloroeckol, 7-phloroeckol, phlorofucofuroeckol A, and phlorofucofuroeckol B (−  9.007 to -9.939 kcal/mol), while moderate interactions were seen with compounds like dioxinodehydroeckol, diphlorethohydroxycarmalol, and triphloroethol A. Weaker interactions were noted for phloroglucinol, isoliolide, difucol, and bifuhalol (−  4.067 to −  6.867 kcal/mol). Interestingly, π-π stacking was a recurrent motif in eight phlorotannins, including 7-phloroeckol, diphlorethol, and fucophlorethol A. 7-phloroeckol formed hydrogen bonds with residues such as Leu153 and Tyr155, and π-interactions with His144. Similarly, dioxinodehydroeckol and triphloroethol A formed multiple hydrogen and π-π interactions with His144 and Glu141. Fucophlorethol A and bifuhalol displayed extensive interaction networks involving both π-stacking and hydrogen bonding, while phloroglucinol formed π-donor and π-π interactions with His144 and Tyr155.

Protease LasA and phlorotannin complexes exhibited binding energies ranging from − 4.94 to −  9.466 kcal/mol. These complexes were stabilized by various interactions, including conventional hydrogen bonds, π-π stacked, π-π T-shaped, and π-alkyl interactions. Compounds such as 2-phloroeckol, 7-phloroeckol, fucodiphloroethol G, and fucophlorethol A showed the strongest binding affinities (−  9.042 to -9.466 kcal/mol). In comparison, moderate interactions (−  7.093 to −  8.756 kcal/mol) were found with bifuhalol, difucol, dioxinodehydroeckol, diphlorethohydroxycarmalol, diphlorethol, eckol, phlorofucofuroeckol A, phlorofucofuroeckol B, and triphloroethol A. High binding energies (indicative of weak binding) were seen with phloroglucinol and isoliolide (−  4.94 to −  5.561 kcal/mol). 2-phloroeckol interacted via conventional hydrogen bonds with Asn20, and Asp36, and π-π stacking with Trp41, and Tyr151. Phlorofucofuroeckol B engaged in multiple hydrogen bonds (with Ser115, Tyr151, Asp36, His23, Gln66, Glu112), three π-π stacking interactions with His122, His81, and Tyr80. In contrast, phloroglucinol showed fewer interactions, including hydrogen bonds with Asn25 and Gln66, and one π-π stacked interaction with Tyr80.

Iron acquisition protein Fe^3+^-pyochelin receptor and phlorotannin complex showed binding free energy ranging from − 5.897 to -12.24 kcal/mol. 2-phloroeckol, 7-phloroeckol, diphlorethohydroxycarmalol, and fucodiphloroethol G exhibited strong binding affinities (ranging − 11.05 to −  12.24 kcal/mol). Bifuhalol, difucol, dioxinodehydroeckol, diphlorethol, eckol, fucophlorethol A, phlorofucofuroeckol A, phlorofucofuroeckol B, and triphloroethol A showed moderate binding energies from − 8.066 to -10.95 kcal/mol, while phloroglucinol and isoliolide had higher binding energies and thus weaker interactions (-5.897 and − 7.287 kcal/mol, respectively). 2-Phloroeckol engaged in four conventional hydrogen bonds with Glu708, Gln695, Arg705, Asn686, π-anion interaction with Asp100, π-alkyl interaction with Pro113, and π-donor interaction with Tyr232. Despite weaker binding, phloroglucinol formed seven hydrogen bonds, including with Arg717 and Glu204, along with π-anion and π-alkyl interactions with Leu91 and Glu204.

Ferripyoverdine receptor interaction with the phlorotannins resulted in binding-free energies from − 5.402 to −  1.6 kcal/mol. Dioxinodehydroeckol, eckol, fucodiphloroethol G, and triphloroethol A had the lowest binding free energies from − 10.91 to −  1.6 kcal/mol. Moderate binding affinities (−  8.314 to −  9.938 kcal/mol) were shown by 2-phloroeckol, 7-phloroeckol, bifuhalol, difucol, diphlorethohydroxycarmalol, diphlorethol, fucophlorethol A, phlorofucofuroeckol A, and phlorofucofuroeckol B. Phloroglucinol and isoliolide again exhibited the weakest binding profile (−  5.402 and − 6.821 kcal/mol). Dioxinodehydroeckol showed a rich interaction profile including eight conventional hydrogen bonds (with Tyr789, Asn783, Asp803, Asn290, Gln316, Phe180, Gly181, and Asp764), π-anion interaction with Asp187, π-alkyl interaction with Leu762, and π-donor hydrogen bonds with Lys318 and Asn782. In contrast, phloroglucinol formed only two conventional hydrogen bonds with Arg768 and Ser718, and one π-alkyl interaction with Lys720.

### Molecular interactions of phlorotannins with host adhesion and toxin-producing virulence proteins

The docking interactions between host adhesion proteins (PA-I and PA-IIL) and toxin-producing proteins (PhzM and VgrG2b) with phlorotannins were summarized in **Table S6**.

The binding free energy of PA-I with phlorotannin ranged from − 4.223 to − 8.011 kcal/mol. These complexes engaged in multiple interactions such as conventional hydrogen bonds, π-alkyl, π-donor hydrogen bonds, π-cation, π-π T-shaped, amide-π stacked, van der Waals, π-sigma, carbon-hydrogen bonds, and π-π stacked interactions. Among the phlorotannins, **7-**phloroeckol, diphlorethohydroxycarmalol, fucodiphloroethol G, phlorofucofuroeckol A, and phlorofucofuroeckol B exhibited strong binding affinity in the range of -7.053 to −  8.011 kcal/mol. Moderated binding affinities were observed for 2**-**phloroeckol, bifuhalol, difucol, dioxinodehydroeckol, diphlorethol, eckol, fucophlorethol A, and triphloroethol (between − 5.496 and − 6.844 kcal/mol). Phloroglucinol and isoliolide showed the weakest binding, with binding free energies of −  4.233 and − 4.829 kcal/mol, respectively. Notably, phlorofucofuroeckol B interacted strongly via one conventional hydrogen bond with Gly43, two π-π stacked, and a π-π T-shaped interaction with Trp42, Trp33, two π-alkyl interactions with Lys41, Trp33, a carbon-hydrogen bond, and a π-donor hydrogen bond with Lys41, Gln40. Conversely, phloroglucinol, despite forming six conventional hydrogen bonds with Tyr36, Thr104, Asn107, Asp100, His50, Gln53, and one π-π T-shaped interaction with Tyr36, had a weaker binding profile.

Binding free energies for phlorotannin complexes with PA-IIL spanned − 4.193 to -8.658 kcal/mol. These complexes demonstrated multiple interactions, van der Waals, conventional hydrogen bonds, carbon-hydrogen bonds, π-anion, alkyl, π-alkyl, π-donor hydrogen bond, π-sigma, π-π T-shaped, and amide-π stacked interactions. Phlorofucofuroeckol A and phlorofucofuroeckol B exhibited the lowest binding free energies of -8.658 and − 8.527 kcal/mol, respectively. 2-phloroeckol, 7-phloroeckol, bifuhalol, difucol, dioxinodehydroeckol, diphlorethohydroxycarmalol, diphlorethol, eckol, fucodiphloroethol G, fucophlorethol A, and triphloroethol A had moderate binding free energy ranging from − 6.132 to -7.908 kcal/mol. The weakest binding was seen with phloroglucinol and isoliolide (-4.193 and − 4.99 kcal/mol). Detailed interaction analysis revealed that phlorofucofuroeckol A formed eight conventional hydrogen bonds with Ser22, Ser23, Thr45, Asp104, Asp101, Glu95, Asp99, Thr98, and one carbon-hydrogen bond with Gly97. In contrast, phloroglucinol showed fewer contacts, forming three conventional hydrogen bonds with Asp75, Leu76, and Gln3 and one π-sigma interaction with Leu76.

Phenazine-1-carboxylate N-methyltransferase showed binding free energies between − 5.066 and − 11.27 kcal/mol. The binding was facilitated by conventional hydrogen bonds, carbon-hydrogen bonds, π-sigma, π-alkyl, π-donor hydrogen bonds, π-cation, π-π T-shaped, van der Waals, and alkyl interactions. Compared to other phlorotannins, 2-phloroeckol, 7-phloroeckol, diphlorethohydroxycarmalol, fucodiphloroethol G, phlorofucofuroeckol A, and phlorofucofuroeckol B showed the strongest binding affinity with energies between − 9.161 and − 11.27 kcal/mol. A moderate group (bifuhalol, difucol, dioxinodehydroeckol, diphlorethol, eckol, fucophlorethol A, and triphloroethol A) had binding free energies between − 7.35 and − 8.621 kcal/mol, while phloroglucinol and isoliolide had the weakest binding (-5.066 and − 6.365 kcal/mol). Phlorofucofuroeckol B engaged in nine conventional hydrogen bonds with Gly176, Gly179, Glu180, Ser178, Gly143, Gly201, Tyr133, Arg241, Ser240, van der Waals interaction with Gly204, one π-sigma interaction with Val205, three π-alkyl interactions with Leu147, Met150, Val205 interactions. Meanwhile, phloroglucinol formed four conventional hydrogen bonds with Ser240, Leu181, Gly176, Glu180, and one π-π T-shaped interaction with Phe157.

The binding free energy for the VgrG2b with phlorotannins ranged from − 4.888 to -9.779 kcal/mol. The interaction repertoire included conventional hydrogen bond, π-sulfur, π-alkyl, carbon-hydrogen bond, π-sigma, alkyl, van der Waals, π-donor hydrogen bond, π-π T-shaped, π-cation, π-anion, π-π stacked, amide-π stacked interactions. Fucophloroethol A and phlorofucofuroeckol B exhibited low binding free energy of -9.708 and − 9.779 kcal/mol, respectively. Most other phlorotannins (2-phloroeckol, 7-phloroeckol, bifuhalol, difucol, dioxinodehydroeckol, diphlorethohydroxycarmalol, diphlorethol, eckol, fucodiphloroethol G, fucophlorethol A, and triphloroethol A) showed moderate affinities between − 7.399 and − 8.975 kcal/mol. Phloroglucinol and isoliolide again demonstrated weak binding (-4.888 and − 6.478 kcal/mol). Phlorofucofuroeckol B formed eight conventional hydrogen bonds with Arg959, Gln960, Asp965, Gln966, Arg970, Glu972, Tyr980, Gln956, one π-π stacked, and π-π T-shaped interactions with Tyr980, one π-donor hydrogen bond with Tyr955, and one π-alkyl interaction with Lys975. The phloroglucinol formed six conventional hydrogen bonds with Gln957, Tyr902, Val949, Leu950, Ala954, Ala905 and two π-alkyl interactions with Met904, Pro906.

Across all 18 virulence proteins, 2-phloroeckol, 7-phloroeckol, difucol diphlorethohydroxycarmalol, fucodiphloroethol G, phlorofucofuroeckol A, and Phlorofucofuroeckol B consistently exhibited a strong binding affinity and rich interaction profiles. These interactions, involving hydrogen bonding and multiple π-type contacts, suggest their potential to effectively occupy and interact with the phlorotannins within the binding pockets of virulence proteins. In contrast, phloroglucinol and isoliolide showed weaker binding across all targets.

**Fig. 4:**
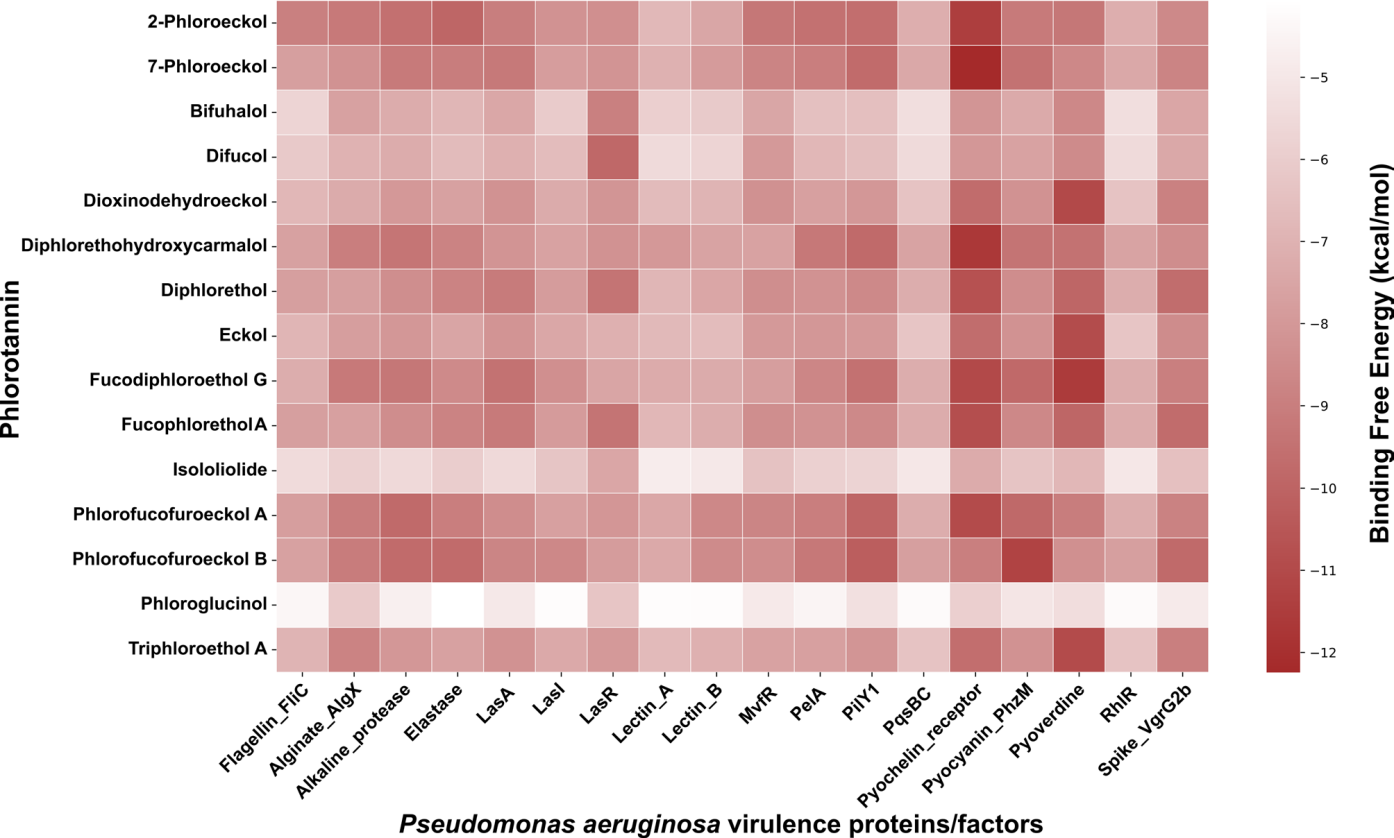
Heatmap illustrating the intensity of the lowest binding free energies (kcal/mol) of various phlorotannin compounds with *Pseudomonas aeruginosa* target proteins. Color gradient illustrates the strength of the interaction, with dark brown color showing a more negative value of binding free energy (stronger interactions) and light brown color corresponding to the lower binding free energy (weaker interactions). The label of the color gradient highlights the numerical range of binding free energy values

Figure 4 depicts a heatmap, showing overall binding affinity patterns of docked virulence protein-phlorotannin complexes. The phlorotannins 2-phloroeckol, 7-phloroeckol, diphlorethohydroxycarmalol, fucodiphloroethol G, phlorofucofuroeckol A, and phlorofucofuroeckol B consistently exhibited strong binding affinities (dark-brown boxes) across various virulence proteins, indicating stronger interactions. Phlorotannins such as bifuhalol, difucol, dioxyindodehydroeckol, eckol, fucophlorethol A, and triphloroethol showed moderate binding affinities (light-brown boxes) with most of the virulence proteins. In contrast, phloroglucinol and isololiolide were found to interact weakly (nearly white color boxes) with all the proteins of *P. aeruginosa*. Here, the intensity of color is directly proportional to the negative value of binding free energies. The dark-brown color represents the strong interactions, the light-brown color depicts intermediate binding energy values, and the white color represents the lowest. Detailed 2D interaction diagrams of all protein-ligand complexes generated in this study were provided in the **Supplementary Figures ****S1****-S18**.

**Fig. 5:**
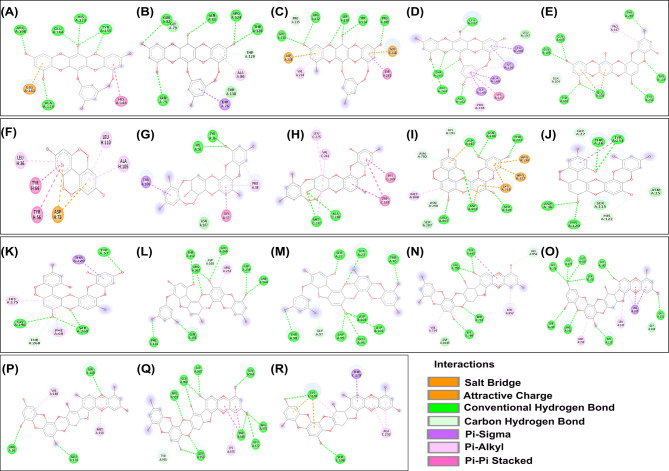
2D interaction diagrams showing the molecular interactions and key residues involved in the interactions between *P. aeruginosa* virulence factors and the phlorotannin compound, showing the strongest interaction. **A–D** 2-Pholoroeckol in complex with elastase, flagellin, PelA, and multiple virulence factor regulator MvfR respectively, **E** 7-phloroeckol with Fe ^3+^pyochelin receptor, **F** Difucol with transcriptional activator protein LasR, **G–H** Diphlorethohydroxycarmalol in complex with PA-I galactophilic lectin, and 2-heptyl-4(1 H)-quinolone synthase subunit PqsB, **I–K** Fucodiphloroethol G with ferripyoverdine receptor, protease LasA, and alginate biosynthesis protein AlgX respectively, **L–M** Phlorofucofuroeckol A with serralysin, and fucose-binding lectin PA-IIL respectively, **N–R** Phlorofucofuroeckol B in complex with type IV pilus biogenesis factor PilY1, phenazine-1-carboxylate N-methyltransferase, acyl-homoserine-lactone synthase, type VI secretion system spike protein VgrG2b, HTH-type quorum-sensing regulator RhlR respectively. Color boxes, along with the text labels, list the interaction types

For each virulence protein, the 2D interaction plots of the docking complexes with the phlorotannins exhibiting the strongest binding affinities are illustrated in Fig. 5. The plots highlighted the key amino acid residues involved in ligand binding and the types of interactions that stabilized the phlorotannins within the active site of virulence proteins. The majority of complexes exhibited multiple conventional hydrogen bonds, which were fundamental in anchoring the ligands through polar interactions. Apart from this, multiple π interactions such as π-π stacking, π-alkyl, π-anion, π-cation, π-sigma, and π-π T-shaped were commonly observed, facilitating aromatic and hydrophobic stabilization of phlorotannins within the protein active site. Additionally, salt bridges and π-sulfur interactions suggested that the formation of electrostatic and sulfur-mediated non-covalent interactions might play a significant role in binding affinity. Other interactions, such as carbon-hydrogen bonds, π-π donor hydrogen bonds, and amide-π stacking, might contribute to the structural integrity of the complex. A complete summary of the binding free energies for the most strongly interacting virulence factor–phlorotannin complexes was provided in Table 1.

**Table 1:** The molecular docking results of *P. aeruginosa* virulence factors and the phlorotannin compound show the strongest interaction. For each ligand-protein pair, the binding free energy (in kcal/mol), types of molecular interactions, and key interacting residues are listed

Phlorotannins	Virulence proteins	Binding free energy (kcal/mol)	Interaction types
2-Phloroeckol	Elastase	− 9.939	Conventional hydrogen bond, π-π T-shaped, π-anion
	Flagellin	− 8.942	Van der Waals, π-sigma, conventional hydrogen bond, π-Alkyl, π-donor hydrogen bond
	PelA	− 9.515	Conventional hydrogen bond, π-πstacked, carbon-hydrogen bond, π-alkyl, π-Anion
	Multiple virulence factor regulator MvfR	− 9.292	Van der Waals, π-sigma, conventional hydrogen bond, amide-π stacked, carbon-hydrogen bond, π-alkyl
7-Phloroeckol	Fe (3+)-pyochelin receptor	− 12.24	Conventional hydrogen bond, π-donor hydrogen bond, π-anion, π-alkyl
Difucol	Transcriptional activator protein LasR	− 9.865	Salt bridge, alkyl, conventional hydrogen Bond, π-alkyl, carbon-hydrogen bond
Diphlorethohydroxycarmalol	PA-I galactophilic lectin	− 8.011	Van der Waals, π-sigma, conventional hydrogen bond, amide-π stacked, carbon-hydrogen bond, π-alkyl
	2-heptyl-4(1 H)-quinolone synthase subunit PqsB	-10.2	Conventional hydrogen bond, π-πT-shaped, alkyl
Fucodiphloroethol G	Ferripyoverdine receptor	− 11.6	Van der Waals, π-Anion, conventional hydrogen bond, π-donor hydrogen bond, carbon-hydrogen bond, π-alkyl, π-cation
	Protease LasA	− 9.466	Van der Waals, π-donor hydrogen bond, conventional hydrogen bond, π-πT-shaped, carbon-hydrogen bond
	Alginate biosynthesis protein AlgX	− 9.192	Conventional hydrogen bond, π-sigma, carbon-hydrogen bond, π-alkyl
Phlorofucofuroeckol A	Serralysin	− 9.765	Conventional hydrogen bond, carbon-hydrogen bond, π-alkyl
	Fucose-binding lectin PA-IIL	− 8.658	Conventional hydrogen bond, carbon-hydrogen bond
Phlorofucofuroeckol B	Type IV pilus biogenesis factor PilY1	− 10.24	Van der Waals, π-π stacked, conventional hydrogen bond, π-alkyl, carbon-hydrogen bond
	Phenazine-1-carboxylate N-methyltransferase	− 11.27	Van der Waals, π-sigma, conventional hydrogen bond, π-alkyl
	Acyl-homoserine-lactone synthase	− 8.638	Conventional hydrogen bond, π-Alkyl
	Type VI secretion system spike protein VgrG2b	− 9.779	Conventional hydrogen bond, π-π T-shaped, π-donor hydrogen bond, π-alkyl, π-π stacked
	HTH-type quorum-sensing regulator RhlR	− 7.752	Conventional hydrogen bond, alkyl, π-cation, π-alkyl, π-sigma

**Fig. 6:**
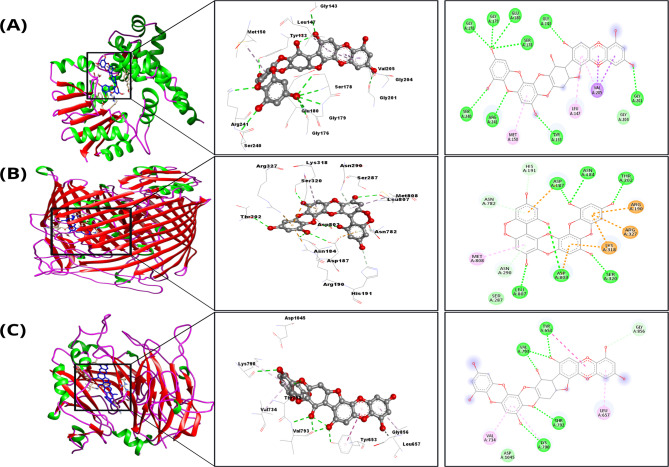
Molecular docking analysis of the *Pseudomonas aeruginosa* virulence factor and phlorotannin showing the best interactions. The figures from left to right in the panel show cartoon (red: beta-sheets, green: alpha-helices, pink: turns/loops, and blue: ligand) of protein-ligand complexes, interacting residues forming the core of the active site (Ligand: Ball-socket, and Protein: lines), and 2D interaction plots showing the interacting residues and interaction types (dark-green: Conventional H-bond, light-green: van der Waal interaction, orange: Pi-cation/anion interactions, dark-purple: Pi-Sigma, and light-purple: Pi-alkyl interaction). **A** Phenazine-1-carboxylate N-methyltransferase with phlorofucofuroeckol B. **B** Ferripyoverdine receptor bound to fucodiphloroethol G. and **C** Type IV pilus biogenesis factor PilY1 in complex to phlorofucofuroeckol B

Figure 6 showed the surface representation (red: beta-sheets, green: alpha-helices, pink: turns/loops, and blue: ligand) of protein-ligand complexes, interacting residues forming the core of active site (ligand: Ball-socket, and protein: lines), and 2D interaction plots showing the interacting residues and interaction types (dark-green: Conventional H-bond, light-green: Van der waal’s interaction, orange: Pi-cation/anion interactions, dark-purple: Pi-Sigma, and light-purple: Pi-alkyl interaction) of three protein-phlorotannin complexes, that exhibited the highest binding affinities and relatively rich interaction profiles across all the docking complexes. These complexes were selected for molecular dynamics simulations. Figure 6A depicts fucodiphloroethol G in complex with ferripyoverdine receptor, showing fucodiphloroethol G positioned between the alpha-helices and beta-sheets of the active site of the protein. This protein was found to form several polar and hydrophobic contacts with fucodiphloroethol G, resulting in a binding free energy of -11.6 kcal/mol. Similarly, Phlorofucofuroeckol B strongly interacted with PilY1 and phenazine-1-carboxylate N-methyltransferase (Fig. 6B and C, respectively), resulting in binding free energies of -10.24 kcal/mol and − 11.27 kcal/mol, respectively.

### Pharmacokinetic (ADMET) profiling of phlorotannins

The pharmacokinetic parameters, including absorption, distribution, metabolism, excretion, and toxicity, were evaluated for all fifteen phlorotannins using the pkCSM-pharmacokinetics tool. This* in silico* study provided a comprehensive understanding of the safety profiles of each phlorotannin. It aided in identifying phlorotannins with optimal bioavailability, low toxicity, and favourable pharmacological properties for further analysis. **Table S7** summarizes the pharmacokinetic parameters of phlorotannins, while **Table S8** outlines the toxicity analysis of phlorotannins.

### Absorption

The water solubility of the fifteen phlorotannins, expressed in log mol/L, ranged from − 3.265 to − 1.067. Among them, phloroglucinol and isoliolide had the highest water solubility of − 1.067 and − 1.401 log mol/L, indicating a significantly greater solubility than other compounds. In contrast, bifuhalol, dioxinodehydroeckol, and diphlorethol exhibit low water solubility with values of − 3.006, − 3.008, and − 3.265 log mol/L. The Caco-2 permeability of all phlorotannins was observed, ranging from − 0.131 to 1.217 log Papp. Phloroglucinol and isoliolide exhibited higher Caco-2 permeability, with values of 1.094 and 1.217 log Papp, respectively, indicating a high intestinal absorption potential. Other phlorotannins, 7-phloroeckol, eckol, fucodiphloroethol G, fucophlorethol A, phlorofucofuroeckol A, phlorofucofuroeckol B, also showed higher permeability with values ranging from 0.542 to 0.503 log Papp. On the other hand, dioxinodehydroeckol and diphlorethohydroxycarmalol showed low permeability with values of -0.176 and − 0.131 log Papp. The intestinal absorption of all phlorotannins varied significantly, ranging from 56.087 to 100%. 2-phloroeckol and dioxinodehydroeckol demonstrated the highest intestinal absorption at 94.402% and 100%, respectively. In contrast, fucophlorethol A 56.087% and difucol 57.858% exhibited lower absorption. Regarding P-glycoprotein interactions, phloroglucinol and isoliolide were the only compounds predicted not to be substrates for P-glycoprotein, which enhances their bioavailability. In contrast, the other thirteen phlorotannins are anticipated to be substrates for P-glycoprotein, suggesting that the efflux mechanism diminishes their bioavailability. Phlorotannins demonstrate different inhibitory effects on P-glycoproteins I and II. Out of fifteen phlorotannin compounds, only five compounds inhibit P-glycoprotein (I) Similarly, ten compounds show inhibition of P-glycoprotein (II) Notably, bifuhalol, difucol, diphlorethol, isololiolide, and phloroglucinol did not show any inhibition against both P-glycoproteins I and II.

### Distribution

The study-state volume of distribution (VDss) values varied among the fifteen phlorotannins, suggesting differences in their distribution across human tissues. Bifuhalol exhibited the greatest distribution potential with a VDss of 0.378 log L/kg, followed by difucol, diphlorethol, and fucophloroethol A, which also exhibited positive log VDss values ranging from 0.112 to 0.183. In contrast, dioxinodehydroeckol had the lowest distribution potential with a VDss of − 1.263 log L/kg. The fraction unbound (Fu) values showed which phlorotannin compound is free in plasma versus bound to plasma proteins. Isoliolide and phloroglucinol had the highest values of 0.611 and 0.553, suggesting that these compounds were freely available in plasma and accessible for activity or clearance. By contrast, bifuhalol, dioxinodehydroeckol, and diphlorethol had low Fu values ranging from 0.135 to 0.143, indicating these compounds might bind to plasma proteins.

### Metabolism

The inhibitory activity of phlorotannins against cytochrome P450 enzymes was evaluated to assess their potential and metabolic drug-drug interactions. All phlorotannin compounds were predicted not to be substrates for the cytochrome P450 enzymes CYP2D6 and CYP3A4. The inhibitory activity of phlorotannin against CYP1A2, CYP2C19, CYP2C9, CYP2D6, and CYP3A4 was tested. Most phlorotannins exhibited no inhibitory activity; however, bifuhalol, difucol, dioxinodehydroeckol, and triphloroethol A demonstrated inhibitory activity against the CYP1A2 enzyme. Dioxinodehydroeckol also showed inhibitory activity against CYP2C19 and CYP3A4. Eckol, fucophlorethol A, and triphloroethol A inhibited the CYP2C9 enzyme. Overall, the phlorotannins that did not inhibit the CYP enzymes were considered more favourable due to their lower risk of metabolic drug-drug interactions.

### Excretion

Compared to other phlorotannins, isoliolide had the highest clearance value of 1.042 log ml/min/kg, suggesting it would be removed from systemic circulation more quickly than the others. Diphlorethohydroxycarmalol showed the lowest clearance value, 0.433 log ml/min/kg, indicating a slower elimination rate. All phlorotannin compounds were predicted to be non-substrate compounds for the renal OCT2. The absence of OCT2 interaction suggests a lower risk of drug-drug interactions with recognized OCT2 inhibitors.

### Toxicity

Only four phlorotannins, bifuhalol, difucol, dioxinodehydroeckol, and diphlorethol, exhibited AMES toxicity. The maximum tolerated dose (MTD) was investigated to determines their potential toxicity. The threshold value of 0.477 log mg/kg/day is used to differentiate the toxic and nontoxic compounds. Eleven phlorotannins, including 2-phloroeckol, 7-phloroeckol, diphlorethohydroxycarmalol, eckol, fucodiphloroethol G, fucophlorethol A, phlorofucofuroeckol A, phlorofucofuroeckol B, phloroglucinol, and triphloroethol A, values ranging from 0.351 to 0.456, suggested a lower risk of toxicity. All fifteen compounds were predicted to be non-inhibitors of hERG (I) In contrast, only five phlorotannins, such as bifuhalol, difucol, diphlorethol, isoliolide, and phloroglucinol, were predicted to be non-inhibitors of hERG (II) Significantly, isoliolide and phloroglucinol were the only compounds that were non-inhibitors of both hERG I and II. The oral acute toxicity of phlorotannin was predicted in rats, ranging from 1.89 to 2.555 mol/kg. Phloroglucinol and diphlorethol showed the lowest LD50 values of 1.89 and 1.891 mol/kg, indicating higher toxicity than the others. By comparison, fucodiphloroethol A had a high LD50 value of 2.555 mol/kg, suggesting lower oral toxicity. The lowest observed adverse effect level (LOAEL) values of phlorotannins for oral chronic toxicity in rats ranged from 2.241 to 5.482 log mg/kg_bw/day. The phlorofucofuroeckol A, phlorofucofuroeckol B, fucodiphloroethol G, diphlorethohydroxycarmalol, and 7-phloroeckol had the highest LOAEL values, ranging from 5.141 to 5.482 log mg/kg_bw/day. All phlorotannin compounds were predicted to be non-hepatotoxic. Compared to other phlorotannins, only isoliolide was predicted to be a potential skin sensitizer.

The ADMET analysis of the phlorotannin revealed that most phlorotannins exhibit favourable pharmacokinetic parameters. Phloroglucinol, isoliolide, exhibited great water solubility, Caco2 permeability, and plasma availability, and did not interact with P-glycoproteins and cytochrome P450 enzymes. All phlorotannins were predicted not to be substrates of renal OCT2. Furthermore, the majority of the phlorotannins were found to be non-toxic, lack hepatotoxicity, and have a tolerable level of oral toxicity.

### Status of mutagenicity of phlorotannins

The mutagenic potential of selected phlorotannins was analyzed using the VEGA (Q)SAR platform, which combines various prediction models, including Caesar, SarPy/IRFN, ISS, KNN Read-Across, and a consensus model. **Table S9** summarizes the predictions and reliability scores, such as the consensus score (CS) and global applicability domain index (GADI).

The consensus model integrated multiple prediction models, which predicted 2-phloroeckol, 7-phloroeckol, difucol, diphlorethohydroxycarmalol, eckol, diphlorethol, fucodiphloroethol G, fucophlorethol A, isololiolide, phlorofucofuroeckol A, phlorofucofuroeckol B, phloroglucinol, and triphloroethol A to be non-mutagenic. Among these, phloroglucinol has high confidence across all models, with maximum reliability scores (CS and GADI = 1), and exhibited no structural alarms for mutagenicity.

The five phlorotannins 2-phloroeckol, 7-phloroeckol, fucodiphloroethol G, diphlorethohydroxycarmalol, and fucophlorethol A were predicted as non-mutagenic by the Caesar, SarPy/IRFN, ISS, and KNN Read-Across models. The reliability score indicated low to moderate confidence in these predictions. Difucol, eckol, diphlorethol, isololiolide, and triphloroethol A exhibited mutagenicity in only one model, while the remaining three models predict these compounds as non-mutagenic with a reliability score ranging from low to moderate. Furthermore, phlorofucofuroeckol A and phlorofucofuroeckol B were predicted to be non-mutagenic by two models with a low to moderate reliability score.

In contrast, the consensus model and several individual models predicted bifuhalol and dioxinodehydroeckol as mutagenic. Bifuhalol was forecasted to be mutagenic by Caesar and KNN/Read-Across, showing a moderate to high-reliability score. Dioxinodehydroeckol was forecasted to be mutagenic by Caesar, ISS, and KNN/Read-Across models with a moderate reliability score. The consensus prediction was mutagenic, albeit with a low consensus score, indicating low reliability due to model disagreement and a borderline prediction probability, which suggested a higher likelihood of mutagenicity. Overall, the mutagenicity prediction demonstrates that most of the selected phlorotannins were predicted to be non-mutagenic.

#### Computational analysis of binding stability and conformational changes via MD Simulation

MD simulation was performed to evaluate the structural stability and conformational changes of the protein-ligand complex. Three protein-ligand complexes were selected based on the docking results, toxicity, and mutagenicity of the compounds, such as Pily1 with phlorofucofuroeckol B, pyocyanin biosynthetic protein PhzM with phlorofucofuroeckol B, and pyoverdine with fucodiphloroethol G. To evaluate the structural dynamics and ligand-induced stability of these complexes, analyses of RMSD, RMSF, Rg, and hydrogen bonds were conducted.

**Fig. 7:**
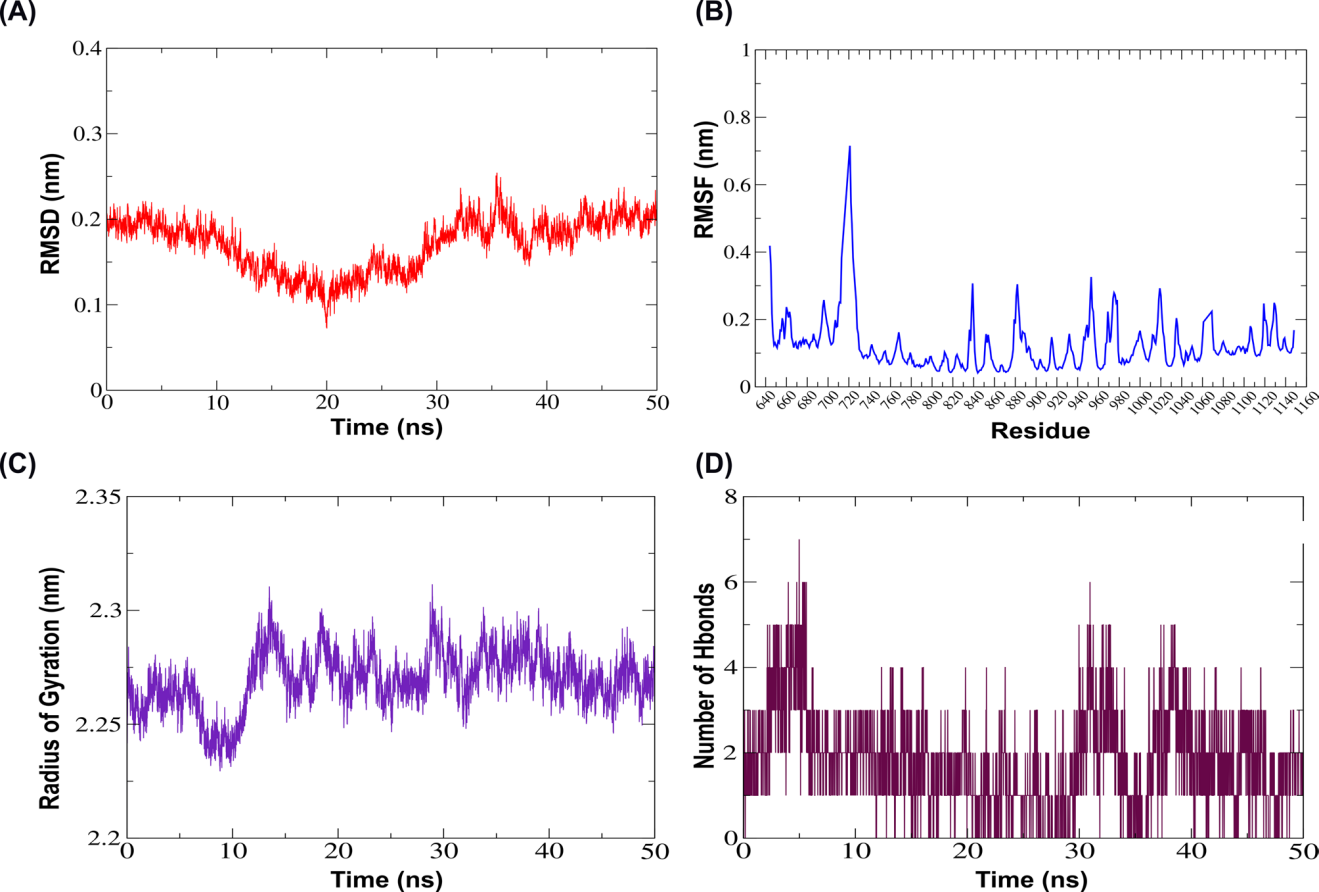
Structural stability and interaction analysis of the type IV pilus biogenesis factor PilY1-phlorofucofuroeckol-B complex during 50ns molecular dynamics simulation. **A** Root Mean Square Deviation (RMSD) of the protein backbone Cα atoms indicates structural stability over time. Initially, the RMSD decreased from 0.2 nm to around 0.1 nm during first 20ns, indicating structural relaxation of the protein-ligand complex. From 20ns onward, the RMSD stabilizes around (~ 0.2 ± 0.05) nm, suggesting a well-equilibrated and conformationally stable system throughout the simulation. **B** Root Mean Square Fluctuation (RMSF) plot of Cα showing per-residue flexibility of the protein backbone. Most of the residues exhibited limited flexibility, fluctuating within a 0.1–0.3 nm window, indicative of a structurally stable protein backbone. A notable peak between residues 705–725 reaches ~ 0.7 nm, suggesting localized flexibility or loop motion in this region, which might contribute to ligand interaction or functional dynamics. **C** Radius of Gyration (Rg) represents the compactness of the protein structure throughout the simulation. The Rg fluctuates between 2.25 and 2.28 nm during the initial 5 ns, followed by a transient dip to ~ 2.23 nm around 10 ns, suggesting a brief compacting event. A peak of ~ 2.31 nm occurs near 15 ns, after which the Rg stabilizes between 2.25–2.30 nm, indicating maintenance of the protein’s overall compactness and structural integrity throughout the simulation.**D** Hydrogen bond (H-bond) analysis depicting the number of intermolecular hydrogen bonds between the type IV pilus biogenesis factor PilY1-phlorofucofuroeckol-B.The number of hydrogen bonds initially rises from 1 to 7 within the first 5 ns, followed by a drop, and fluctuates mostly between 1 and 3 up to 30 ns. A secondary increase is observed between 30 and 40 ns time frame, reaching up to 6 bonds. Throughout the simulation, transient loss of hydrogen bonding is indicated by intermittent zero values, suggesting dynamic interaction and possible repositioning of the ligand within the binding site

In the type IV pilus biogenesis factor PilY1-phlorofucofuroeckol B complex, RMSD values initially decreased from 0.2 nm to 0.1 nm during the first 20 ns, then stabilized around ~ 0.2 ± 0.05 nm, indicating a well-equilibrated complex (Fig. 7A). RMSF values fluctuate within a 0.1–0.3 nm window, and a notable peak fluctuation was observed between residues 705–725, reaching ~ 0.7 nm, indicating regional flexibility or loop movement that might have enhanced the ligand interactions (Fig. 7B). The Rg values fluctuate during the initial 5 ns from 2.25 to 2.28 nm, followed by a transient dip to approximately 2.23 nm around 10 ns. A peak of ~ 2.31 nm occurred near 15 ns, then Rg stabilized between 2.25 and 2.30 nm, demonstrating the preservation of the protein’s compactness (Fig. 7C). Hydrogen bond dynamics, the initial H-bonds increased from 1 to 7 within the first 5 ns, followed by a drop and fluctuating between 1 and 3 H-bonds up to 30 ns. A secondary increase was observed up to 6 H-bonds from 30 ns to 40 ns time frames, indicating the possibility of dynamic reposition of the ligand within the binding pocket (Fig. 7D).

**Fig. 8:**
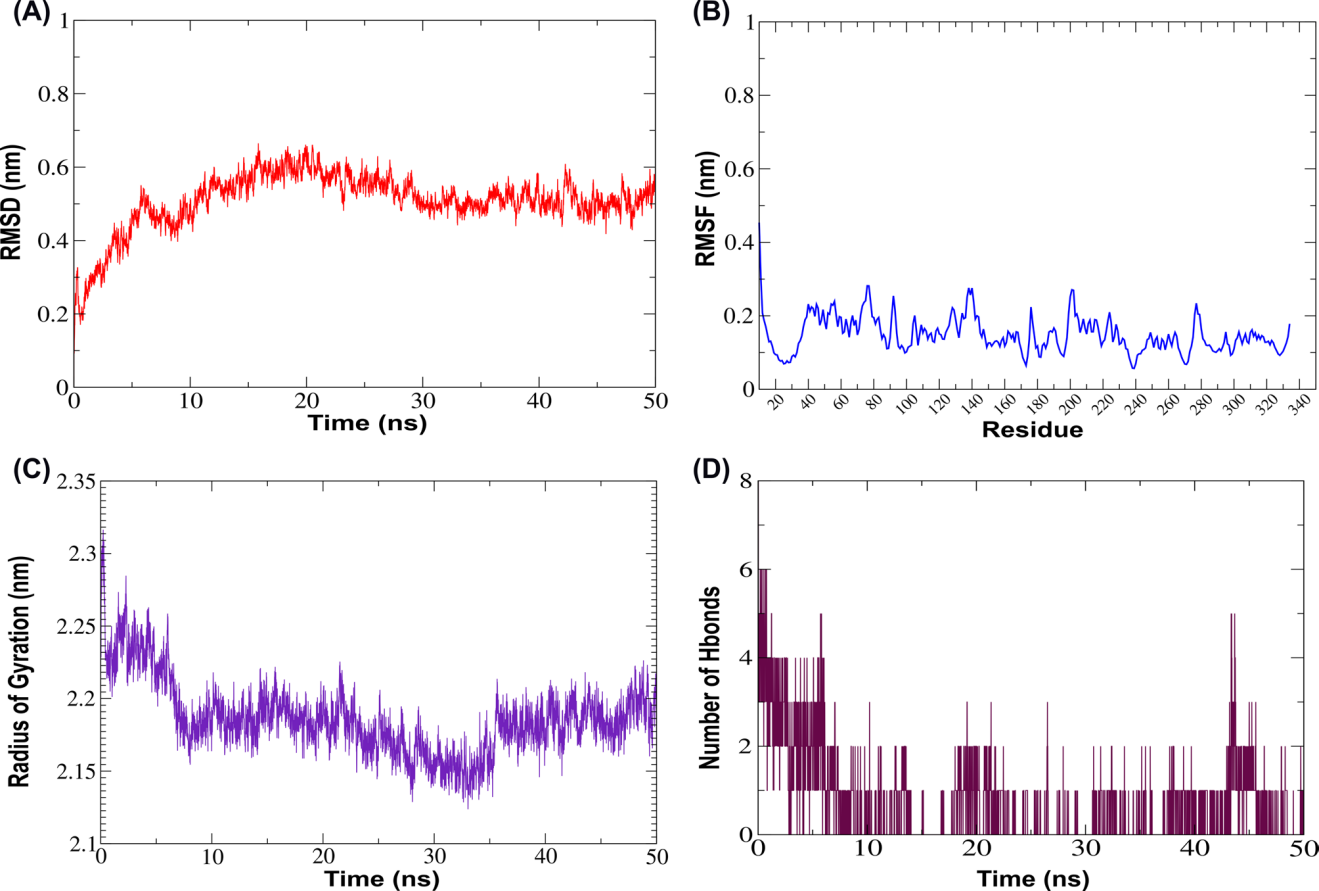
Structural stability and interaction analysis of the phenazine-1-carboxylate N-methyltransferase-phlorofucofuroeckol-B complex during 50ns molecular dynamics simulation. **A**Root Mean Square Deviation (RMSD) of the protein backbone Cα atoms indicates structural stability over time. Initially, RMSD increased from 0.2 nm to 0.6 nm during the first 20 ns. From 20 ns onward, the RMSD stabilizes around (~ 0.5 ± 0.1) nm, suggesting a structural stability. **B** Root Mean Square Fluctuation (RMSF) demonstrates the per-residue flexibility of the protein backbone. Most residues exhibited limited flexibility, fluctuating within a 0.1–0.3 nm window, indicating a structurally stable protein backbone. **C** Radius of Gyration (Rg) represents the compactness of the protein structure throughout the simulation, indicating consistent structural integrity. The Rg fluctuates between 2.2 and 2.3 nm during the initial 5 ns, followed by a transient dip up to 30 ns, then Rg values stabilize around 2.2 nm, indicating the protein’s overall compactness throughout the simulation. **D** Hydrogen bond (H-bond) analysis depicting the number of intermolecular hydrogen bonds between the phenazine-1-carboxylate N-methyltransferase-phlorofucofuroeckol-B. The number of hydrogen bonds initially rose from 1 to 6 within 5 ns, followed by a drop, and fluctuated between 0 and 3 up to 40 ns. An increase is observed between 40 and 50 ns, reaching up to 5 bonds

Phenazine-1-carboxylate N-methyltransferase-phlorofucofuroeckol-B complex, RMSD values increased from 0.2 nm to 0.6 nm during the first 20 ns, then stabilized at ~ 0.5 ± 0.1 nm, suggesting structural stability (Fig. 8A). RMSF fluctuated within a 0.1–0.3 nm window, indicating a structurally stable backbone (Fig. 8B). Rg fluctuated between 2.2 and 2.3 nm during the initial 5 ns, then it showed a transient dip up to 30 ns, reaching up to 2.15 nm and finally stabilizing after 35 ns around a Rg value of 2.2 nm, confirming the overall structural compactness (Fig. 8C). The number of hydrogen bonds initially rose from 1 to 6 within five ns, followed by a drop, and mainly fluctuated between 0 and 3 up to 40 ns. An increase in the number of H-bonds was observed between 40 and 50 ns time frames, reaching up to 5 hydrogen bonds, demonstrating possible restructuring or dynamic stabilization in ligand binding (Fig. 8D).

**Fig. 9:**
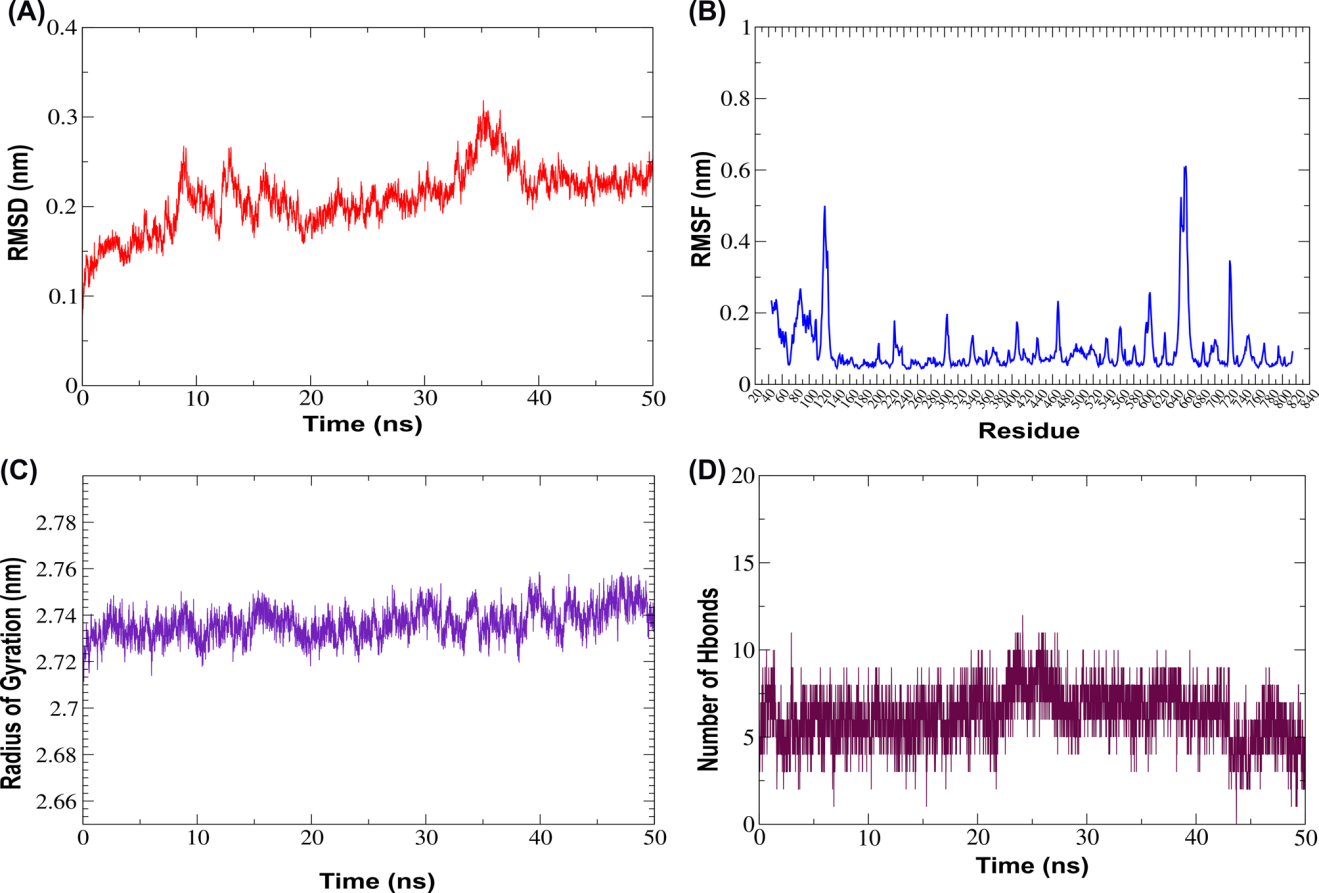
Structural stability and interaction analysis of the ferripyoverdine receptor-fucodiphloroethol-G complex during 50ns molecular dynamics simulation. **A** Root Mean Square Deviation (RMSD) of the protein backbone Cα atoms indicates structural stability over time. Initially, RMSD increased from 0.1 nm to 0.25 nm during the first 15 ns. From 15 ns to 35 ns, the RMSD stabilizes around (~ 0.2 ± 0.05) nm, and a secondary increase was observed from 0.2 nm to 0.3 nm between 35 ns and 40 ns. From 40 ns onward, the RMSD stabilized around (~ 0.23 ± 0.05) nm. **B** Root Mean Square Fluctuation (RMSF) shows the per-residue flexibility of the protein backbone. Most of the residues exhibited limited flexibility, fluctuating within a 0.1–0.25 nm window, indicative of a structurally stable protein backbone. Notable peaks were observed between residues 110–140 and 640–670, with RMSF peaks reaching up to ~ 0.5 nm and ~ 0.6 nm, respectively. **C** Radius of Gyration (Rg) represents the compactness of the protein structure throughout the simulation, indicating consistent structural integrity. The Rg values were consistent throughout the simulation, slightly fluctuating between 2.72 and 2.75 nm, confirming the protein’s overall compactness. **D** Hydrogen bond (H-bond) analysis depicting the number of intermolecular hydrogen bonds between the ferripyoverdine receptor and fucodiphloroethol-G. The number of hydrogen bonds initially rises from 3 to 12 bonds throughout the simulation, indicating strong and stable interactions

Ferripyoverdine receptor-fucodiphloroethol-G complex RMSD increased from 0.1 to 0.25 nm during the first 15 ns. From 15 ns to 35 ns, the RMSD remained constant around (~ 0.2 ± 0.05 nm), and a secondary increase was observed from 0.2 nm to 0.3 nm between 35 ns and 40 ns. From 40 ns onward, the RMSD stabilized around (~ 0.23 ± 0.05) nm, signifying an overall structurally steady state (Fig. 9A). RMSF mostly showed peaks between 0.1 and 0.25 across the length of the protein, although notable peaks were observed between residues 110–140, and 640–670, with RMSF peaks reaching upto 0.5 nm, and 0.6 nm, respectively, indicating flexible regions that might contribute to the ligand binding (Fig. 9B). Rg values were consistent throughout the simulation and fluctuated between 2.72 and 2.75 nm, indicating that the system maintained a compact and stable conformation (Fig. 9C). The number of hydrogen bonds initially rose from 3 to 12, and then remained around 7 to 8 throughout the simulation, indicating strong and stable interactions (Fig. 9D).

## Discussion

*Pseudomonas aeruginosa*, a gram-negative opportunistic pathogen, has developed resistance to antimicrobial agents, making it challenging to treat and limiting the therapeutic options. Seaweed-derived compounds have demonstrated the potential antimicrobial activity against *P. aeruginosa*, particularly through disrupting their biofilm structure (Azam and Khan 2019; Rima et al. 2022). While extensive research has focused on extracts containing phlorotannins and their antibacterial properties, studies focusing on purified phlorotannin compounds remain limited (Lemesheva et al. 2023). To address this gap, this study conducted a comprehensive *in silico* analysis of phlorotannins, employing molecular docking, pharmacokinetic profiling, mutagenicity prediction, and molecular dynamics simulation against essential virulence proteins of *P. aeruginosa*.

Furthermore, Pearson correlation analysis was conducted between the physicochemical properties of phlorotannins and MIC values reported in previous studies. Our results suggested that aromaticity, lipophilicity, molecular size, and polar surface area could be key physicochemical factors influencing the antibacterial potential of phlorotannins. These physicochemical properties enhance the drug-target binding, membrane permeability, solubility, and overall pharmacokinetics of compounds (Meanwell 2011). This finding aligns with previous research, which reported that the physicochemical profile of the compounds plays a significant role in determining biological activity, ADMET behaviour, the penetration of the drug into bacteria, and interaction with the receptor protein (Macielag 2012).

In our molecular docking study, seven phlorotannins consistently demonstrated strong binding affinities toward various virulence proteins of *P. aeruginosa*, including QS-regulating proteins, cell surface components, biofilm-associated proteins, proteolytic enzymes, iron acquisition proteins, host adhesion factors, and toxin-producing virulence factors. These complexes were stabilized through multiple non-covalent interactions, such as conventional hydrogen bonds, van der Waals forces, several π-π interactions, and salt bridges. Previous research has identified key residues within the LasR binding pocket, such as Tyr47, Thr75, Tyr93, Asp73, and Ser129, which are essential for ligand stabilization. Our study revealed that several phlorotannins, such as bifuhalol, difucol, and phloroglucinol, interacted with three or more residues. Marine natural products are involved in various interactions, such as hydrogen bonds, water bridges, hydrophobic interactions, and ionic interactions. (Singothu and Bhandari 2024). While difucol demonstrated diverse interactions, including conventional hydrogen bonds, π-donor hydrogen bonds, π-π stacking, π-π T-shaped interactions, and π-alkyl interactions. These findings are consistent with the previously described mechanism and emphasize a distinctive interaction profile for LasR targeting. In this study, MvfR-2-phloroeckol showed a higher binding affinity of -9.29 kcal/mol than MvfR-M64 complex (-9.18 kcal/mol) (Almihyawi et al. 2021). Both ligands formed hydrogen bonds, van der Waals forces, and π-π interactions. However, 2-phloroeckol displayed more diverse interactions, including amide-π stacking, π-alkyl interactions, π-sigma interactions, and carbon-hydrogen bond interactions. A prior study identified three primary residues, Ile186, Ile236, and Ile263, which improve affinity in all MvfR-ligand complexes (Vieira et al. 2022). Our results consistently showed interaction with Ile236 and Ile263. Similarly, RhlR-phlorofucofuroeckol B formed conventional hydrogen bonds, π-cation, π-sigma, and π-alkyl interactions, consistent with the interaction profile of RhlR in complex with rotiorinol, suggesting the involvement of similar interaction types in stabilization of the ligand within the active site of RhlR (Skariyachan et al. 2020). The acyl-homoserine-lactone synthase-phlorotannin molecular docking analysis shows that among the fifteen phlorotannins, eleven consistently interact with the Ile107 residue. Prior research reported that Ile107 was a common interaction residue among the highest-performing marine compounds with LasI, thereby enhancing binding affinity (Singothu et al. 2023). The phloroglucinol-Pily1 and propenyl guaethol-Pily1 complexes demonstrated significant interaction with residues in the calcium-binding domain of Pily1, which is essential for type IV pili. The phloroglucinol complex engaged with Asp891, His797, Asn890, Lys879, and Ala877, Ile876. While propenyl guaethol complex showed similar interacting residues, including Asn881, Lys879, Val880, Asn890, Ala877, Ile876, and His797. Notably, the residues Lys879, Asn890, Ala877, Ile876, and His797 were common to both complexes, highlighting the significance of ligand binding. The Phlorofucofuroeckol B complex exhibited stronger binding affinity (–10.24 kcal/mol) than the propenyl guaethol complex (–5.2 kcal/mol), suggesting our ligand formed a stable and biologically significant interaction (Srivastava et al. 2024). Likewise, the elastase-quercetin complex interacted with Trp115, Ala113, His144, Glu141, and Tyr155 residues, which are biologically significant for the receptor, contributing to substrate identification and catalytic activity. Bifuhalol also engaged with elastase via Asn163, His144, Tyr155, Glu164, Trp115, and Glu141. Among them, Trp115, His144, Glu141, and Tyr155 were consistently present in both complexes. Furthermore, 2-phloroeckol complex demonstrated a high binding affinity of − 9.939 kcal/mol compared to quercetin complex, which showed a lower binding affinity of − 7.9 kcal/mol (Ren et al. 2024).

Similarly, residues such as Asn20, Asp36, Trp41, Tyr151, and Thr117 of protease LasA were found to interact with 2-phloroeckol. These residues are located in the active site of LasA, which is responsible for staphylolytic and elastolytic action. Notably, these residues were also engaged in ligand binding with azlocillin. The Fucodiphloroethol G–LasA complex (− 9.466 kcal/mol) showed a stronger binding affinity compared to the azlocillin-LasA complex (− 8.2 kcal/mol) (Qais et al. 2024). The PA-I galactophilic lectin interacted with phloroglucinol via the residues Tyr36, Thr104, Asn107, Asp100, His50, and Gln53. These residues were biologically important for the receptor, facilitating calcium coordination, sugar recognition, and structural stabilization within the carbohydrate-binding region. Significantly, His50 and Gln53 also reported to stabilize the βGal moiety in the αGal1–3Gal disaccharide-lectin I complex, as reported by (Blanchard et al. 2008). The lectin-II-α-L-fucose complex exhibited the key residues Glu95, Asp99, Asp101, Asp104, Asn103, and Gly114 involved in the interactions. Similarly, fucose-binding lectin PA-IIL-phlorofucofuroeckol A complexes interacted via key residues of Ser22, Ser23, Thr45, Asp104, Asp101, Glu95, Asp99, Thr98, and Gly97. These residues were significantly important for the receptor due to their role in glycan identification and calcium-dependent binding. Notably, Glu95, Asp99, Asp101, and Asp104 were frequently found in both complexes (Mishra et al. 2008). Taken together, these findings underscore the multitargeting capability of phlorotannin and their ability to interact with biologically significant residues across the various virulence factors.

The pharmacokinetic profile of the compound outlines its absorption, distribution, metabolism, excretion, and toxicity properties. In the initial stage of drug development, the selected compound must be non-carcinogenic and non-hepatotoxic to ensure its suitability (Al Azzam 2022). The current findings from the ADMET analysis of phlorotannins indicated that most phlorotannins possess favourable pharmacokinetic parameters. Specifically, phloroglucinol and isoliolide demonstrated excellent water solubility, Caco-2 permeability, and plasma availability. These phlorotannins also did not interact with P-glycoproteins and cytochrome P450 enzymes. All phlorotannins were predicted not to be substrates of renal OCT2, and the majority of the phlorotannins were non-toxic, lacked hepatotoxicity, and had a tolerable level of oral toxicity. These findings were in close agreement with a previous study, which reported that among the phlorotannins tested, phloroglucinol exhibited high water solubility and high Caco-2 permeability and did not interact with P-glycoprotein, which functions as an efflux pump. This indicated that phloroglucinol may have better bioavailability (Renagupita, 2023). Cytochrome P450 metabolizes most of the medications, and it is essential to predict whether a drug interacts with any of these enzymes or another drug, as this can result in drug-drug interactions. The phlorotannins phloroglucinol, phlorofucofuroeckol-A, eckol, dioxinodehydroeckol, 7-phloroeckol, dieckol, and 6,6′-bieckol were predicted not to be substrates of CYP3A4. The rate of excretion and elimination of the drug from the body determines the interval between doses, and all phlorotannins except 6,6′-bieckol were predicted not to be substrates of renal OCT2, suggesting a lower risk of drug-drug interactions (Croft et al. 2012; Renagupita, 2023). However, dioxinodehydroeckol was predicted to be AMES positive, indicating the compound is mutagenic and may act as a mutagen (Chakraborty et al. 2024).

VEGA (Q) SAR is a platform used to analyze the mutagenicity of chemical compounds, aiding the screening and selection of suitable compounds. This platform utilizes various models to evaluate their mutagenicity, toxicity, and carcinogenic potency (Nikunj et al. 2020). This study used VEGA (Q) SAR to predict the mutagenicity of the selected phlorotannins. Most of the phlorotannins were predicted as non-mutagenic, including 2-phloroeckol, 7-phloroeckol, difucol, diphlorethohydroxycarmalol, eckol, diphlorethol, fucodiphloroethol G, fucophlorethol A, isololiolide, phlorofucofuroeckol A, phlorofucofuroeckol B, phloroglucinol, and triphloroethol A. In contrast, bifuhalol and dioxinodehydroeckol were predicted as mutagenic. These computational results align with the previous Ames study, demonstrating that the eckol did not exhibit mutagenic activity (Nabiya et al. 2022). However, studies focused on the computational investigation of phlorotannins’ mutagenicity were limited. The experimental analysis of the early study reported that phlorotannin extracts from *Fucus serratus* and *Ectocarpus siliculosus* seaweeds have a slight mutagenic effect at higher concentrations in certain bacterial strains(Tarakhovskaya et al. 2023). This underscores the importance of compound-level evaluation and supports the reliability of the computational approach in early toxicity screening.

The molecular dynamics (MD) simulation results support the structural stability along with the binding properties of phlorotannin-virulence complexes. For the three selected complexes, the RMSD values remained around 0.25 nm throughout the 50 ns simulation period for Pily1-Phlorofucofuroeckol-B and Pyoverdine-Fucodiphloroethol-G, and ~ 0.6 nm for the Pyocyanin-Phlorofucofuroeckol-B complex, indicating well-equilibrated systems and nearly stable protein-ligand interactions under physiological conditions (Srivastava et al. 2024). The RMSF values of these complexes fluctuate within a 0.1–0.3 nm window, highlighting conformational stability. These findings align with the prior investigation of 3FXI-oxathiapiprolin complex, which exhibited reduced fluctuation in the binding site, indicating a more rigid and stable interaction at the active binding site (Yadav et al. 2024). The radius of gyration for these three complexes was relatively constant throughout the simulation period, with only minor fluctuations indicating structural compactness and stability. This observation was consistent with MCC950-NLR P3 complexes; Rg values remained stable during the simulation. It suggests the complex did not induce significant changes, highlighting its stable nature and supporting previous studies on protein-ligand interactions (Igor et al. 2021). The hydrogen bond analysis showed that the three complexes formed a high number of hydrogen bonds, particularly the ferripyoverdine receptor-fucodiphloroethol G complex, which formed up to 12 hydrogen bonds. The 3CL protease-nirmatrelvir complex formed multiple hydrogen bonds, indicating that this strong and stable interaction may contribute to its high binding affinity and inhibitory potential (Wang et al. 2024). These selected complexes exhibited consistent structural stability across the 50 ns simulation period, indicated by RMSD, RMSF, radius of gyration, and hydrogen bond profiles. The result suggests that the essential dynamic behaviour of the protein-ligand complex was effectively captured within this time frame. Previous studies on *P. aeruginosa* virulence factors have also employed 50 ns simulations to obtain insight into molecular interactions. Therefore, the 50 ns simulation was appropriate for assessing the binding and stability of phlorotannin complexes (Sadiq et al. 2020). Overall, *in silico* investigations demonstrated that pure phlorotannins were potentially non-toxic, non-mutagenic, and exhibited high binding affinities, better pharmacokinetic properties, and structural stability when interacting with key virulence proteins of *P. aeruginosa*. These results suggested that these compounds have the potential to inhibit virulence proteins, highlighting their significance in the development of novel antimicrobial drugs.

## Conclusion and future perspectives

The comprehensive *in silico* investigation demonstrated that brown algal phlorotannins may serve as multi-target antivirulence agents against *Pseudomonas aeruginosa*. This research indicated that many phlorotannins, including 2-phloroeckol, 7-phloroeckol, difucol, diphlorethohydroxycarmalol, fucodiphloroethol G, phlorofucofuroeckol A, and phlorofucofuroeckol B, have strong and stable interactions with essential virulence proteins.

These phlorotannins effectively interact with QS-regulating proteins, cell surface components, biofilm-associated proteins, proteolytic enzymes, iron acquisition proteins, host adhesion, and toxin-producing proteins, thereby attenuating bacterial virulence. This was achieved through the integration of molecular docking, dynamic simulation, pharmacokinetic profiling, and mutagenicity assessment. Furthermore, favourable ADMET and mutagenicity profiles validate their potential as safe lead compounds for further preclinical assessment. Overall, these findings support further exploration of phlorotannin-based antivirulence therapeutics, particularly in combating antibiotic-resistant *P. aeruginosa* infections. Utilizing marine-derived materials may offer an innovative and sustainable method in antimicrobial research.

While these results establish a solid computational foundation for developing phlorotannin-based antivirulence therapeutics, further research is required to confirm their efficacy and safety through experimental studies.

- *In vitro* and *in vivo* investigations are essential to confirm the anticipated binding affinities and virulence mitigation of phlorotannins in *P. aeruginosa*.
- Comprehensive SAR investigations are required to elucidate the significant chemical features of phlorotannins that inhibit virulence proteins and enhance lead compounds.
- Investigate the potential synergistic effects of phlorotannins in conjunction with sub-minimum inhibitory concentrations to enhance efficacy and mitigate antibiotic resistance.
- Incorporate additional steps into the computational and experimental methodologies to evaluate the efficacy of phlorotannin against significant pathogens, known as SKAPE pathogens.
- Conduct comprehensive toxicological studies, including chronic exposure, immune system, cytotoxicity, and animal models.

## Supplementary Information

Below is the link to the electronic supplementary material.

Supplementary Material 1

## Data Availability

No datasets were generated or analysed during the current study.

## Abbreviations

MD simulation: Molecular dynamic simulation
ADMET: Absorption, Distribution, Metabolism, Excretion, Toxicity
MDR: Multi-drug resistant
TPSA: Topological polar surface area
MW: Molecular weight
RMSD: Root mean square deviation
RMSF: Root mean square fluctuation
Rg: Radius of gyration
MIC: Minimum inhibitory concentration
CS: Consensus score
GADI: Global applicability domain index
MTD: Maximum tolerated dose
QS: Quorum sensing
PDB: Protein data bank
pkCSM: Pharmacokinetics computational structure modelling
SPCE: Simple point charge extended
P-gp: P-glycoprotein
CYP: Cytochrome P
