# Supplementary material for: Marine-derived phlorotannins: sustainable inhibitors of multiple virulence factors in Pseudomonas aeruginosa
Source: AMB Express. 2025 Oct 31;15:162. doi: 10.1186/s13568-025-01963-w (PMC12579053; doi:10.1186/s13568-025-01963-w)
Supplement: Supplementary file 1 — Supplementary Material 1 [file 13568_2025_1963_MOESM1_ESM.pdf]

Table S1. This table provides information on selected virulence factors/proteins of *Pseudomonas aeruginosa*, including their UniProt accession numbers, alternative names, associated PDB IDs, and functional descriptions. All proteins listed have been functionally validated with evidence at the protein level, as reported in UniProt.

Uniprot ID	Name of virulence factor	Alternative names of the virulence factor	PDB ids	Functional description	References
Q03023	Serralysin	Alkaline metalloproteinase (AP)	1AKL	Preferential cleavage of bonds with hydrophobic residues in P1	(Morihara et al. 1973)
P14756	Elastase	Neutral metalloproteinase<br>PAE<br>Pseudolysin	1EZM	Cleaves host elastin, collagen, IgG, and several complement components, as well as endogenous pro-aminopeptidase.	(Braun et al. 1998; Cahan et al. 2001; McIver et al. 1991; Olson and Ohman 1992)
P42512	Fe (3+)-pyochelin receptor	Fe (III)-pyochelin receptor	1XKW	High-affinity outer membrane receptor required for the transport of Fe3+-pyochelin.	(Cobessi et al. 2005)
P25084	Transcriptional activator protein LasR	-	2UV0	Transcriptional activator of the elastase structural gene (LasB). Binds to the PAI autoinducer.	(Bottomley et al. 2007)
P48632	Ferripyoverdine receptor	-	2W78	Receptor for the siderophore ferripyoverdine	(Greenwald et al. 2009)
S0HPF7	Type IV pilus biogenesis factor PilY1	Pilus-associated adhesin PilY	3HX6	Involved in pilus assembly, twitching motility, and adhesion to host cells. Prime type IV pili (T4P) assembly is required for inclusion of minor pilins PilV, PilW, and PilX to the surface pili. Calcium-binding and calcium release by PilY1 seem to be essential for	(Heiniger et al. 2010; Johnson et al. 2011; Orans et al.

				twitching motility and for the regulation of pilus retraction dynamics of PilT.<br>Adhesin for human tissue specifically recognizing a host receptor localized or enriched on basolateral epithelial cell surfaces. Binds host integrins in a calcium-dependent manner in vitro, and this interaction may be employed by the bacterium to mediate host epithelial cell binding in vivo.	2010)
P14789	Protease LasA	Staphylolytic protease	3IT5	Involved in proteolysis and elastolysis (degradation of the host protein elastin). Has staphylolytic activity (degrades pentaglycine cross-links in cell wall peptidoglycan.<br>Degradation of host elastin is likely to contribute to the pathogenicity of P. aeruginosa. intermediate.	(Spencer et al. 2010; Vessillier et al. 2001)
O86266	Flagellin	-	4NX9	Flagellin is the subunit protein that polymerizes to form the filaments of bacterial flagella.	(Song and Yoon 2014)
Q9HWH2	Phenazine-1-carboxylate N-methyltransferase	-	2IP2	Involved in the biosynthesis of pyocyanine, a blue-pigmented phenazine derivative, which plays a role in virulence. Converts phenazine-1-carboxylate (PCA) to 5-methylphenazine-1-carboxylate (5-methyl-PCA).	(Parsons et al. 2007)
P33883	Acyl-homoserine-lactone synthase	Autoinducer synthesis protein LasI	1RO5	Required for the synthesis of PAI, consisting of 3-oxo-N-(tetrahydro-2-oxo-3-furanyl)-dodecanamide also known as N-(3-oxododecanoyl) homoserine lactone, an autoinducer molecule which binds to LasR and thus acts in elastase biosynthesis regulation.	(Gould et al. 2004)
Q51372	Alginate biosynthesis protein AlgX	Probable alginate O-acetyltransferase AlgX	4KNC	Plays two roles in the biosynthesis of the exopolysaccharide alginate: protects alginate from degradation as the polymer traverses the periplasm, and also plays a role in its O-acetylation. Acetylation of alginate causes the cells in the biofilm to adhere better to lung epithelium, form microcolonies, and resist the effects of the	(Riley et al. 2013; Robles-Price et al. 2004)

				host immune system and/or antibiotics.	
Q9HZE4	PelA	-	5TCB	PelA plays an important role in biofilm by hydrolyz-ingthe pel exopolysaccharide.	(Snarr et al. 2017)
Q9I4X0	Multiple virulence factor regulator MvfR	Transcriptional regula-tor MvfR	6B8A,	Transcription regulator that plays a critical role in viru-lence by positively regulating the expression of multi-ple quorum sensing (QS)-regulated virulence factors, genes involved in protein secretion, translation, re-sponse to oxidative stress, and the phnAB operon	(Cao et al. 2001; Maura et al. 2016; Xiao et al. 2006)
Q9I4X2	2-heptyl-4(1H)-quinolone synthase subunit PqsB	-	6ESZ	Required for the biosynthesis of the quorum-sensing signaling molecules 2-heptyl-4(1H)-quinolone (HHQ) and 2-heptyl-3-hydroxy-4(1H)-quinolone (Pseudomo-nas quinolone signal or PQS), which are important for biofilm formation and virulence.	(Drees et al. 2016; Dulcey et al. 2013)
Q9I6M7	Type VI secretion system spike protein VgrG2b	Metallopeptidase ef-fector VgrG2b	6H56	Part of the H2 type VI secretion system (H2-T6SS) specialized secretion system, which delivers several virulence factors in both prokaryotic and eukaryotic cells during infection.<br>The spike at the tip of the elongating tube is probably formed by haemolysin co-regulated protein 2b/Hcp2b (Probable). Allows the delivery of the Tle3 antibacte-rial toxin to target cells where it exerts its toxicity.	(Berni et al. 2019; Sana et al. 2015; Wood et al. 2019)
P54292	HTH-type quorum-sensing regulator RhlR	Regulatory protein RhlR<br><br>Elastase modulator	8DQ0	Quorum-sensing regulator that controls the expression of multiple virulence factors in response to extracellu-lar signaling molecules called autoinducers.<br>Involved, among others, in the transcriptional regula-tion of genes responsible for rhamnolipid surfactant biosynthesis. In addition, it is involved in regulating the production of elastase (lasB) and pyocyanine.	(Brint and Ohman 1995; Latifi et al. 1995; Me-dina et al. 2003; Ochsner et al. 1994; Ochsner

					and Reiser 1995; Wagner et al. 2004)
Q05097	PA-I galactophilic lectin	Galactose-binding lectin	1OKO	D-galactose-specific lectin.<br>It helps the bacteria to adhere to the host tissues, especially in cystic fibrosis patients.	(Cioci et al. 2003)
Q9HYN5	Fucose-binding lectin PA-IIL	-	1UZV	PA-IIL is involved in the bacterial colonization and adhesion in host tissues.	(Mitchell et al. 2005)

Table S2. This table includes phlorotannin compounds, selected as ligands for docking, along with previous studies that demonstrate their activity against various virulence factors/proteins across diverse bacterial species. It also lists various physicochemical parameters predicted for all phlorotannin compounds used in this study, using MolGpKa (Graph-Convolutional Neural Network) for pKa prediction and Swiss-ADME for LogP, TPSA, MW, H-bond Acceptors and Donors, Rotatable bonds, and Aromaticity predictions.

**Previous study**			**This study**							
**Phlorotannin**	**MIC(µg/mL): Bacterial Strain**	**References**	**LogP**	**TPSA (A2)**	**MW (g/mol)**	**H-bond Acceptors**	**H-bond Donors**	**Rotatable bonds**	**Lowest pKa**	**Aromaticity**
2-Phloroeckol	NA	-	4.814	198.76	496.38	12	8	4	7.5	4
7-Phloroeckol	128:(*P.acnes, S.epidermidis, L.monocytogenes*) 256:(*P.acnes, S.aureus*) 64:(*L.monocytogenes*)	[1, 2]	4.814	198.76	496.064	12	8	4	7.1	4
Bifuhalol	NA	-	1.713	130.61	266.043	6	7	2	8.2	2
Difucol	NA	-	1.587	121.38	250.048	6	6	1	7.3	2
Dioxinodehydroeckol	64:(*P.acnes, S.epidermidis, L.monocytogenes*) 128:(*P.acnes, S.aureus, L.monocytogenes*)	[1, 2]	4.324	153.71	370.032	9	5	0	8	5
Diphlorethohydroxycarmalol	128:(*S.aureus, P.aeruginosa*)	[3]	4.52	218.99	512.059	13	9	4	6.7	4
Diphlorethol	NA	-	2.007	110.38	250.048	6	5	2	8.3	2
Eckol	128:(*S.epidermidis, L.monocytogenes*) 256:(*P.acnes, S.aureus, L.monocytogenes*) 250:(*S.aureus, S.gallinarum, S.typhimurium*)	[1, 2, 4]	3.611	149.07	372.048	4	6	2	7.9	3

Fucodiphloroethol G	NA	-	3.994	220.76	498.08	12	10	5	6.9	4
Fucophlorethol A	NA	-	2.791	171.07	374.064	9	8	3	6.9	3
Isololiolide	NA	-	1.409	46.53	196.11	3	1	0	13.8	0
Phlorofucofuroeckol A	128:(*S.epidermidis*) 256:(*P.acnes*, *S.aureus*) 32:(*Methicillin-resistant S.aureus*)	[1, 5]	6.419	232.13	602.07	14	9	4	7.2	6
Phlorofucofuroeckol B	NA	-	6.419	232.13	602.07	14	9	4	7.1	6
Phloroglucinol	2048:(*P.aeruginosa*)	[6]	0.803	60.69	126.032	3	3	0	9.2	1
Triphloroethol A	NA	-	3.21	160.07	374.064	9	7	4	8.1	3

NA: Not available

Table S3. Detailed docking result of Quorum sensing regulatory proteins of *Pseudomonas aeruginosa* with phlorotaninns. The table presents the minimum binding free energies of the complexes and the aminoacid residues involved in the interaction. All the residues are shown in standard 3-letter code along with the aminoacid position within the peptide chain.

Virulence protein	Phlorotannins	Binding Free Energy (kcal/mol)	Key residues involved in interaction
Transcriptional activator protein LasR	2-Phloroeckol	-8.36	PHE87, SER82, HIS78, GLN94, SER91, GLU89
	7-Phloroeckol	-8.182	SER91, ILE86, GLN98, ILE92
	Bifuhalol	-8.951	THR75, SER129, LEU36, ASP73, TYR64, ALA127, VAL76, TYR47, ALA50
	Difucol	-9.865	ALA105, TRP88, TYR93, LEU110, THR115, THR75, ASP73, TYR56, LEU36, TYR64
	Dioxinodehydroeckol	-8.125	CYS79, GLY38, THR75, THR115, PHE101, TRP88, LEU110, TYR56, ALA105, ASP73, TRP60, ARG61, LEU36, TYR64, VAL76, ALA127
	Diphlorethohydroxycarmalol	-8.246	ALA2, ILE22, GLU9, LYS25, ALA27, ASP29, LEU30, SER146, VAL147, THR150
	Diphlorethol	-9.801	TRP60, TYR64, ARG61, ALA127, LEU36, ASP73, SER129, THR75, THR115
	Eckol	-7.072	HIS78, ILE86, GLN98, ILE92, SER77, GLN81
	Fucodiphloroethol G	-7.515	SER77, HIS78, GLN81, SER82
	Fucophlorethol A	-9.4	THR75, SER129, ALA127, GLY38, ALA50, LEU39, VAL76, LEU36, TYR64, TRP60, TYR56, LEU110, TRP88
	Isololiolide	-7.455	GLY126, TYR47, ALA50, ALA127, LEU125, CYS79, VAL76, LEU40
	Phlorofucofuroeckol A	-8.116	ILE86, GLN4, PRO85, PHE87, GLU145, GLN81, HIS78, SER77
	Phlorofucofuroeckol B	-7.88	GLN94, GLN81, SER77, PHE87
	Phloroglucinol	-6.296	THR75, THR115, SER129, ASP73, TRP60, LEU110,

			ALA105, TYR93
	Triphloroethol A	-7.972	THR75, THR115, PHE101, TRP88, TYR56, LEU110, ALA105, TRP60, ARG61, LEU36, TYR64, GLY38, ALA127, VAL76, CYS79
Acyl-homoserine-lactone synthase	2-Phloroeckol	-8.232	ARG172, GLY147, THR145, PHE105, ARG30, GLU171
	7-Phloroeckol	-7.828	PHE105, ALA106, ILE107, GLU171, ARG172, ILE170, GLY147, THR145
	Bifuhalol	-6.049	THR121, THR145, GLU171, ILE107, PHE105, VAL148
	Difucol	-6.638	SER109, ILE107, ARG30, VAL148, THR145, THR144
	Dioxinodehydroeckol	-7.319	THR144, THR145, VAL148, ARG30, SER109, ILE107, VAL26
	Diphlorethohydroxycarmalol	-7.623	VAL146, GLY147, ARG172, THR145, ILE107, VAL26, GLN25, ARG30, GLU171
	Diphlorethol	-6.778	SER109, ARG30, VAL26, THR144
	Eckol	-7.428	GLN25, SER109, ASN108, VAL26, ILE107, ARG30
	Fucodiphloroethol G	-8.368	VAL26, SER109, ILE107, ARG30, PHE105, PHE27, THR144, THR145, GLU171
	Fucophlorethol A	-7.922	ILE107, PHE117, VAL26, GLN25
	Isololiolide	-6.328	ILE107, PHE117, VAL148, THR145, THR144, VAL26, ARG30
	Phlorofucofuroeckol A	-7.727	THR145, VAL146, SER118, ARG154, GLY147, ARG172
	Phlorofucofuroeckol B	-8.638	ARG30, VAL148, SER118, MET151, GLU171
	Phloroglucinol	-4.211	ILE107, ARG30, VAL26
	Triphloroethol A	-7.359	THR144, THR145, VAL148, ARG30, SER109, ILE107, VAL26
Multiple virulence factor regulator MvfR	2-Phloroeckol	-9.292	LEU207, LEU197, LEU208, ILE236, ALA168, LYS167, ILE149, PRO238, ALA102, ASP264, THR265
	7-Phloroeckol	-8.788	ILE149, THR265, LEU207, ILE236, PRO238, ALA168, ALA102

	Bifuhalol	-7.488	LEU197, ARG209, LEU208, ILE236, THR265, ALA168, ALA102, ILE149
	Difucol	-7.981	LEU207, THR265, PRO238, ALA168, ALA102, ILE149, LEU208, ILE236
	Dioxinodehydroeckol	-8.308	LEU197, SER196, GLN194, LEU208, LEU207, ILE263, THR265, ALA168, ILE236
	Diphlorethohydroxycarmalol	-7.621	LEU251, ALA252, LEU178, CYS179, ALA181, LEU183, VAL253
	Diphlorethol	-7.84	THR265, GLN194, SER196, LEU208, ILE236
	Eckol	-7.969	LEU197, LEU208, ILE236, LEU207, ALA168, THR265
	Fucodiphloroethol G	-7.787	LEU246, VAL253, ALA181, CYS179, PRO174, ALA252
	Fucophlorethol A	-8.403	LEU197, LEU208, ILE236, ALA168, LEU207, ASP264, ILE263, TYR258
	Isololiolide	-6.416	ALA102, PRO238, PHE221, ILE149, LEU207, ALA168, ILE236
	Phlorofucofuroeckol A	-8.741	ARG209, TYR258, GLU259, ASP264, ILE263, THR265, LEU208, GLN194, LEU207, ILE236
	Phlorofucofuroeckol B	-8.443	GLU151, THR265, LEU207, ILE236, LEU197, LEU208, TYR258
	Phloroglucinol	-4.898	TYR258, LEU183, VAL170, LEU189, LEU254
	Triphloroethol A	-7.634	GLN194, ARG209, LEU197, LEU208, ILE236, LEU207
2-heptyl-4(1H)-quinolone synthase subunit PqsB	2-Phloroeckol	-9.202	ALA238, PHE274, LEU229, PRO271
	7-Phloroeckol	-9.282	VAL241, TRP333, ALA238, ALA329, HIS269
	Bifuhalol	-7.05	THR171, ARG168, LEU172, PHE173
	Difucol	-7.424	PHE173, ARG168, THR171, LEU172, LEU229
	Dioxinodehydroeckol	-8.765	ARG168, THR171, LEU172, PHE173, PRO271, LEU229
	Diphlorethohydroxycarmalol	-10.2	LEU275, VAL241, HIS269, TRP333, ALA238, MET237
	Diphlorethol	-8.112	THR331, TYR204, GLY209, GLU128
	Eckol	-8.633	LEU275, PHE274, PHE173, LEU172, THR171, ARG168
	Fucodiphloroethol G	-10.16	LEU229, GLN234, SER231, PHE230, LEU172, THR171,

			ARG168, PHE173, TRP39
	Fucophlorethol A	-9.221	LEU172, PHE173, THR171, ARG168, TRP333, PRO271, HIS269
	Isololiolide	-6.398	TRP35, LEU172, PHE173, LEU229, TRP39
	Phlorofucofuroeckol A	-9.012	ILE301, THR331, SER85, PRO271
	Phlorofucofuroeckol B	-9.627	PRO271, PHE38, SER34, SER32, MET237
	Phloroglucinol	-4.994	THR171, ARG168, LEU172, PHE173
	Triphloroethol A	-8.69	THR169, ARG168, THR171, LEU172, PHE173, HIS269, PRO271
HTH-type quorum-sensing regulator RhlR	2-Phloroeckol	-7.185	LEU174, ASN128, ARG186, LEU240, LEU181, VAL179, SER176, MET175
	7-Phloroeckol	-7.409	SER176, LEU174, ASN128, MET175, ARG186, LEU181
	Bifuhalol	-5.363	PHE53, THR54, LEU230, THR229, ALA223
	Difucol	-5.492	GLY239, ASN128, SER176, LEU174, MET175
	Dioxinodehydroeckol	-6.366	LEU181, VAL179, ASN177, PRO178, SER176, THR167, LEU174
	Diphlorethohydroxycarmalol	-7.585	THR54, ARG55, PRO52, SER176, MET175, ASN177, LYS57, HIS49, ILE51
	Diphlorethol	-5.61	THR229, LEU230, PHE53, THR54
	Eckol	-6.297	PHE53, PRO56, ALA236, ALA237, ASN129, ARG125, THR50
	Fucodiphloroethol G	-7.201	ARG55, PRO52, TYR234, PHE53, LEU230, ASP224, ASN177
	Fucophlorethol A	-7.229	LEU174, MET175, SER176, LEU181, VAL179, LEU240, ARG186
	Isololiolide	-5.015	LEU230, THR54, PHE53, ALA233
	Phlorofucofuroeckol A	-7.202	THR192, ALA232, ALA193, ALA236, LEU88, ARG125, ARG48, THR50, ALA233
	Phlorofucofuroeckol B	-7.752	LYS228, THR229, ALA232, SER198
	Phloroglucinol	-4.28	ARG55, THR58, ASN76, ALA79

	Triphloroethol A	-6.377	LEU181, VAL179, PRO178, ASN177, SER176, THR167, LEU174

Table S4. Detailed docking result of cell surface components and biofilm-associated proteins of *Pseudomonas aeruginosa* with phlorotaninns. The table presents the minimum binding free energies of the complexes and the aminoacid residues involved in the interaction. All the residues are shown in standard 3-letter code along with the aminoacid position within the peptide chain.

Virulence protein	Phlorotannins	Binding Free Energy (kcal/mol)	Key residues involved in interaction
Type IV pilus biogenesis factor PilY1	2-Phloroeckol	-9.637	LEU927, ASP851, LEU657, GLY856, VAL793, TYR653, ARG848
	7-Phloroeckol	-9.746	GLY856, ALA858, VAL793, VAL734, LYS790, PRO733, VAL735, PRO645, ARG848, THR792, LEU849, ALA794
	Bifuhalol	-6.551	SER732, VAL734, THR792, ASP1045, ARG848, VAL735
	Difucol	-6.581	TYR653, ILE661, ALA858, ALA794
	Dioxinodehydroeckol	-8.086	VAL734, VAL735, PRO645, GLN652, TYR653, ALA794, ARG848, THR792, LYS790
	Diphlorethohydroxycarmalol	-9.772	PRO733, LEU849, GLY856, ALA794, ALA858, TYR653, ARG848, ASP1045
	Diphlorethol	-6.748	GLN652, VAL734, VAL735, LYS790, THR792
	Eckol	-7.966	PRO645, ALA646, VAL735, GLN652, THR792, ASP1045, LYS790, VAL734
	Fucodiphloroethol G	-9.522	ALA794, ALA858, GLY856, LEU849, TYR936, LEU927, ARG848
	Fucophlorethol A	-8.577	LEU849, LEU927, ALA850, ASP851, TYR936, ARG929, TYR1079
	Isololiolide	-5.786	ALA794, ALA858, LEU849, TYR653
	Phlorofucofuroeckol A	-9.973	ILE661, TYR653, ALA794, GLY856, LEU849, THR792, PRO791, LYS790, LEU1123, THR1124, ARG848
	Phlorofucofuroeckol B	-10.24	VAL793, TYR653, GLY856, LEU657, THR792,

			LYS790, ASP1045, VAL734
	Phloroglucinol	-5.28	ASP891, HIS797, ASN890, ILE876, LYS879, ALA877
	Triphloroethol A	-8.125	LYS790, VAL734, VAL735, PRO645, GLN652, TYR653, ALA794, ARG848, THR792
Flagellin	2-Phloroeckol	-8.942	GLN82, GLY79, GLN83, ARG124, THR128, THR129, ALA80, THR130, THR76, GLN75
	7-Phloroeckol	-7.742	THR128, ALA80, THR76, GLN75, GLN321, GLN82, GLY79, GLN83, ARG124
	Bifuhalol	-5.746	SER167, ASN169, ALA312, ASP161, ASP316, GLN313
	Difucol	-6.157	ALA80, ILE125, THR129, ARG124, THR128
	Dioxinodehydroeckol	-6.84	GLN75, THR76, THR128, THR129, ARG124, ALA80, GLN83, GLY79
	Diphlorethohydroxycarmalol	-7.671	ALA80, GLY79, GLN82, THR129, THR128
	Diphlorethol	-6.041	THR130, THR128, ILE125, ARG124, GLN83
	Eckol	-6.896	GLN83, GLY79, GLN75, THR76, THR128, THR129, ALA80
	Fucodiphloroethol G	-7.217	THR129, THR128, THR130, GLY132, PHE131, THR76
	Fucophlorethol A	-7.761	GLY79, THR76, ILE125, ALA80, THR128, ARG124
	Isololiolide	-5.459	PHE131, PHE146, ALA144
	Phlorofucofuroeckol A	-7.795	GLN313, GLY159, ASP316, ASP161, ALA312, GLN309, ASN169, GLY170, THR171, GLU305, LYS308
	Phlorofucofuroeckol B	-7.693	GLY79, ALA80, THR129, THR128, THR76, SER72
	Phloroglucinol	-4.441	THR171, LEU301, LYS246, GLU305, LYS308, ALA247, ASP304
	Triphloroethol A	-6.966	THR128, ARG124, THR129, ILE125, ALA80, THR130, THR76
Alginate biosynthesis protein AlgX	2-Phloroeckol	-9.177	LYS223, LEU218, LYS56, TYR57, PHE60, GLY296, HIS175, GLY298, THR268, HIS176, LYS217

	7-Phloroeckol	-8.218	PHE60, TYR328, HIS327, SER269, HIS175, SER216, LYS217, LYS223
	Bifuhalol	-7.651	SER269, ASN270, HIS175, GLN112, TYR170, ARG74, ALA325, HIS327
	Difucol	-7.014	ALA168, LYS172, TRP71, VAL238, HIS167, ASP239
	Dioxinodehydroeckol	-7.297	HIS327, HIS175, PHE60, SER269, GLY296, THR268, GLY298, TYR328, PHE299, GLY297, ASP300, GLN332
	Diphlorethohydroxycarmalol	-9	THR76, GLY59, THR62, TYR328, GLY84, ASP329, SER86, THR85, TYR77, ARG80
	Diphlorethol	-8.143	HIS327, ARG74, TYR170, ALA325, HIS175, ASN270, THR268, SER269
	Eckol	-7.791	GLY296, SER269, THR268, ASP300, PHE299, GLN332, HIS327, TYR328, HIS175, PHE60
	Fucodiphloroethol G	-9.192	THR220, TYR57, SER269, PHE60, THR268, GLY296, HIS175
	Fucophlorethol A	-7.72	TYR328, PHE60, SER269, HIS175
	Isololiolide	-5.855	ALA168, THR178, HIS180, VAL238, HIS167
	Phlorofucofuroeckol A	-9.051	TYR77, HIS327, LYS56, PHE60, PHE299, TYR328, ASP300, ASP329
	Phlorofucofuroeckol B	-9.102	ARG80, THR76, TYR77, GLY298, PHE299, GLN332, TYR328
	Phloroglucinol	-6.101	HIS327, ARG74, HIS175, TYR170, ALA325, GLN112, THR326
	Triphloroethol A	-8.808	HIS327, ARG74, TYR170, ALA325, HIS175, ASN270, SER269, GLY296
PelA	2-Phloroeckol	-9.515	VAL234, ASP238, GLU233, PRO235, ARG232, SER219, TRP224, PRO289, GLU218, TYR261
	7-Phloroeckol	-9.012	GLU101, SER98, TYR96, GLU218, VAL234, PRO235, ARG200, ALA226
	Bifuhalol	-6.497	PRO235, ASP238, ARG200, ASP163, GLU218,

			TRP224, SER219, VAL234
	Difucol	-6.839	ASP238, ASP237, TRP241, GLU203
	Dioxinodehydroeckol	-7.718	ASP163, ASN199, SER219, GLU233, ARG232, PRO235, ASP238, VAL234
	Diphlorethohydroxycarmalol	-9.253	ASP160, ASN199, ALA226, ASP225, PRO235, ARG232, GLU218, SER164, GLU101
	Diphlorethol	-6.486	SER219, GLY223, TRP224, ASP238
	Eckol	-8.089	ASP163, GLU218, ASN199, SER219, GLU233, ARG232, PRO235, ASP238, ASP225, VAL234, ALA226
	Fucodiphloroethol G	-8.701	GLU218, SER219, VAL234, ASP238, PRO235, ARG232, ASP225, ALA227, ALA226
	Fucophlorethol A	-8.262	TRP224, GLU218, GLY223, SER219, ASP238, ARG200
	Isololiolide	-5.866	ARG239, LYS243, ARG250, HIS221, TYR282
	Phlorofucofuroeckol A	-8.99	ARG200, ASP163, GLU218, ASP160, ASP103, TRP126, SER128, GLU101, SER219, TRP224, ASP238, VAL234
	Phlorofucofuroeckol B	-9.251	ASP225, GLU218, TRP224, TYR261, SER219, GLY223
	Phloroglucinol	-4.489	ARG232, ASP225, TRP224, PRO235, ASP238
	Triphloroethol A	-7.713	ASN199, GLU218, SER219, GLU233, ARG232, PRO235, ASP238, VAL234, ASP163

Table S5. Detailed docking result of proteolytic enzymes and iron acquisition proteins of *Pseudomonas aeruginosa* with phlorotaninns. The table presents the minimum binding free energies of the complexes and the aminoacid residues involved in the interaction. All the residues are shown in standard 3-letter code along with the aminoacid position within the peptide chain.

**Virulence protein**	**Phlorotannins**	**Binding Free Energy (kcal/mol)**	**Key residues involved in interaction**
Protease LasA	2-Phloroeckol	-9.042	TYR151, HIS122, ASP36, ASN20, THR117, TRP41
	7-Phloroeckol	-9.238	ASP36, HIS120, HIS122, ASN25, GLU112, GLN66, GLY114
	Bifuhalol	-7.424	GLY114, TYR80, ASN25, HIS122, HIS120, ASP36, TYR151
	Difucol	-7.093	HIS122, SER115
	Dioxinodehydroeckol	-8.247	GLN66, HIS120, HIS81, TYR15, SER115, ASN25
	Diphlorethohydroxycarmalol	-8.163	GLU112, GLY114, SER115, HIS81, HIS122, ASN25, GLN66
	Diphlorethol	-7.504	HIS120, HIS81, HIS122, ASN25
	Eckol	-8.167	GLY114, SER115, ASP36, HIS120, TYR151, HIS122, ASN25, TYR80
	Fucodiphloroethol G	-9.466	GLY27, THR26, TYR151, ASN25, SER115, HIS122, HIS120, ASP36
	Fucophlorethol A	-9.147	GLN66, ASN25, SER115, HIS122, HIS81
	Isololiolide	-5.561	HIS120, THR117, SER115, TYR151, ASN20
	Phlorofucofuroeckol A	-8.413	TYR80, PHE131, GLU112, GLY113, ASN25, ARG68, ASN78, GLN66
	Phlorofucofuroeckol B	-8.756	GLU112, GLN66, TYR80, HIS81, HIS122, HIS23, ASP36, TYR151, SER115
	Phloroglucinol	-4.94	ASN25, HIS122, TYR80, GLN66
	Triphloroethol A	-8.255	GLN66, HIS81, HIS122, HIS120, TYR151, SER115, ASN25
Serralysin	2-Phloroeckol	-9.557	GLU266, ARG267, GLN101, PHE111, SER104,

			HIS110, THR251, ASN249, ARG253
	7-Phloroeckol	-9.156	HIS110, PHE111, ARG267, ASN264, GLU266, ARG253, THR254, THR252, ASP105, ASN249
	Bifuhalol	-7.23	VAL32, ARG18, ALA15, TYR16, PHE12, LEU13, ASN304, ALA309, LYS308
	Difucol	-7.251	ASN304, ARG18, LYS308, ALA309, PHE12
	Dioxinodehydroeckol	-8.006	ARG267, ARG253, THR252, THR251, ASN249, ASP105, PHE111, HIS110, ILE109, SER104,
	Diphlorethohydroxycarmalol	-9.357	HIS110, ARG267, GLU266, THR265, ARG253, THR252, ASP105
	Diphlorethol	-6.753	THR251, ASN249, HIS110, PHE111, SER104,
	Eckol	-8.076	HIS110, VAL54, ASN249, SER104, GLN101
	Fucodiphloroethol G	-9.296	PHE111, ILE109, HIS110, VAL54, THR251, ASN249, THR252, ASP105, SER104
	Fucophlorethol A	-8.412	TYR59, HIS110, ASN108, ASN249, THR251
	Isololiolide	-5.546	LEU310, LYS308, ARG18
	Phlorofucofuroeckol A	-9.765	THR252, ARG267, ASP105, GLU266, ARG253, ASP256, ASN264, GLN101, PHE111
	Phlorofucofuroeckol B	-9.746	SER53, VAL54, HIS110, SER104, TYR59, GLN101
	Phloroglucinol	-4.686	MET214, TYR216, HIS186, ASP207, GLY188, TRP217, ASP189
	Triphloroethol A	-8.074	ARG267, ARG253, THR252, ASN249, THR251, ASP105, PHE111, HIS110, SER104
Elastase	2-Phloroeckol	-9.939	ARG198, GLU164, HIS223, TYR155, HIS144, ALA113, GLU141,
	7-Phloroeckol	-9.068	TRP115, GLY152, LEU153, TYR155, ASN163, HIS144, GLU141
	Bifuhalol	-6.867	ASN163, HIS144, TYR155, GLU164, TRP115, GLU141
	Difucol	-6.676	GLY152, ASN163, TYR155, HIS144

	Dioxinodehydroeckol	-7.705	TRP115, ASP116, ASN163, HIS144, TYR155, GLU164, GLU141
	Diphlorethohydroxycarmalol	-8.816	ILE154, TYR155, GLU141, ALA113, HIS144, ASN163
	Diphlorethol	-7.205	ASN163, TYR155
	Eckol	-7.53	ASN163, TYR155, HIS144, GLU164, GLU141, TRP115
	Fucodiphloroethol G	-8.545	GLU111, ARG198, HIS140, LEU197, ASP206, HIS224
	Fucophlorethol A	-8.806	TYR155, HIS144, ASN163, TRP115
	Isololiolide	-6.007	ASN218, TYR216, GLN158, SER159, THR262, SER263, ARG156, ILE154
	Phlorofucofuroeckol A	-9.007	TRP115, TYR155, GLU164, ASN112, HIS144, ASN163, GLU148, GLY152
	Phlorofucofuroeckol B	-9.735	TYR155, ALA113, TYR114, TRP115, GLU141, HIS144, ASN163, LEU153, ARG156
	Phloroglucinol	-4.067	HIS144, ASN163, TYR155
	Triphloroethol A	-7.676	TRP115, ASP116, ASN163, HIS144, TYR155, GLU164, GLU141
Fe3+-pyochelin receptor	2-Phloroeckol	-11.45	GLU708, GLN695, ARG705, TYR232, PRO113, ASP100, ASN686
	7-Phloroeckol	-12.24	GLN104, GLN105, GLN645, PRO147, THR269, THR119, TYR232, GLU708, TYR693
	Bifuhalol	-8.122	GLN104, ASN686, SER711, GLU708, ASN713, ASN95, ARG200, GLU101, GLN105
	Difucol	-8.066	GLU708, TYR693, ASN686, GLN645, GLN105, GLN104
	Dioxinodehydroeckol	-9.701	ASN686, ASP100, PRO113, TYR706, GLU708, TYR693
	Diphlorethohydroxycarmalol	-11.67	GLN237, PRO147, PHE114, TYR230, ARG228, GLU101, GLN645, GLN105, GLU708, GLN104, ASP100

	Diphlorethol	-8.937	GLN104, ASN686, SER711, GLU708, ASN95, ARG200, GLU101, GLN105
	Eckol	-9.678	ASN686, ASP100, TYR706, PRO113, GLU708, TYR693
	Fucodiphloroethol G	-11.05	PHE231, PRO113, ARG705, TYR232, PHE114, LEU117, THR118, THR119, PRO147, ASP100, GLN237, GLU708, GLN104
	Fucophlorethol A	-10.84	TYR232, ASP236, THR269, ILE268, TYR230, PRO113, GLY707, GLU708, GLN695
	Isololiolide	-7.287	TYR230, GLN237, SER267, PHE114, PRO147, TRP300
	Phlorofucofuroeckol A	-10.95	GLN94, ASN686, GLU101, TYR693, ARG228, THR118, LEU117, THR119, PRO147, GLU708
	Phlorofucofuroeckol B	-8.966	ASP227, ARG240, ASP236, ASP229, ASP233, PHE231, TYR230, ARG297, ASP266
	Phloroglucinol	-5.897	ARG717, LEU91, LEU96, MET150, TYR153, GLU204, THR192
	Triphloroethol A	-9.638	GLN104, GLN237, TYR230, TYR232, TYR693, GLN695, GLU708
Ferripyoverdine receptor	2-Phloroeckol	-9.291	SER320, TRP288, SER287, HIS319, LYS318, ASN290, ASP803, ASP187, ARG190, ALA199
	7-Phloroeckol	-8.644	ASP323, ARG380, SER379, SER381, TRP288, HIS319, ASP289
	Bifuhalol	-8.645	ASP764, ARG190, ARG735, VAL197, LEU762, TYR789
	Difucol	-8.521	ARG327, MET322, ALA199, SER800, SER365, TYR801, ARG190, ASP186
	Dioxinodehydroeckol	-11.03	TYR789, ASN783, ASP803, ASN806, ASN290, GLN316, LYS318, ASP187, PHE180, GLY181, ASN782, ASP764, LEU762
	Diphlorethohydroxycarmalol	-9.49	ARG327, LYS318, ASN783, ASP764, TYR716,

			LEU762, ARG190, ASP187, ASN290, ARG292, GLN316
	Diphlorethol	-8.59	TYR789, VAL197, ARG735, THR196, LEU762, ARG190, TYR716, HIS191
	Eckol	-10.91	TYR789, ASN783, ASP803, ASN806, GLN316, ASN290, LYS318, ASP187, GLY181, PHE180, ASN782, ASP764, LEU762
	Fucodiphloroethol G	-11.6	ASN782, HIS191, ASP187, ASN184, THR202, ARG190, ARG327, LYS318, SER320, ASP803, LEU807, SER287, ASN290, MET808
	Fucophlorethol A	-9.938	ASN782, ASN783, ASP764, ARG190, TYR789, LYS318, ASN183, GLN316, ARG292
	Isololiolide	-6.821	ARG190, MET322, ALA199
	Phlorofucofuroeckol A	-9.068	ARG190, MET808, SER810, ASN780, ASN183, GLN316, ASP803
	Phlorofucofuroeckol B	-8.314	PHE321, SER381, TRP288, HIS324, HIS319
	Phloroglucinol	-5.402	LYS720, ARG768, SER718, GLY684
	Triphloroethol A	-10.95	TYR789, ASN783, ASP803, ASN806, ASN290, GLN316, LYS318, ASP187, PHE180, GLY181, ASN782, ASP764, LEU762

Table S6. Detailed docking result of host adhesion and toxin-producing proteins of *Pseudomonas aeruginosa* with phlorotaninns. The table presents the minimum binding free energies of the complexes and the aminoacid residues involved in the interaction. All the residues are shown in standard 3-letter code along with the aminoacid position within the peptide chain.

Virulence protein	Phlorotannins	Binding Free Energy (kcal/mol)	Key residues involved in interaction
PA-I galactophilic lectin	2-Phloroeckol	-6.807	THR104, ASN107, ASP100, TYR36, PRO38, GLN53, HIS50
	7-Phloroeckol	-7.053	TRP42, ARG48, GLN45, GLY43, GLN40
	Bifuhalol	-5.936	PRO38, GLY37, ASN107, ASP100, THR104, GLN53, HIS50, TYR36
	Difucol	-5.496	LYS41, ARG48, GLN45, TRP42
	Dioxinodehydroeckol	-6.731	ASN107, THR104, GLN53, HIS50, TYR36, PRO38, GLY37
	Diphlorethohydroxycarmalol	-8.011	THR104, HIS50, TYR36, PRO38, GLY37, ASN107
	Diphlorethol	-6.142	ASN107, THR104, PRO38, TYR36, HIS50
	Eckol	-6.771	GLN53, TYR36, HIS50, PRO38, GLY37, ASN107, THR104
	Fucodiphloroethol G	-7.291	ASN107, THR104, ASP100, TYR36, PRO38
	Fucophlorethol A	6.844	THR74, LYS68, ILE56, GLU11
	Isololiolide	-4.829	CYS62, ASP52, PHE61
	Phlorofucofuroeckol A	-7.44	ASN78, HIS50, ALA60, HIS58, ASP52, PHE61
	Phlorofucofuroeckol B	-7.372	GLY43, TRP42, TRP33, LYS41, GLN40
	Phloroglucinol	-4.223	TYR36, THR104, ASN107, ASP100, HIS50, GLN53
	Triphloroethol A	-6.743	ASP100, GLN53, HIS50, TYR36, PRO38, GLY37, ASN107, THR104
Fucose-binding lectin PA-IIL	2-Phloroeckol	-7.503	SER23, ASN103, SER22, GLU95, ASP99, ASP104, ASP96, THR98
	7-Phloroeckol	-7.908	PRO73, LEU76, ASP75, GLN3, SER74, VAL67,

			GLN66
	Bifuhalol	-6.132	ASP101, ASP99, ASP104, GLU95, ASP96, GLY97, SER22, SER23, THR45
	Difucol	-5.706	SER74, LEU76, VAL67, GLN66, GLN64, THR2
	Dioxinodehydroeckol	-6.968	SER23, ASP96, SER22, ASN70, GLY97, THR98, ASP99, ASP104, ASP101
	Diphlorethohydroxycarmalol	-7.596	ASP101, ASP99, SER22, SER23, ASN70, GLY97, THR98
	Diphlorethol	-6.195	ASN29, SER68, VAL67, SER74, PRO73
	Eckol	-6.636	GLU95, ASP104, ASP96, ASP99, SER22, ASN103, THR45, SER23
	Fucodiphloroethol G	-7.304	GLY93, PHE89, LEU91, VAL108, ASN47, PHE19, VAL106, VAL92
	Fucophlorethol A	-7.213	GLY24, SER22, SER23, ASP99, THR98, GLY97, ASN70
	Isololiolide	-4.99	GLY93, VAL106, LEU91
	Phlorofucofuroeckol A	-8.658	SER22, SER23, THR45, ASP101, ASP104, GLU95, ASP99, GLY97, THR98
	Phlorofucofuroeckol B	-8.527	ASN29, VAL67, GLN66, GLN3, THR2, PRO73
	Phloroglucinol	-4.193	ASP75, LEU76, GLN3
	Triphloroethol A	-7.086	ASN70, GLY97, THR98, ASP99, ASP104, ASP101, SER23, ASP96, SER22
Type VI secretion system spike protein VgrG2b	2-Phloroeckol	-8.589	LEU950, ALA848, ALA954, MET904, GLN957, ALA958, ALA844
	7-Phloroeckol	-8.768	ALA847, GLN957, PRO906, MET904, VAL949, TYR902, ALA954, ALA844
	Bifuhalol	-7.456	VAL949, ALA905, HIS939, GLU936, PRO906, VAL903, MET904, TYR902, GLN957, ALA954
	Difucol	-7.399	ALA954, TYR902, HIS939, GLU936, ALA905, LEU950

	Dioxinodehydroeckol	-8.907	ALA844, ALA958, ALA954, GLN957, ALA905, HIS939, PRO906, MET904
	Diphlorethohydroxycarmalol	-8.451	TYR902, ASP901, LEU932, ARG990, ASP987, HIS935, GLU983, GLN984, GLN966, ASN981
	Diphlorethol	-7.862	GLY953, ALA905, MET904
	Eckol	-8.494	GLU983, HIS939, ALA844, ALA847, ALA954, LEU950, MET904, ALA905
	Fucodiphloroethol G	-8.975	GLN957, TYR902, ALA905, MET904, PRO906, ALA848, ALA954
	Fucophlorethol A	-9.708	VAL903, TYR902, ALA954, ALA848, ALA844, LEU950, MET904, PRO906, HIS939, GLU936
	Isololiolide	-6.478	HIS939, MET904, PRO906, ALA954, LEU950, VAL949
	Phlorofucofuroeckol A	-8.874	GLN956, TYR955, LEU951, VAL952, GLN960, ASN981, GLN966, GLN984, LYS975
	Phlorofucofuroeckol B	-9.779	GLN966, ARG970, GLU972, TYR980, LYS975, GLN956, TYR955, ARG959, GLN960, ASP965
	Phloroglucinol	-4.888	ALA905, ALA954, LEU950, VAL949, PRO906, MET904, TYR902, GLN957
	Triphloroethol A	-8.966	ALA905, HIS939, PRO906, MET904, ALA954, ALA844, ALA958, GLN957
Phenazine-1-carboxylate N-methyltransferase	2-Phloroeckol	-9.161	ARG298, GLY244, ASP245, GLY177, ARG241, GLU272, LEU291
	7-Phloroeckol	-9.491	ARG199, ASP198, GLY177, ARG241, SER178, GLU180, LEU181, GLY176, GLY175, ASP245
	Bifuhalol	-7.35	TYR102, HIS290, ARG273, GLU272, HIS299, ARG241, LEU291, TYR133
	Difucol	-7.615	HIS299, ARG241, GLU272, LEU291, HIS290, ARG273
	Dioxinodehydroeckol	-8.223	MET150, PHE157, ARG241, TYR133, GLY177, GLY176, SER178, GLY179, GLU180

	Diphlorethohydroxycarmalol	-9.426	LEU147, SER202, GLY224, MET226, ARG199
	Diphlorethol	-7.819	TYR102, ARG273, LEU291, TYR133
	Eckol	-8.273	MET150, ARG241, TYR133, SER178, GLY176, GLY179, GLU180
	Fucodiphloroethol G	-9.8	LEU291, MET150, ARG241, TYR133, SER178, GLY176, GLY179, HIS299
	Fucophlorethol A	-8.621	TYR133, LYS137, LEU136, LEU147, SER178, GLY179, GLY176, GLU180, MET150, ARG241
	Isololiolide	-6.365	ARG273, LEU291, ARG241, SER153, MET150, TRP287
	Phlorofucofuroeckol A	-9.825	SER240, ARG241, SER202, VAL205, LEU136, PRO140, LYS137, GLU180, LEU181, GLY175, ASP173
	Phlorofucofuroeckol B	-11.27	GLY176, GLY179, GLU180, SER178, GLY143, GLY201, GLY204, VAL205, LEU147, TYR133, MET150, ARG241, SER240
	Phloroglucinol	-5.066	SER240, PHE157, LEU181, GLY176, GLU180
	Triphloroethol A	-8.224	MET150, PHE157, ARG241, TYR133, GLY177, GLY176, SER178, GLY179

Table S7. Comprehensive pharmacokinetic (ADME) analysis of the phlorotannin compounds.

	Parameter	Phlorotannins														
		**1**	**2**	**3**	**4**	**5**	**6**	**7**	**8**	**9**	**10**	**11**	**12**	**13**	**14**	**15**
**Absorption**	Water solubility (log mol/L)	-2.892	-2.892	-3.006	-2.919	-3.008	-2.892	-3.265	-2.987	-2.892	-2.892	-1.401	-2.892	-2.892	-1.067	-2.923
	Caco2 permeability (log Papp in 10-6 cm/s)	0.499	0.542	-0.377	-0.774	-0.176	-0.131	0.302	0.574	-0.507	-0.529	1.217	0.542	0.503	1.094	0.337
	Intestinal absorption (human) (% Absorbed)	94.402	73.049	72.503	57.858	100	75.707	74.067	87.719	61.586	56.087	95.545	84.624	76.319	72.389	78.306
	P-glycoprotein substrate	Yes	Yes	Yes	Yes	Yes	Yes	Yes	Yes	Yes	Yes	No	Yes	Yes	No	Yes
	P-glycoprotein I inhibitor	Yes	Yes	No	No	Yes	Yes	No	No	Yes	No	No	No	No	No	No
	P-glycoprotein II inhibitor	Yes	Yes	No	No	Yes	Yes	No	Yes	Yes	Yes	No	Yes	Yes	No	Yes
**Distribution**	VDss (log L/kg)	-0.23	-0.112	0.378	0.183	-1.263	-0.071	0.162	-0.483	-0.05	0.112	-0.019	-0.068	-0.07	-0.233	-0.232
	Fraction unbound (human) (Fu)	0.384	0.383	0.135	0.205	0.139	0.382	0.143	0.312	0.382	0.332	0.611	0.385	0.385	0.553	0.319
	CYP2D6 substrate	No	No	No	No	No	No	No	No	No	No	No	No	No	No	No
	CYP3A4 substrate	No	No	No	No	No	No	No	No	No	No	No	No	No	No	No
	CYP1A2 inhibitor	No	No	Yes	Yes	Yes	No	No	No	No	No	No	No	No	No	Yes
	CYP2C19 inhibitor	No	No	No	No	Yes	No	No	No	No	No	No	No	No	No	No

**Metabolism**	CYP2C9 inhibitor	No	No	No	No	No	No	No	Yes	No	Yes	No	No	No	No	Yes
	CYP2D6 inhibitor	No	No	No	No	No	No	No	No	No	No	No	No	No	No	No
	CYP3A4 inhibitor	No	No	No	No	Yes	No	No	No	No	No	No	No	No	No	No
**Excretion**	Total Clearance (log ml/min/kg)	0.563	0.616	0.602	0.638	0.576	0.433	0.664	0.521	0.656	0.667	1.042	0.527	0.517	0.633	0.674
	Renal OCT2 substrate	No	No	No	No	No	No	No	No	No	No	No	No	No	No	No

(1) 2-phloroeckol, (2) 7-phloroeckol, (3) Bifuhalol, (4) Difucol, (5) Dioxinodehydroeckol, (6) Diphlorethohydroxycarmalol, (7) Di-phlorethol, (8) Eckol, (9) Fucodiphloroethol G, (10) Fucophlorethol A, (11) Isololiolide, (12) Phlorofucofuroeckol A, (13) Phlorofucofuroeckol B, (14) Phloroglucinol, (15) Triphloroethol A.

Table S8. Toxicity assessment of the phlorotannins by using pkCSM-pharmacokinetics

	Phlorotannins*														
**Parameter**	**1**	**2**	**3**	**4**	**5**	**6**	**7**	**8**	**9**	**10**	**11**	**12**	**13**	**14**	**15**
AMES toxicity	No	No	Yes	Yes	Yes	No	Yes	No	No	No	No	No	No	No	No
Max tolerated dose (log mg/kg/day)	0.412	0.351	1.02	0.478	0.454	0.434	0.901	0.417	0.432	0.383	0.833	0.437	0.437	0.456	0.403
hERG I inhibitor	No	No	No	No	No	No	No	No	No	No	No	No	No	No	No
hERG II inhibitor	Yes	Yes	No	No	Yes	Yes	No	Yes	Yes	Yes	No	Yes	Yes	No	Yes
Oral Rat Acute Toxicity (LD50) (mol/kg)	2.479	2.477	2.022	2.305	2.306	2.484	1.89	2.499	2.482	2.555	2.171	2.482	2.482	1.891	2.535
Oral Rat Chronic Toxicity (LOAEL) (log mg/kg_bw/day)	3.623	5.141	4.174	3.362	2.356	5.22	3.381	3.048	5.263	4.561	2.269	5.379	5.482	2.241	3.502
Hepatotoxicity	No	No	No	No	No	No	No	No	No	No	No	No	No	No	No
Skin Sensitisation	No	No	No	No	No	No	No	No	No	No	Yes	No	No	No	No

*(1) 2-phloroeckol, (2) 7-phloroeckol, (3) Bifuhalol, (4) Difucol, (5) Dioxinodehydroeckol, (6) Diphlorethohydroxycarmalol, (7) Diphlorethol, (8) Eckol, (9) Fucodiphloroethol G, (10) Fucophlorethol A, (11) Isololiolide, (12) Phlorofucofuroeckol A, (13) Phlorofucofuroeckol B, (14) Phloroglucinol, (15) Triphloroethol A.

Table S9. The mutagenicity predictions obtained from various computational Ames test models. The predictions indicate whether a compound is classified as mutagenic or non-mutagenic according to the respective model's criteria.

MODELS	CONSENSUS		CAESAR		SarPy-IRFMN		ISS		KNN-Read-Across	
**Ligands**	**Prediction**	**Reliability (CS)***	**Prediction**	**Reliability (GADI)***	**Prediction**	**Reliability (GADI)***	**Prediction**	**Reliability (GADI)***	**Prediction**	**Reliability (GADI)***
**2-phloroeckol**	Non-Mutagenic	0.4	Non-Mutagenic	0.6	Possible NON-Mutagenic	0.6	Non-Mutagenic	0.721	Non-Mutagenic	0.813
**7-phloroeckol**	Non-Mutagenic	0.4	Non-Mutagenic	0.6	Possible NON-Mutagenic	0.6	Non-Mutagenic	0.721	Non-Mutagenic	0.813
**Bifuhalol**	Mutagenic	0.45	Mutagenic	0.92	Possible NON-Mutagenic	0	Non-Mutagenic	0.743	Mutagenic	0.917
**Difucol**	Non-Mutagenic	0.35	Mutagenic	0.694	Non-Mutagenic	0.828	Non-Mutagenic	0	Non-Mutagenic	0.772
**Dioxinodehydroeckol**	Mutagenic	0.25	Suspect Mutagenic	0	Possible NON-Mutagenic	0	Mutagenic	0.624	Mutagenic	0.888
**Diphlorethohydroxycarmalol**	Non-Mutagenic	0.4	Non-Mutagenic	0.597	Possible NON-Mutagenic	0.597	Non-Mutagenic	0.719	Non-Mutagenic	0.869
**Diphlorethol**	Non-Mutagenic	0.25	Non-Mutagenic	0	Possible NON-Mutagenic	0	Non-Mutagenic	0.755	Mutagenic	0.924
**Eckol**	Non-Mutagenic	0.25	Non-Mutagenic	0	Possible NON-Mutagenic	0	Non-Mutagenic	0.731	Mutagenic	0.776
**Fucodiphloroethol G**	Non-Mutagenic	0.3	Non-Mutagenic	0.674	Non-Mutagenic	0	Non-Mutagenic	0	Non-Mutagenic	0.893
**Fucophlorethol A**	Non-	0.5	Non-	0.682	Non-	0.682	Non-	0.734	Non-	0.76

	Mutagenic		Mutagenic		Mutagenic		Mutagenic		Mutagenic	
**Isololiolide**	Non-Mutagenic	0.35	Mutagenic	0.825	Non-Mutagenic	0.698	Non-Mutagenic	0.625	Non-Mutagenic	0.857
**Phlorofucofuroeckol A**	Non-Mutagenic	0.2	Suspect Mutagenic	0.493	Non-Mutagenic	0.59	Mutagenic	0.508	Non-Mutagenic	0.801
**Phlorofucofuroeckol B**	Non-Mutagenic	0.2	Suspect Mutagenic	0.493	Non-Mutagenic	0.59	Mutagenic	0.508	Non-Mutagenic	0.801
**Phloroglucinol**	Non-Mutagenic	1	Non-Mutagenic	1	Non-Mutagenic	1	Non-Mutagenic	0.966	Non-Mutagenic	1
**Triphloroethol A**	Non-Mutagenic	0.25	Non-Mutagenic	0	Possible NON-Mutagenic	0	Non-Mutagenic	0.735	Mutagenic	0.89

*GADI: Global Applicability Domain Index

*CS: Consensus Score

Global AD index/ Consensus score <0.7: low reliability, ≥ 0.7 and <0.9: moderate reliability, ≥ 0.9 and ≤ 1: high reliability.

Supplementary figures

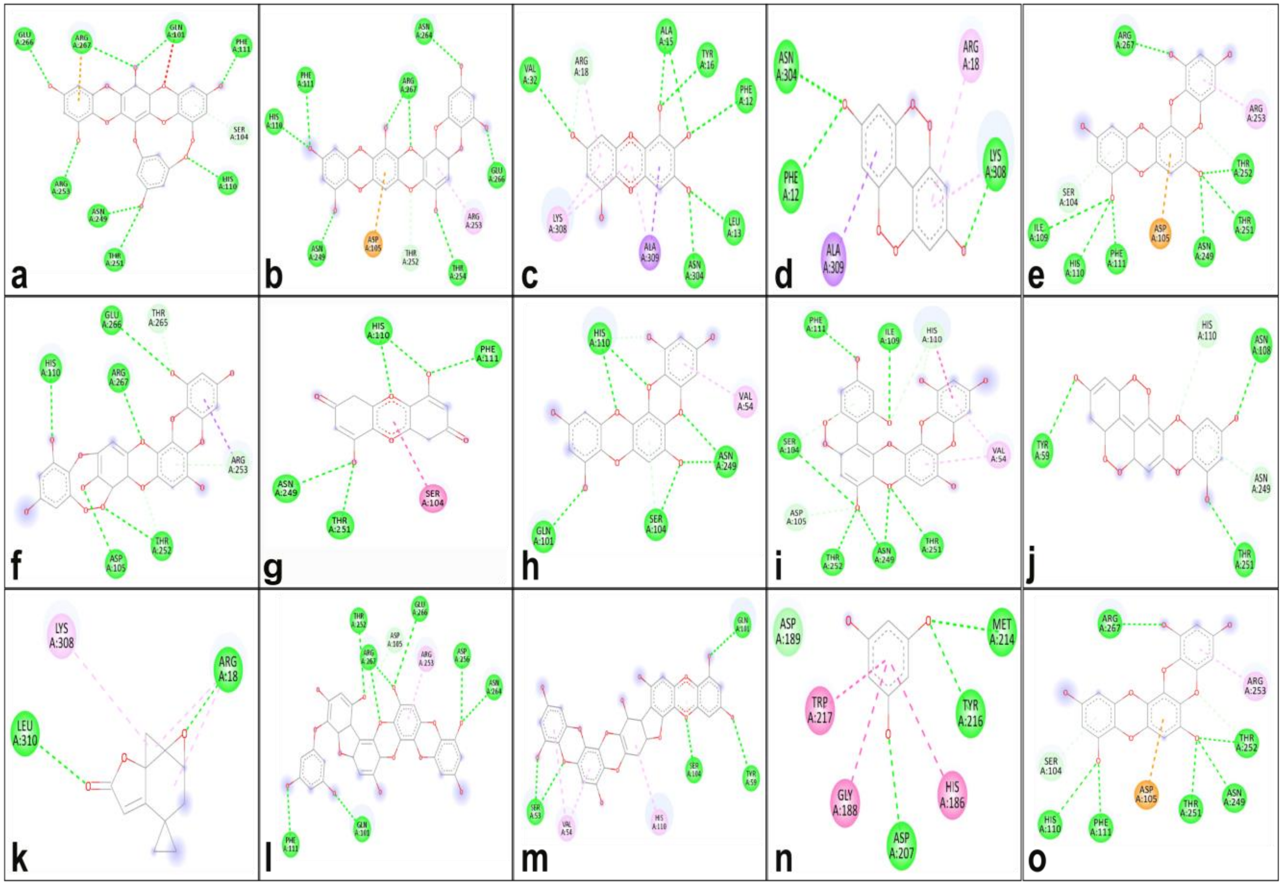

Figure S1. 2D interaction plots of serralysin protein in complex with **(a)** 2-Phloroeckol **(b)**7-Phloroeckol **(c)** Bifuhalol **(d)** Difucol **(e)** Dioxinodehydroeckol **(f)** Diphlorethohydroxycarmalol **(g)** Diphlorethol **(h)** Eckol **(i)** Fucodiphloroethol-G **(j)** Fucophlorethol-A **(k)** Isololiolide **(l)** Phlorofucofuroeckol-A **(m)** Phlorofucofuroeckol-B **(n)** Phloroglucinol **(o)** Triphloroethol-A showing the resi-

dues involved in the interaction along with the interaction types. The 3-letter code of each amino acid is shown along with the residue position and the color of dashed line and the spherical shapes (green: H-bond, orange: Cation/anion charges and salt bridges, pink: π-alkyl, purple: π-π interaction) illustrate the type of interaction.

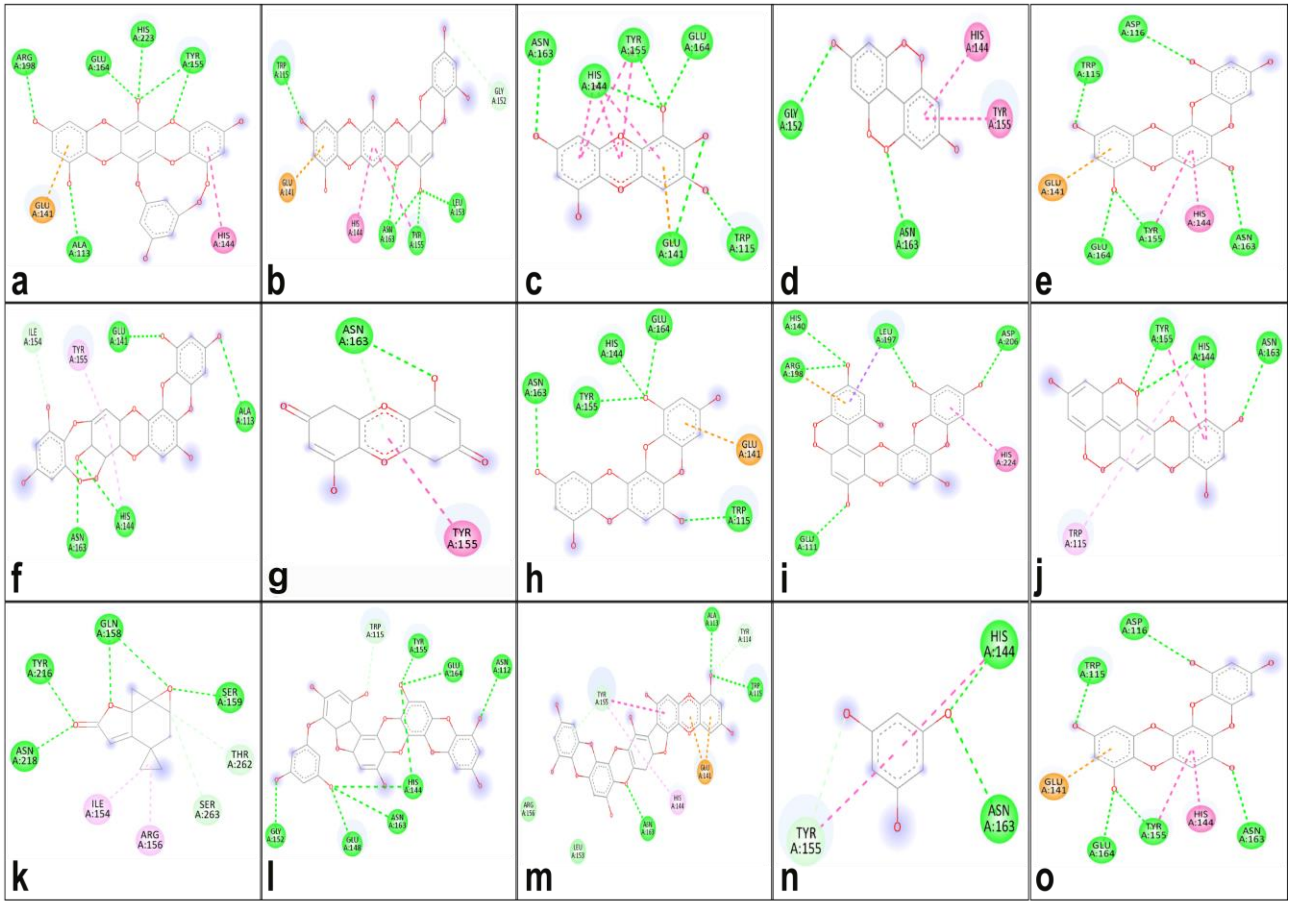

Figure S2. 2D interaction plots of elastase protein in complex with **(a)** 2-Phloroeckol **(b)**7-Phloroeckol **(c)** Bifuhalol **(d)** Difucol **(e)** Dioxinodehydroeckol **(f)** Diphlorethohydroxycarmalol **(g)** Diphlorethol **(h)** Eckol **(i)** Fucodiphloroethol-G **(j)** Fucophlorethol-A **(k)** Isololiolide **(l)** Phlorofucofuroeckol-A **(m)** Phlorofucofuroeckol-B **(n)** Phloroglucinol **(o)** Triphloroethol-A showing the residues involved in the interaction along with the interaction types. The 3-letter code of each amino acid is shown along with the residue

position and the color of dashed line and the spherical shapes (green: H-bond, orange: Cation/anion charges and salt bridges, pink: π-alkyl, purple: π-π interaction) illustrate the type of interaction.

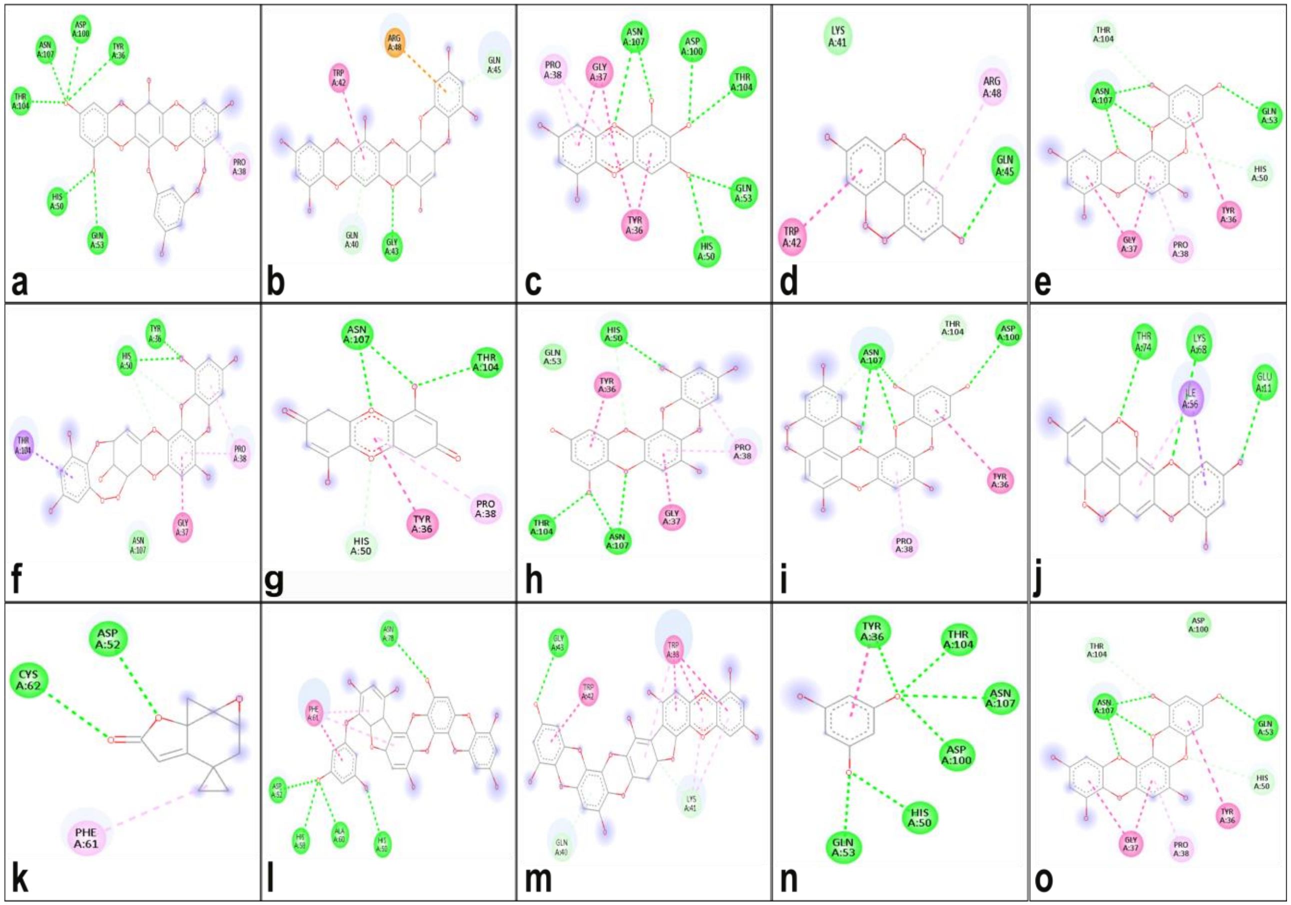

Figure S3. 2D interaction plots of PA-I galactophilic lectin protein in complex with **(a)** 2-Phloroeckol **(b)**7-Phloroeckol **(c)** Bifuhalol **(d)** Difucol **(e)** Dioxinodehydroeckol **(f)** Diphlorethohydroxycarmalol **(g)** Diphlorethol **(h)** Eckol **(i)** Fucodiphloroethol-G **(j)** Fucophlorethol-A **(k)** Isololiolide **(l)** Phlorofucofuroeckol-A **(m)** Phlorofucofuroeckol-B **(n)** Phloroglucinol **(o)** Triphloroethol-A showing the residues involved in the interaction along with the interaction types. The 3-letter code of each amino acid is shown

along with the residue position and the color of dashed lines and the spherical shapes (green: H-bond, orange: Cation/anion charges and salt bridges, pink: π-alkyl, purple: π-π interaction) illustrate the type of interaction.

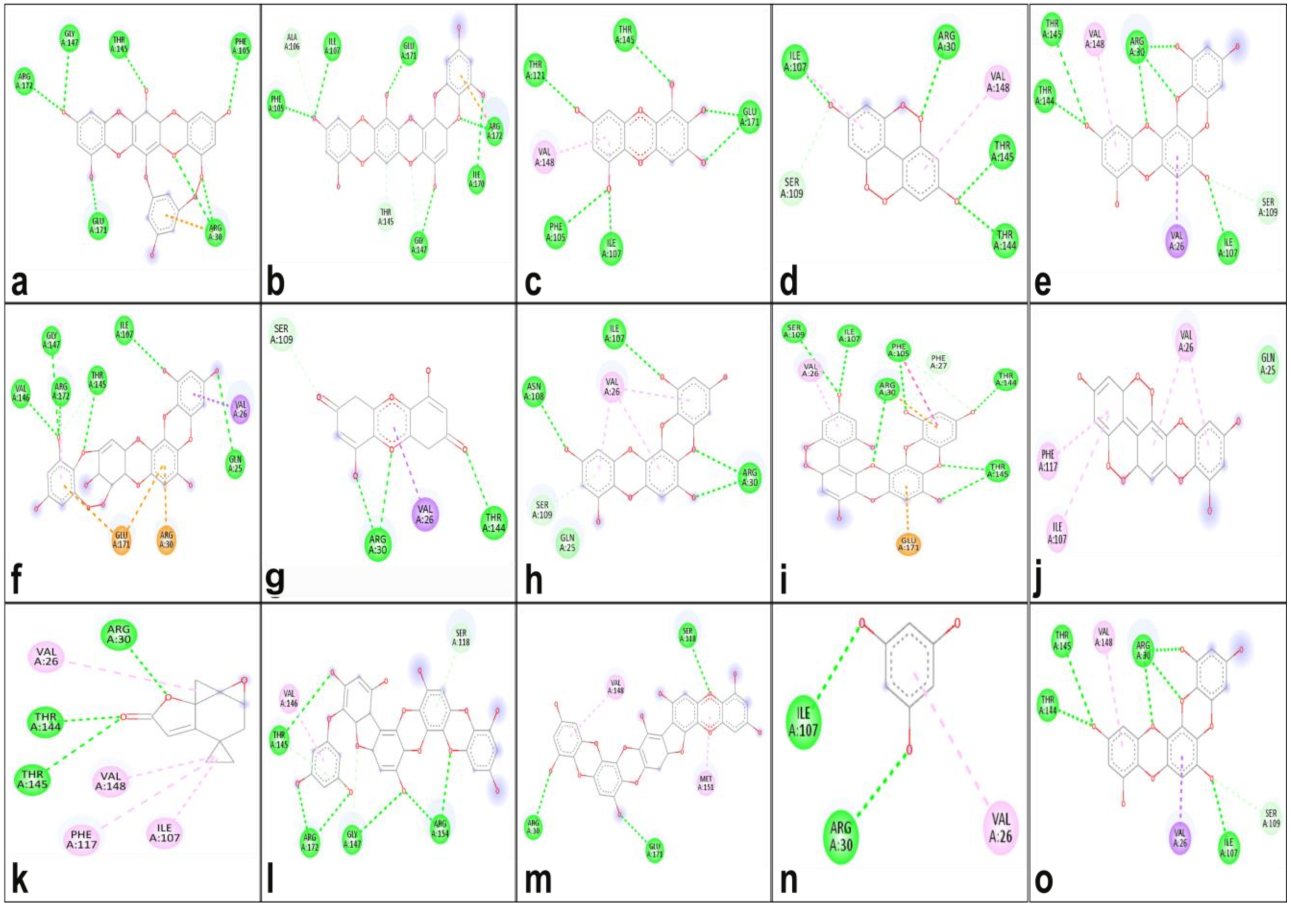

Figure S4. 2D interaction plots of acyl-homoserine-lactone synthase protein in complex with **(a)** 2-Phloroeckol **(b)**7-Phloroeckol **(c)** Bifuhalol **(d)** Difucol **(e)** Dioxinodehydroeckol **(f)** Diphlorethohydroxycarmalol **(g)** Diphlorethol **(h)** Eckol **(i)** Fucodiphloroethol-G **(j)** Fucophlorethol-A **(k)** Isololiolide **(l)** Phlorofucofuroeckol-A **(m)** Phlorofucofuroeckol-B **(n)** Phloroglucinol **(o)** Triphloroethol-A showing the residues involved in the interaction along with the interaction types. The 3-letter code of each amino acid is shown

along with the residue position and the color of dashed line and the spherical shapes (green: H-bond, orange: Cation/anion charges and salt bridges, pink: π-alkyl, purple: π-π interaction) illustrate the type of interaction.

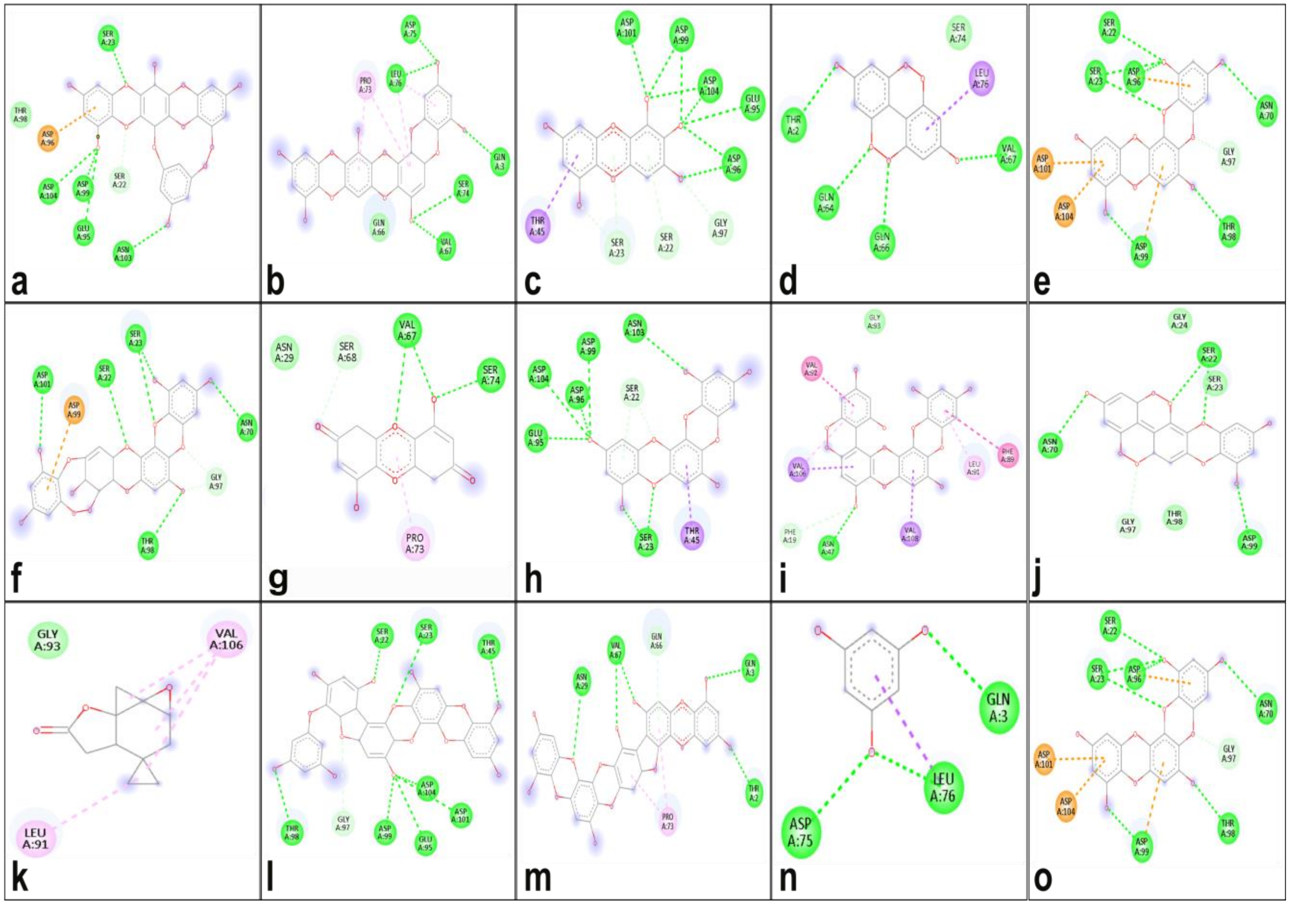

Figure S5. 2D interaction plots of fucose-binding lectin PA-IIL protein in complex with **(a)** 2-Phloroeckol **(b)**7-Phloroeckol **(c)** Bifuhalol **(d)** Difucol **(e)** Dioxinodehydroeckol **(f)** Diphlorethohydroxycarmalol **(g)** Diphlorethol **(h)** Eckol **(i)** Fucodiphloroethol-G **(j)** Fucophlorethol-A **(k)** Isololiolide **(l)** Phlorofucofuroeckol-A **(m)** Phlorofucofuroeckol-B **(n)** Phloroglucinol **(o)** Triphloroethol-A showing the residues involved in the interaction along with the interaction types. The 3-letter code of each amino acid is shown

along with the residue position and the color of dashed line and the spherical shapes (green: H-bond, orange: Cation/anion charges and salt bridges, pink: π-alkyl, purple: π-π interaction) illustrate the type of interaction.

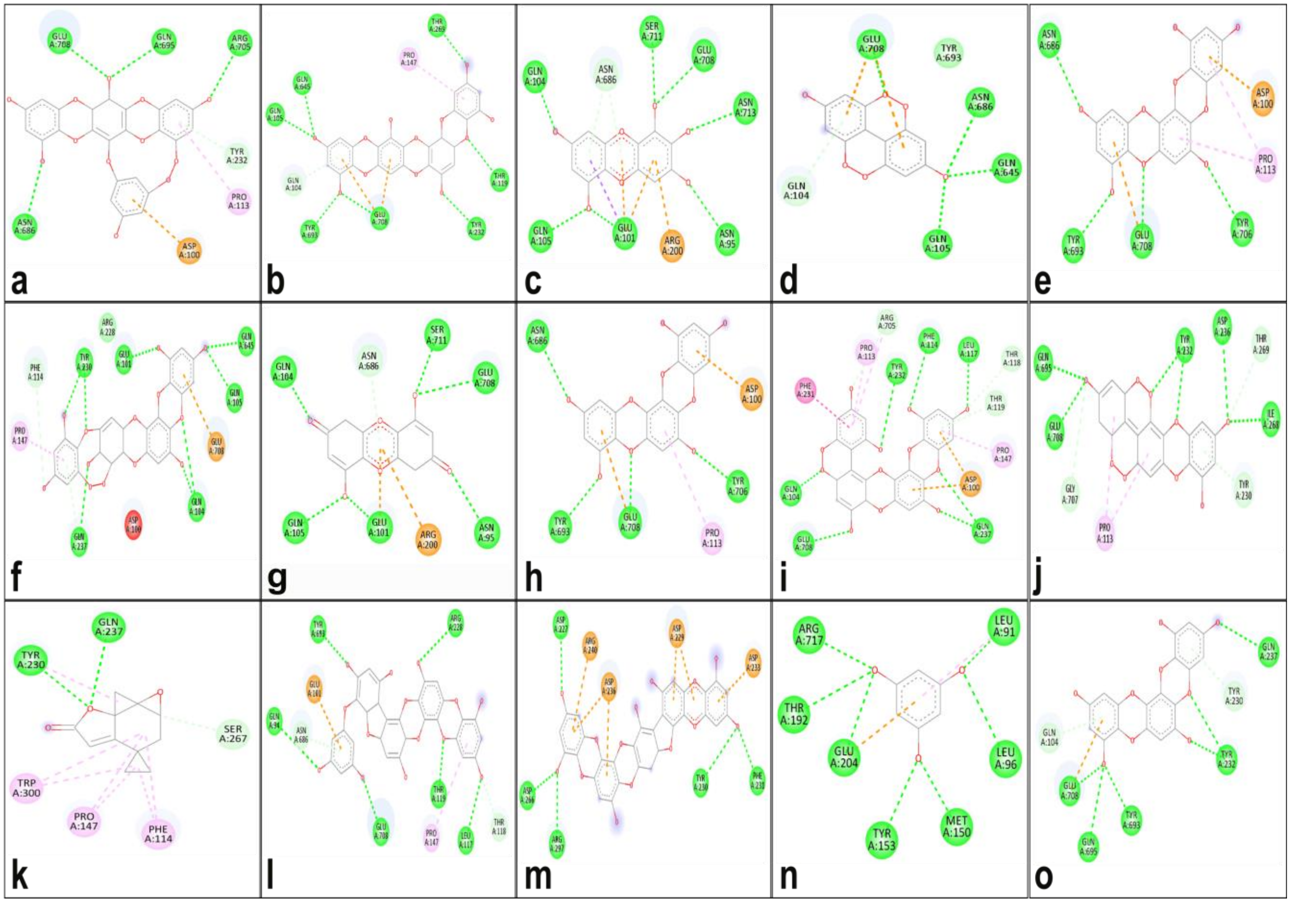

Figure S6. 2D interaction plots of Fe (3+)-pyochelin receptor protein in complex with **(a)** 2-Phloroeckol **(b)**7-Phloroeckol **(c)** Bifuhalol **(d)** Difucol **(e)** Dioxinodehydroeckol **(f)** Diphlorethohydroxycarmalol **(g)** Diphlorethol **(h)** Eckol **(i)** Fucodiphloroethol-G **(j)** Fucophlorethol-A **(k)** Isololiolide **(l)** Phlorofucofuroeckol-A **(m)** Phlorofucofuroeckol-B **(n)** Phloroglucinol **(o)** Triphloroethol-A showing the residues involved in the interaction along with the interaction types. The 3-letter code of each amino acid is shown

along with the residue position and the color of dashed line and the spherical shapes (green: H-bond, orange: Cation/anion charges and salt bridges, pink: π-alkyl, purple: π-π interaction) illustrate the type of interaction.

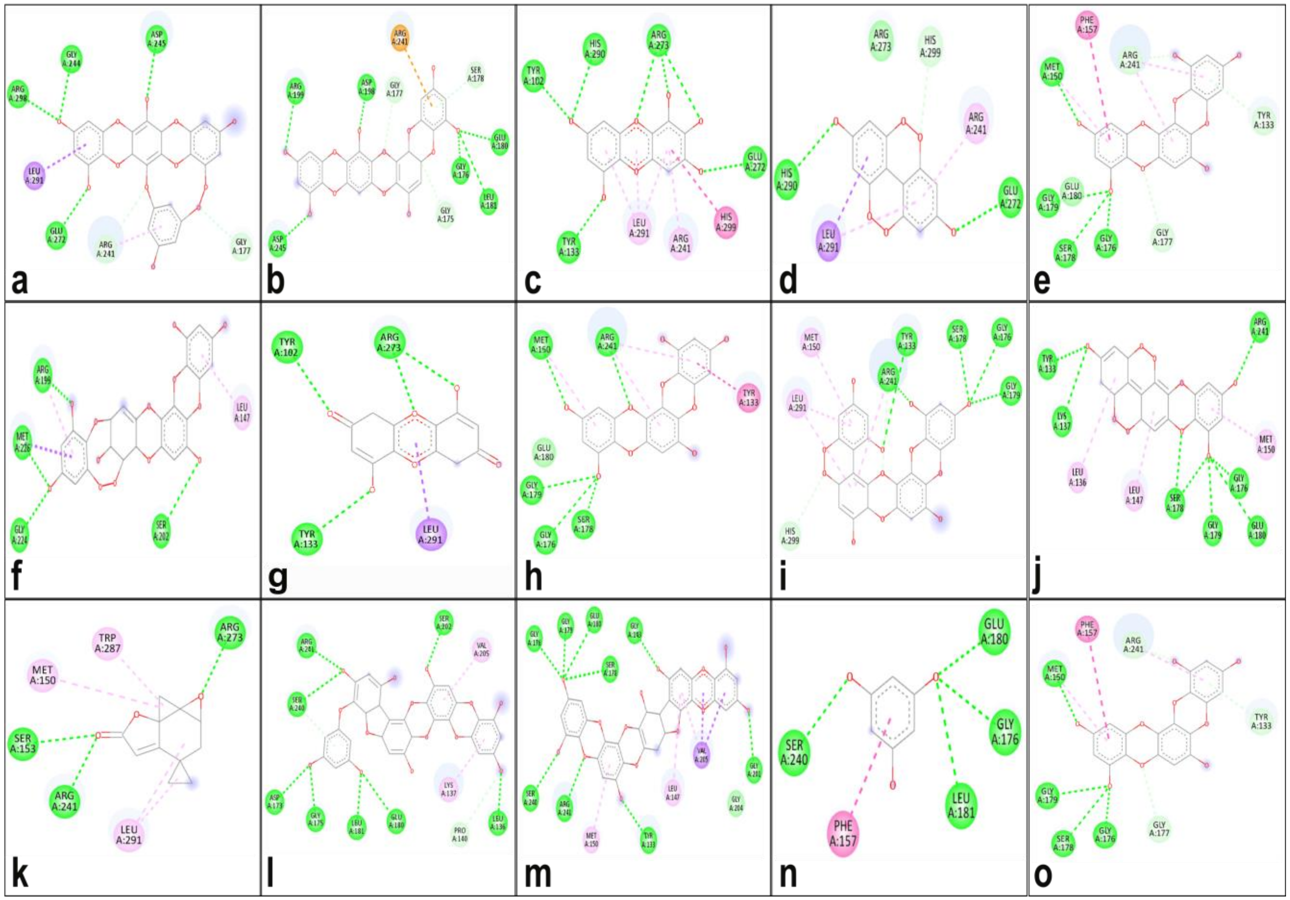

Figure S7. 2D interaction plots of phenazine-1-carboxylate N-methyltransferase protein in complex with **(a)** 2-Phloroeckol **(b)**7-Phloroeckol **(c)** Bifuhalol **(d)** Difucol **(e)** Dioxinodehydroeckol **(f)** Diphlorethohydroxycarmalol **(g)** Diphlorethol **(h)** Eckol **(i)** Fucodiphloroethol-G **(j)** Fucophlorethol-A **(k)** Isololiolide **(l)** Phlorofucofuroeckol-A **(m)** Phlorofucofuroeckol-B **(n)** Phloroglucinol **(o)** Triphloroethol-A showing the residues involved in the interaction along with the interaction types. The 3-letter code of each

amino acid is shown along with the residue position and the color of dashed line and the spherical shapes (green: H-bond, orange: Cation/anion charges and salt bridges, pink: π -alkyl, purple: π-π interaction) illustrate the type of interaction.

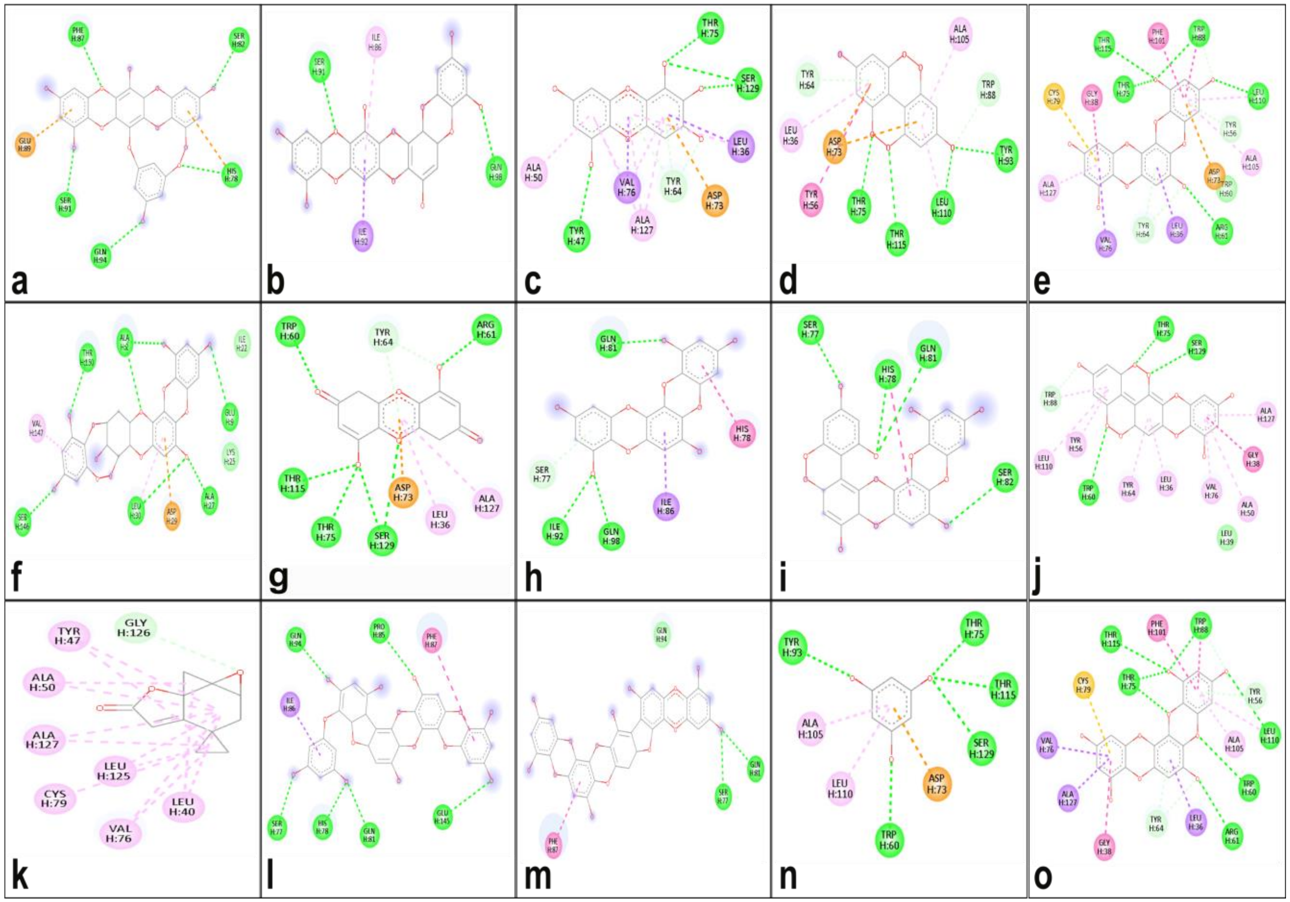

Figure S8. 2D interaction plots of transcriptional activator protein LasR protein in complex with **(a)** 2-Phloroeckol **(b)**7-Phloroeckol **(c)** Bifuhalol **(d)** Difucol **(e)** Dioxinodehydroeckol **(f)** Diphlorethohydroxycarmalol **(g)** Diphlorethol **(h)** Eckol **(i)** Fu-codiphloroethol-G **(j)** Fucophlorethol-A **(k)** Isololiolide **(l)** Phlorofucofuroeckol-A **(m)** Phlorofucofuroeckol-B **(n)** Phloroglucinol **(o)** Triphloroethol-A showing the residues involved in the interaction along with the interaction types. The 3-letter code of each

amino acid is shown along with the residue position and the color of dashed line and the spherical shapes (green: H-bond, orange: Cation/anion charges and salt bridges, pink: π-alkyl, purple: π-π interaction) illustrate the type of interaction.

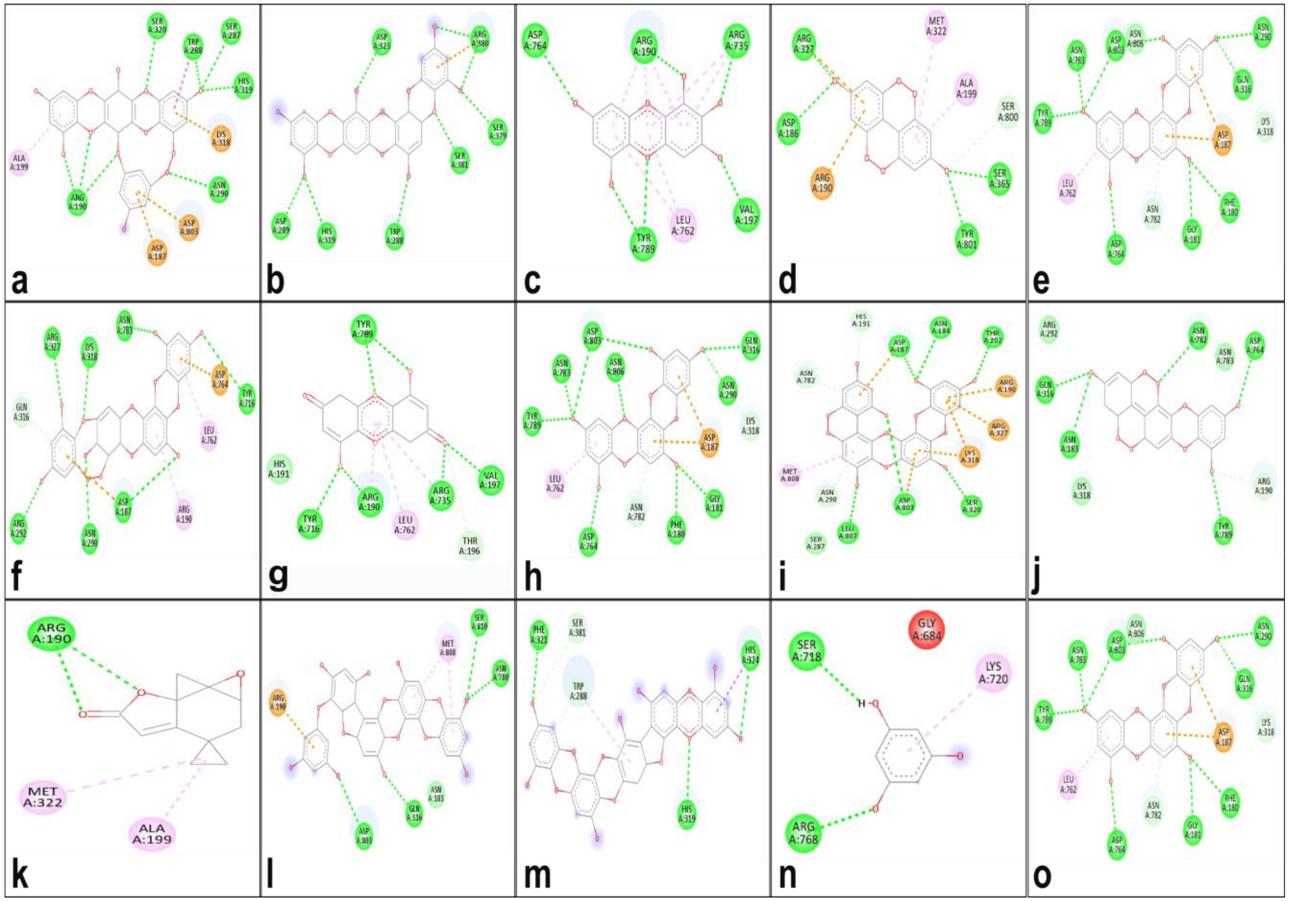

Figure S9. 2D interaction plots of ferripyoverdine receptor protein in complex with **(a)** 2-Phloroeckol **(b)**7-Phloroeckol **(c)** Bifuhalol **(d)** Difucol **(e)** Dioxinodehydroeckol **(f)** Diphlorethohydroxycarmalol **(g)** Diphlorethol **(h)** Eckol **(i)** Fucodiphloroethol-G **(j)** Fucophlorethol-A **(k)** Isololiolide **(l)** Phlorofucofuroeckol-A **(m)** Phlorofucofuroeckol-B **(n)** Phloroglucinol **(o)** Triphloroethol-A showing the residues involved in the interaction along with the interaction types. The 3-letter code of each amino acid is shown

along with the residue position and the color of dashed line and the spherical shapes (green: H-bond, orange: Cation/anion charges and salt bridges, pink: π-alkyl, purple: π-π interaction) illustrate the type of interaction.

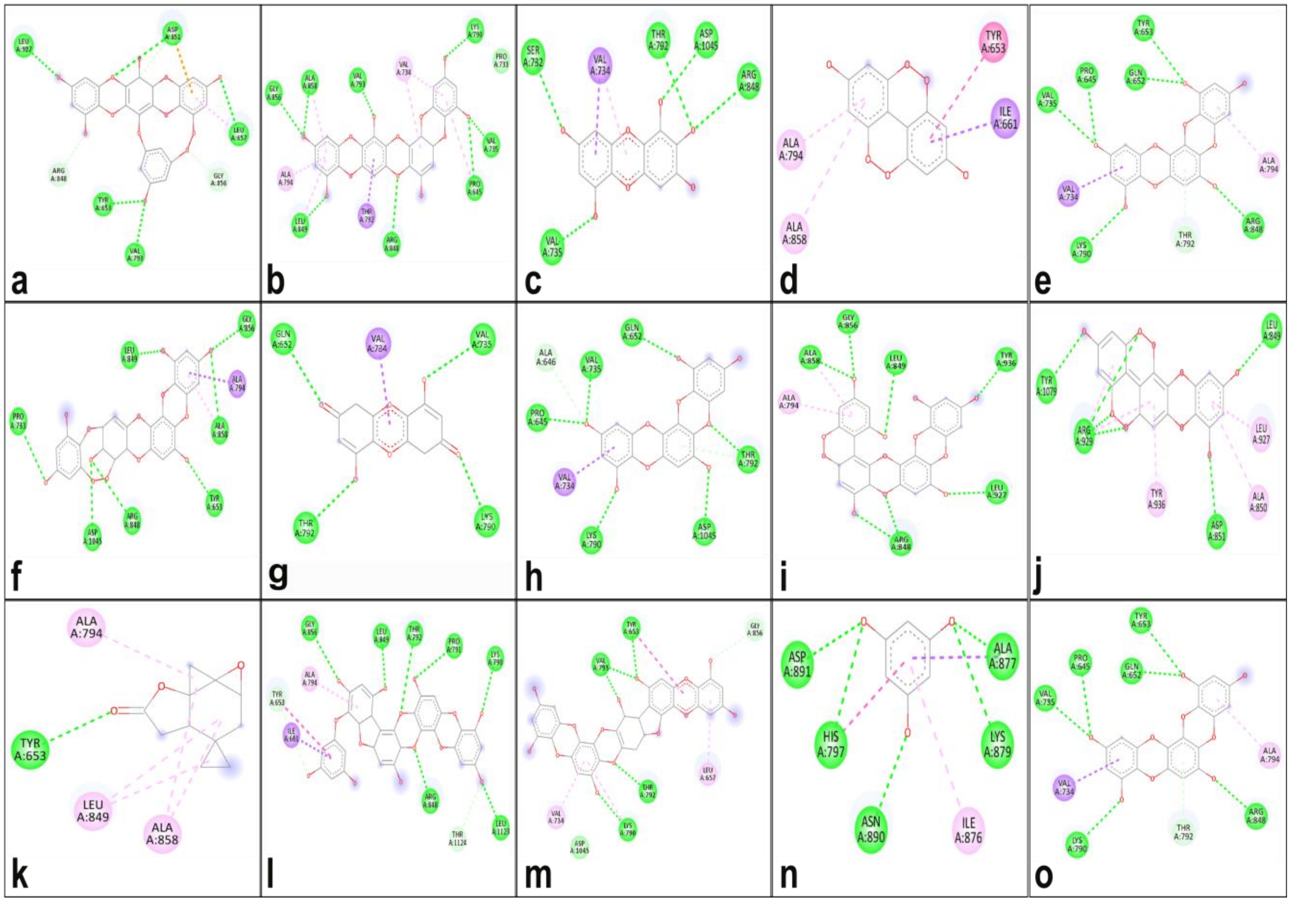

Figure S10. 2D interaction plots of type IV pilus biogenesis factor PilY1protein in complex with **(a)** 2-Phloroeckol **(b)**7-Phloroeckol **(c)** Bifuhalol **(d)** Difucol **(e)** Dioxinodehydroeckol **(f)** Diphlorethohydroxycarmalol **(g)** Diphlorethol **(h)** Eckol **(i)** Fucodiphloroethol-G **(j)** Fucophlorethol-A **(k)** Isololiolide **(l)** Phlorofucofuroeckol-A **(m)** Phlorofucofuroeckol-B **(n)** Phloroglucinol **(o)** Triphloroethol-A showing the residues involved in the interaction along with the interaction types. The 3-letter code of each

amino acid is shown along with the residue position and the color of dashed line and the spherical shapes (green: H-bond, orange: Cation/anion charges and salt bridges, pink: π-alkyl, purple: π-π interaction) illustrate the type of interaction.

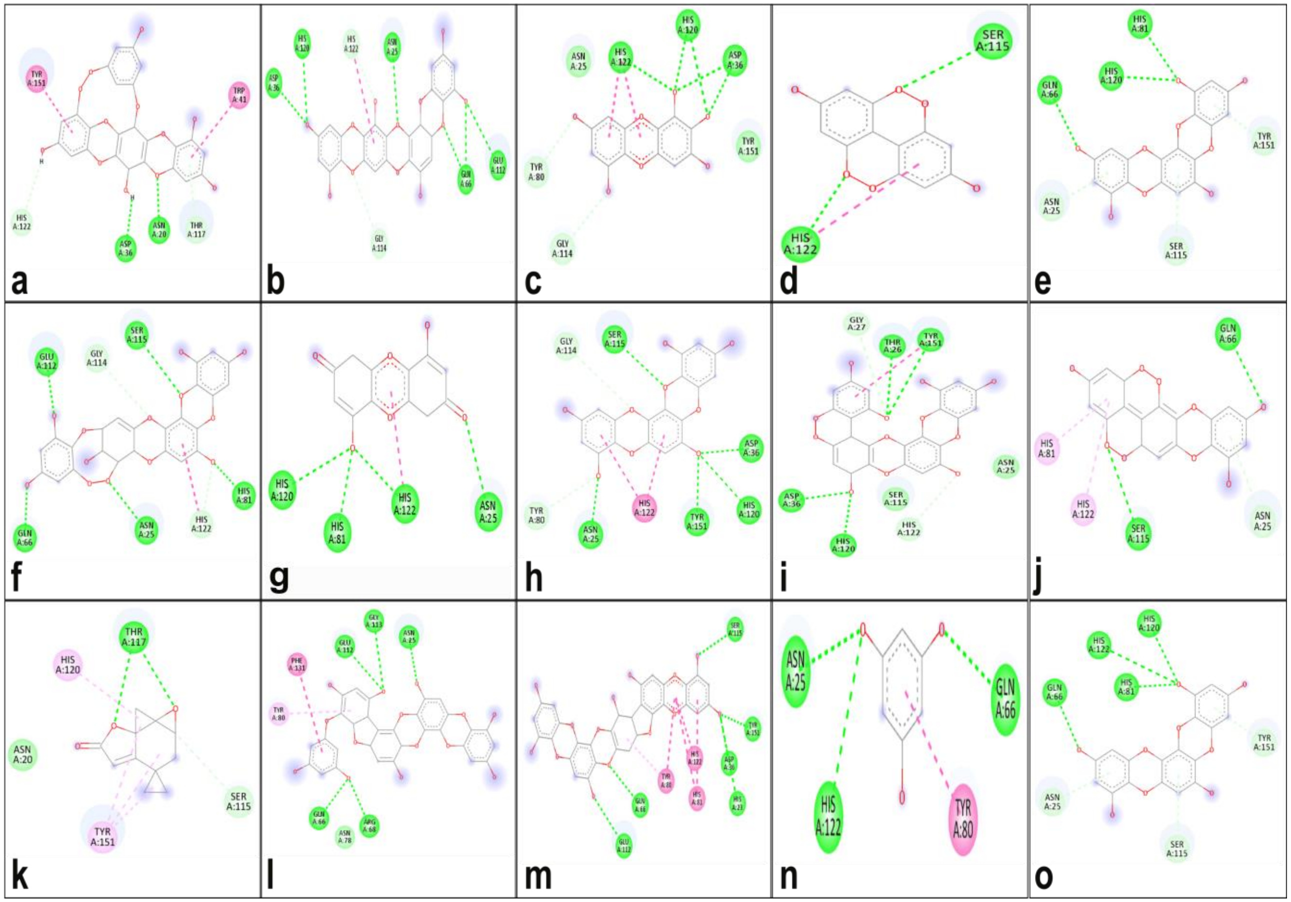

Figure S11. 2D interaction plots of protease LasA protein in complex with **(a)** 2-Phloroeckol **(b)**7-Phloroeckol **(c)** Bifuhalol **(d)** Difucol **(e)** Dioxinodehydroeckol **(f)** Diphlorethohydroxycarmalol **(g)** Diphlorethol **(h)** Eckol **(i)** Fucodiphloroethol-G **(j)** Fuco-phlorethol-A **(k)** Isololiolide **(l)** Phlorofucofuroeckol-A **(m)** Phlorofucofuroeckol-B **(n)** Phloroglucinol **(o)** Triphloroethol-A showing the residues involved in the interaction along with the interaction types. The 3-letter code of each amino acid is shown along

with the residue position and the color of dashed line and the spherical shapes (green: H-bond, orange: Cation/anion charges and salt bridges, pink: π-alkyl, purple: π-π interaction) illustrate the type of interaction.

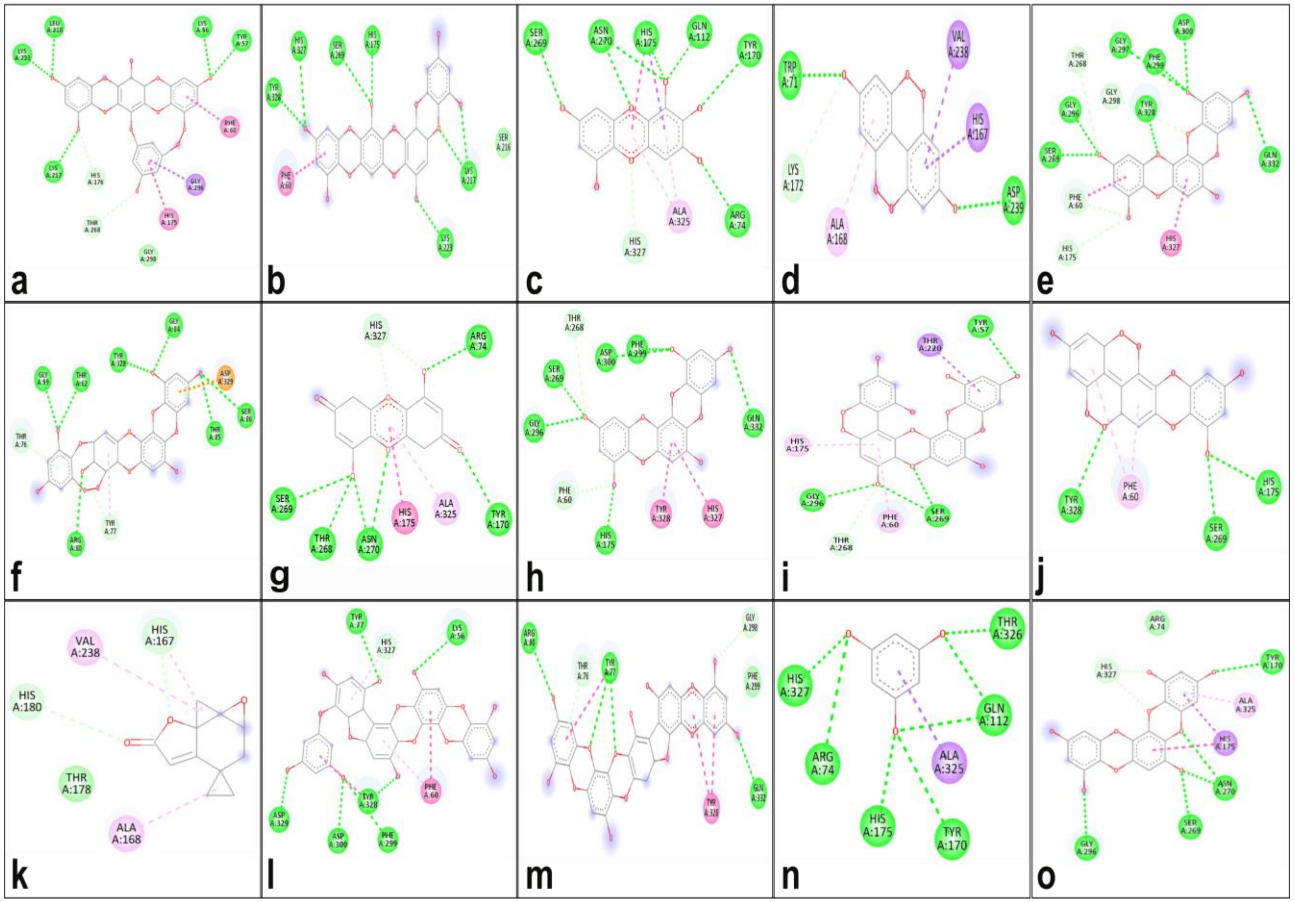

Figure S12. 2D interaction plots of alginate biosynthesis protein AlgX in complex with **(a)** 2-Phloroeckol **(b)**7-Phloroeckol **(c)** Bifuhalol **(d)** Difucol **(e)** Dioxinodehydroeckol **(f)** Diphlorethohydroxycarmalol **(g)** Diphlorethol **(h)** Eckol **(i)** Fucodiphloroethol-G **(j)** Fucophlorethol-A **(k)** Isololiolide **(l)** Phlorofucofuroeckol-A **(m)** Phlorofucofuroeckol-B **(n)** Phloroglucinol **(o)** Triphloroethol-A showing the residues involved in the interaction along with the interaction types. The 3-letter code of each amino acid is shown

along with the residue position and the color of dashed line and the spherical shapes (green: H-bond, orange: Cation/anion charges and salt bridges, pink: π-alkyl, purple: π-π interaction) illustrate the type of interaction.

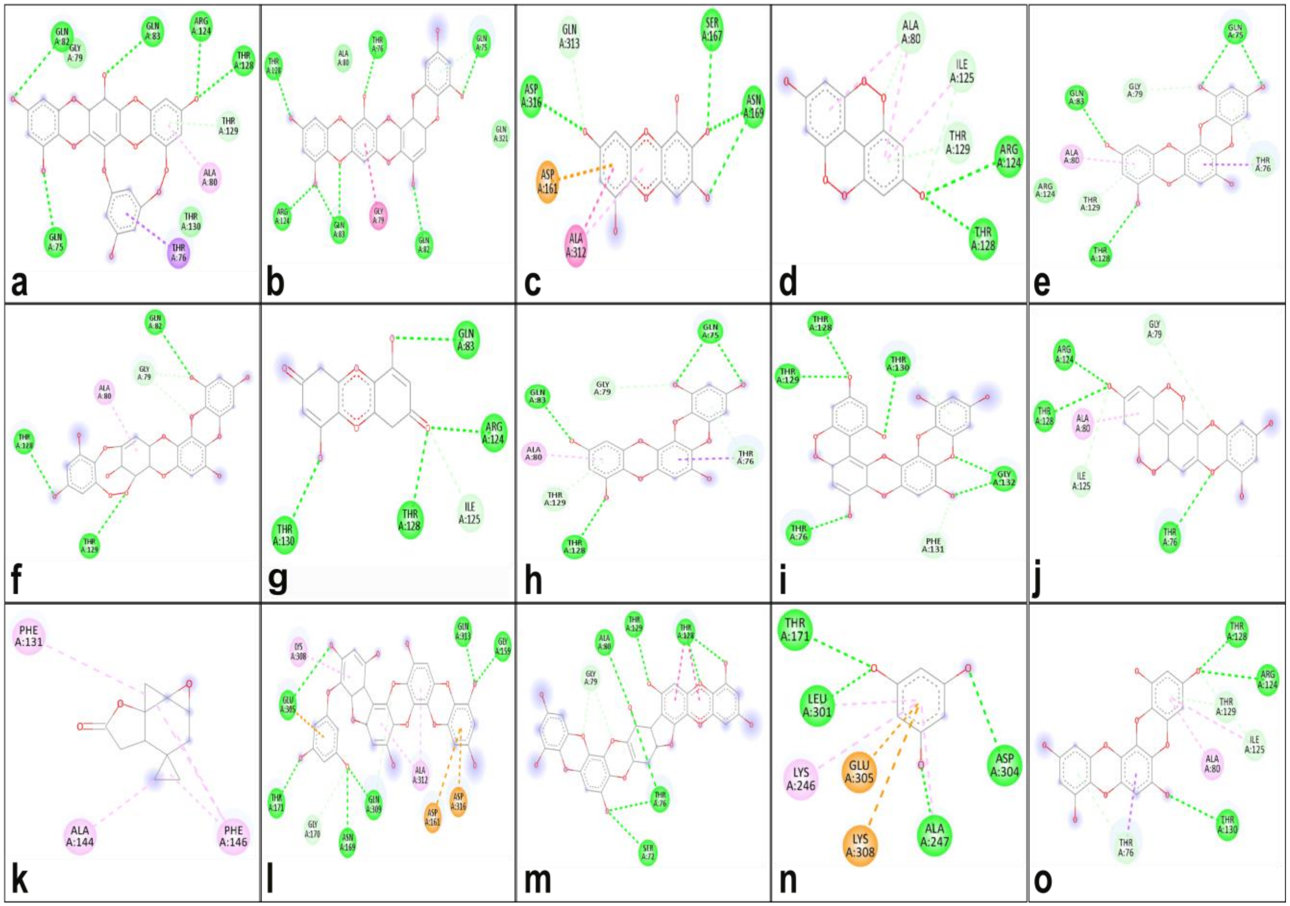

Figure S13. 2D interaction plots of flagellin protein in complex with **(a)** 2-Phloroeckol **(b)**7-Phloroeckol **(c)** Bifuhalol **(d)** Difucol **(e)** Dioxinodehydroeckol **(f)** Diphlorethohydroxycarmalol **(g)** Diphlorethol **(h)** Eckol **(i)** Fucodiphloroethol-G **(j)** Fucophlorethol-A **(k)** Isololiolide **(l)** Phlorofucofuroeckol-A **(m)** Phlorofucofuroeckol-B **(n)** Phloroglucinol **(o)** Triphloroethol-A showing the residues involved in the interaction along with the interaction types. The 3-letter code of each amino acid is shown along with the resi-

due position and the color of dashed line and the spherical shapes (green: H-bond, orange: Cation/anion charges and salt bridges, pink: π-alkyl, purple: π-π interaction) illustrate the type of interaction.

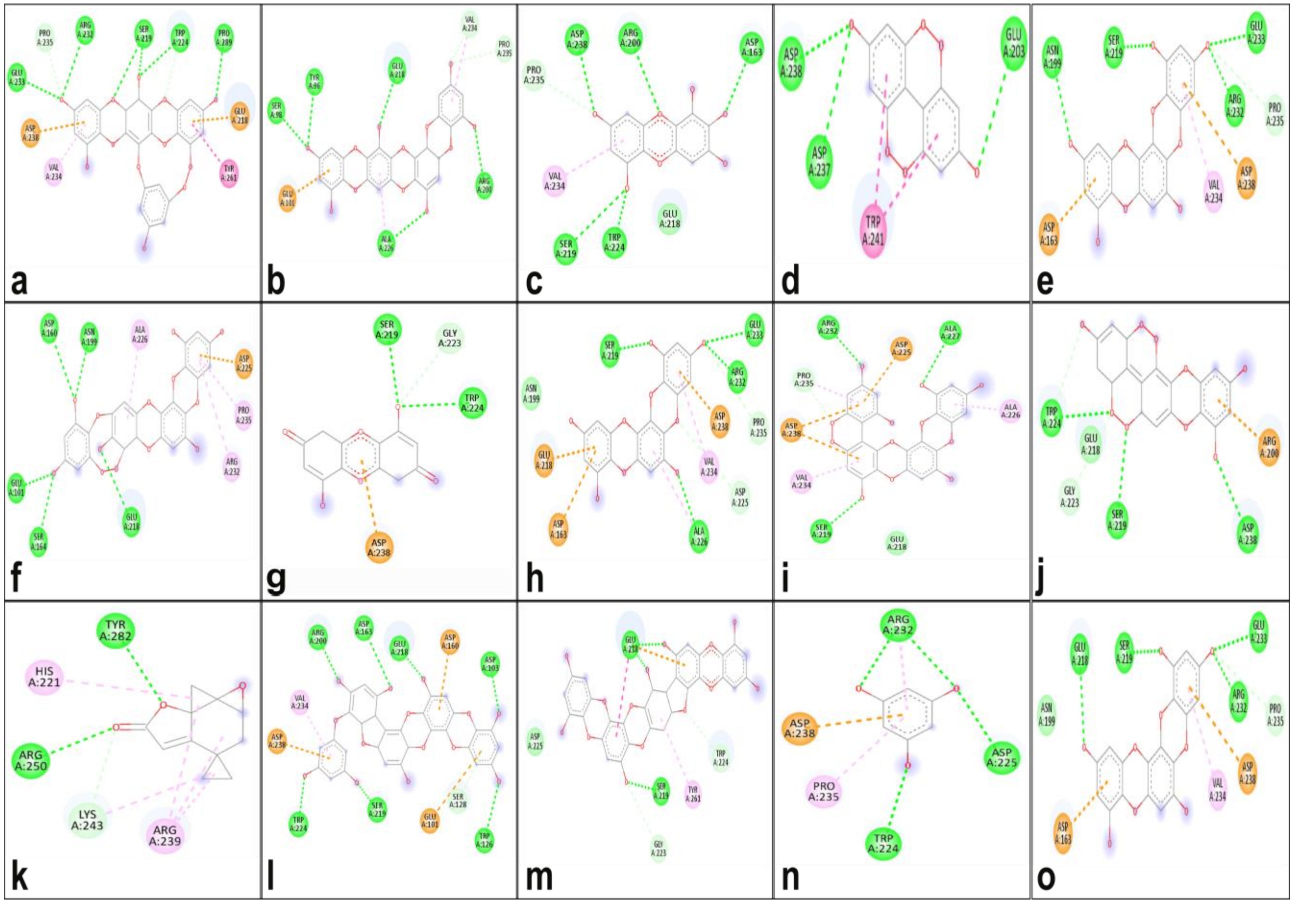

Figure S14. 2D interaction plots of PelA protein in complex with **(a)** 2-Phloroeckol **(b)**7-Phloroeckol **(c)** Bifuhalol **(d)** Difucol **(e)** Dioxinodehydroeckol **(f)** Diphlorethohydroxycarmalol **(g)** Diphlorethol **(h)** Eckol **(i)** Fucodiphloroethol-G **(j)** Fucophlorethol-A **(k)** Isololiolide **(l)** Phlorofucofuroeckol-A **(m)** Phlorofucofuroeckol-B **(n)** Phloroglucinol **(o)** Triphloroethol-A showing the residues involved in the interaction along with the interaction types. The 3-letter code of each amino acid is shown along with the residue

position and the color of dashed line and the spherical shapes (green: H-bond, orange: Cation/anion charges and salt bridges, pink: π-alkyl, purple: π-π interaction) illustrate the type of interaction.

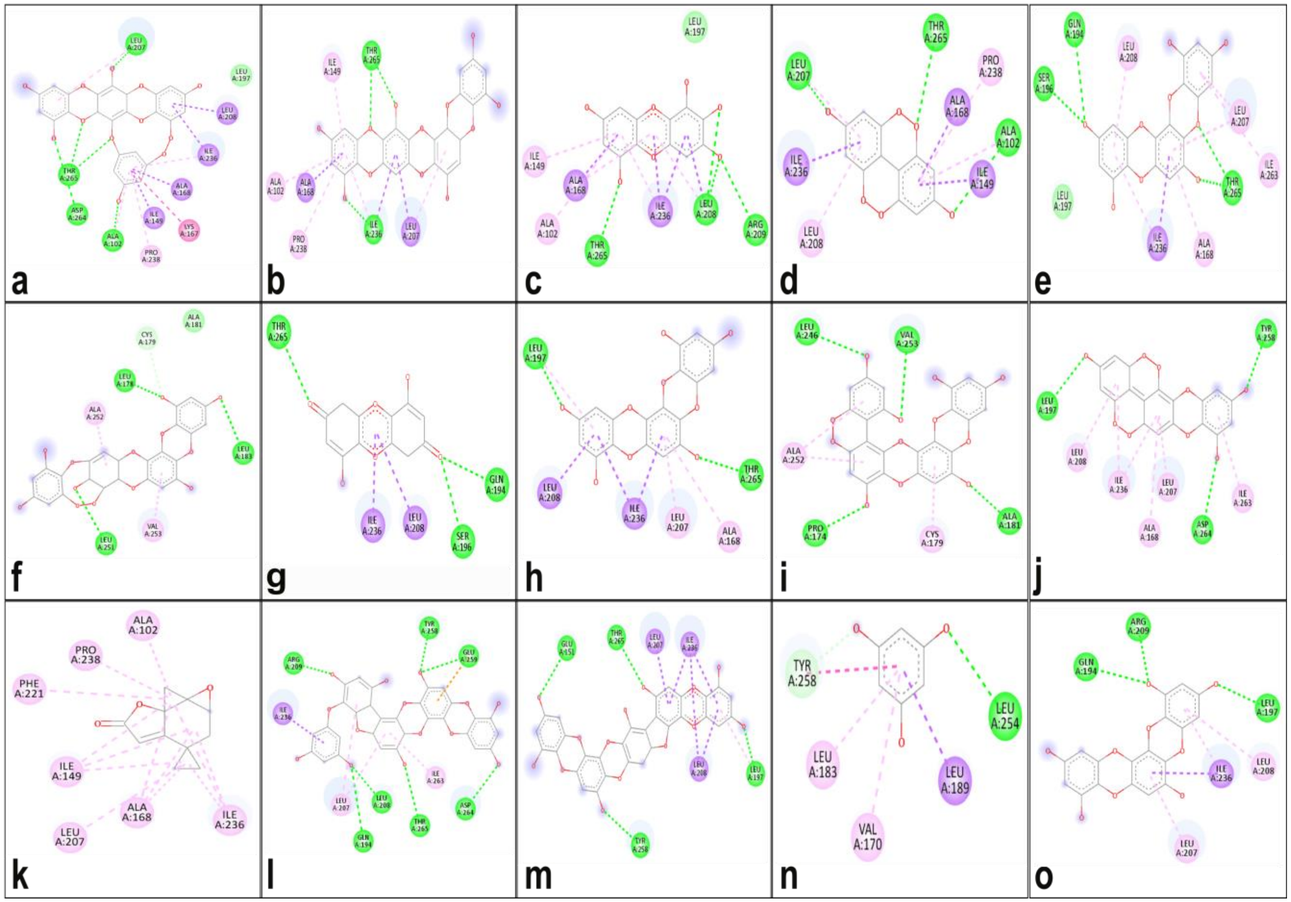

Figure S15. 2D interaction plots of multiple virulence factor regulator MvfR protein in complex with **(a)** 2-Phloroeckol **(b)**7-Phloroeckol **(c)** Bifuhalol **(d)** Difucol **(e)** Dioxinodehydroeckol **(f)** Diphlorethohydroxycarmalol **(g)** Diphlorethol **(h)** Eckol **(i)** Fucodiphloroethol-G **(j)** Fucophlorethol-A **(k)** Isololiolide **(l)** Phlorofucofuroeckol-A **(m)** Phlorofucofuroeckol-B **(n)** Phloroglucinol **(o)** Triphloroethol-A showing the residues involved in the interaction along with the interaction types. The 3-letter code of each

amino acid is shown along with the residue position and the color of dashed line and the spherical shapes (green: H-bond, orange: Cation/anion charges and salt bridges, pink: π-alkyl, purple: π-π interaction) illustrate the type of interaction.

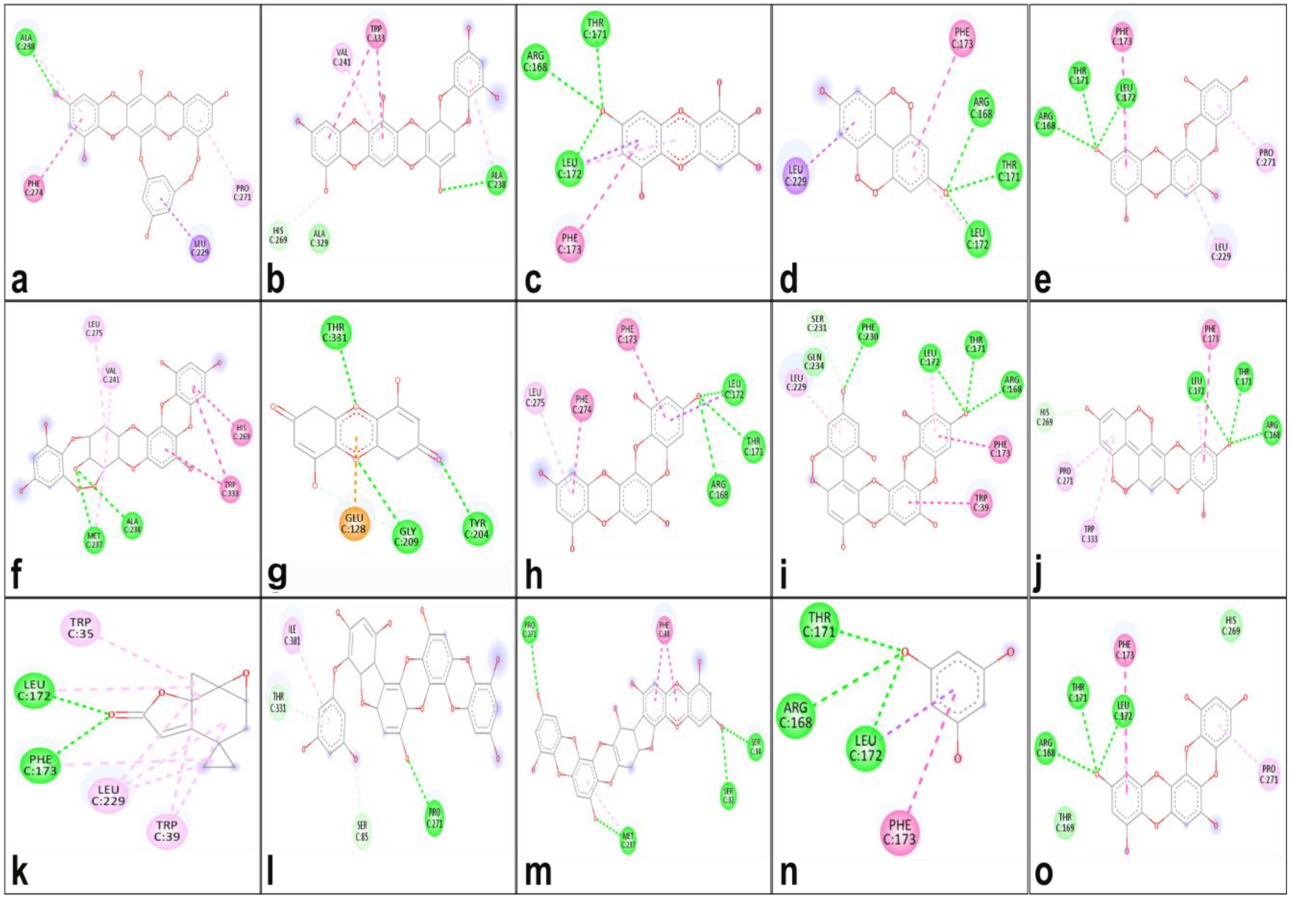

Figure S16. 2D interaction plots of 2-heptyl-4(1H)-quinolone synthase subunit PqsB protein in complex with **(a)** 2-Phloroeckol **(b)**7-Phloroeckol **(c)** Bifuhalol **(d)** Difucol **(e)** Dioxinodehydroeckol **(f)** Diphlorethohydroxycarmalol **(g)** Diphlorethol **(h)** Eckol **(i)** Fucodiphloroethol-G **(j)** Fucophlorethol-A **(k)** Isololiolide **(l)** Phlorofucofuroeckol-A **(m)** Phlorofucofuroeckol-B **(n)** Phloroglu-cinol **(o)** Triphloroethol-A showing the residues involved in the interaction along with the interaction types. The 3-letter code of

each amino acid is shown along with the residue position and the color of dashed line and the spherical shapes (green: H-bond, orange: Cation/anion charges and salt bridges, pink: π-alkyl, purple: π-π interaction) illustrate the type of interaction.

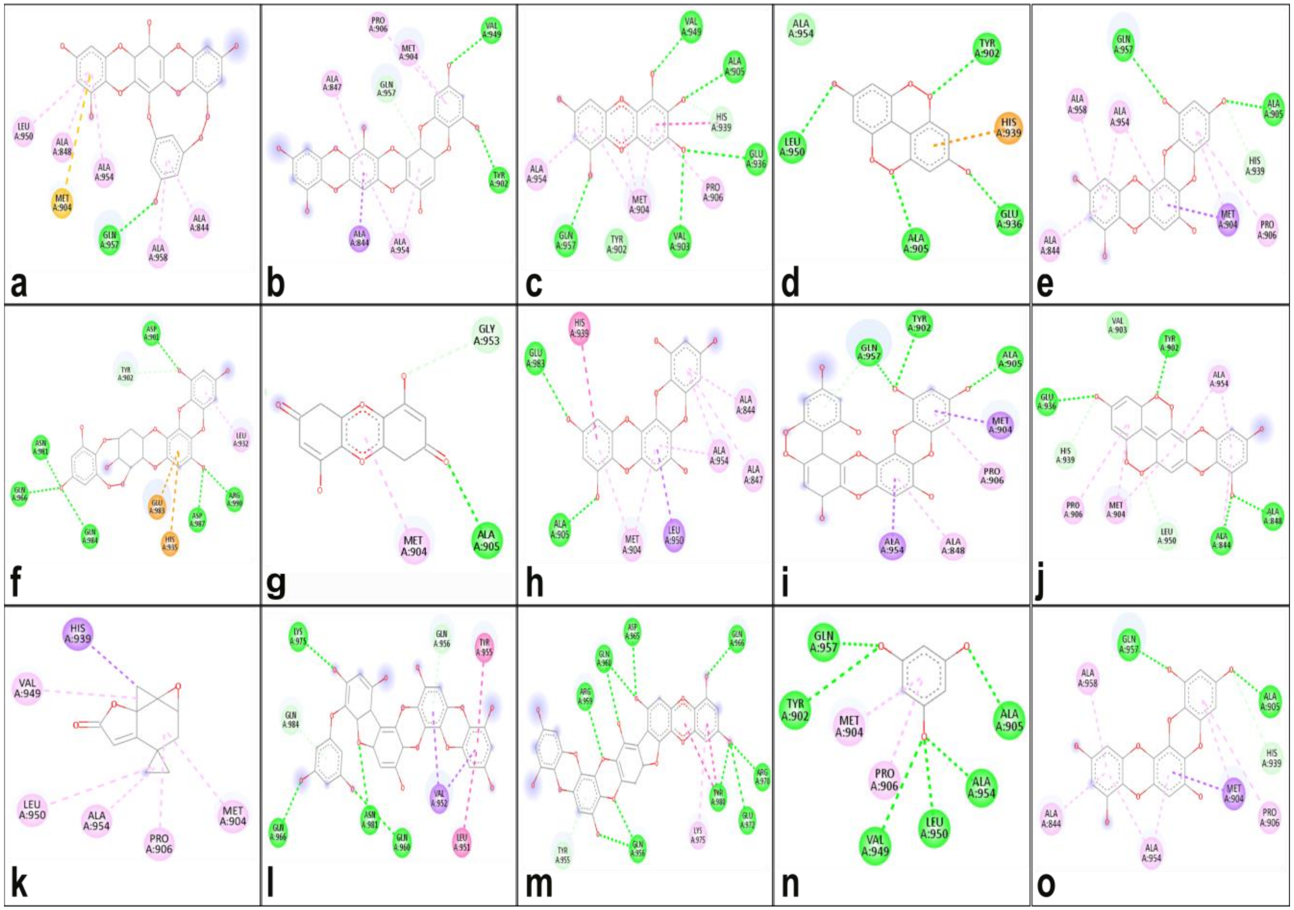

Figure S17. 2D interaction plots of type VI secretion system spike protein VgrG2b protein in complex with (a) 2-Phloroeckol **(b)**7-Phloroeckol **(c)** Bifuhalol **(d)** Difucol **(e)** Dioxinodehydroeckol **(f)** Diphlorethohydroxycarmalol **(g)** Diphlorethol **(h)** Eckol **(i)** Fu-codiphloroethol-G **(j)** Fucophlorethol-A **(k)** Isololiolide **(l)** Phlorofucofuroeckol-A **(m)** Phlorofucofuroeckol-B **(n)** Phloroglucinol **(o)** Triphloroethol-A showing the residues involved in the interaction along with the interaction types. The 3-letter code of each

amino acid is shown along with the residue position and the color of dashed line and the spherical shapes (green: H-bond, orange: Cation/anion charges and salt bridges, pink: π-alkyl, purple: π-π interaction) illustrate the type of interaction.

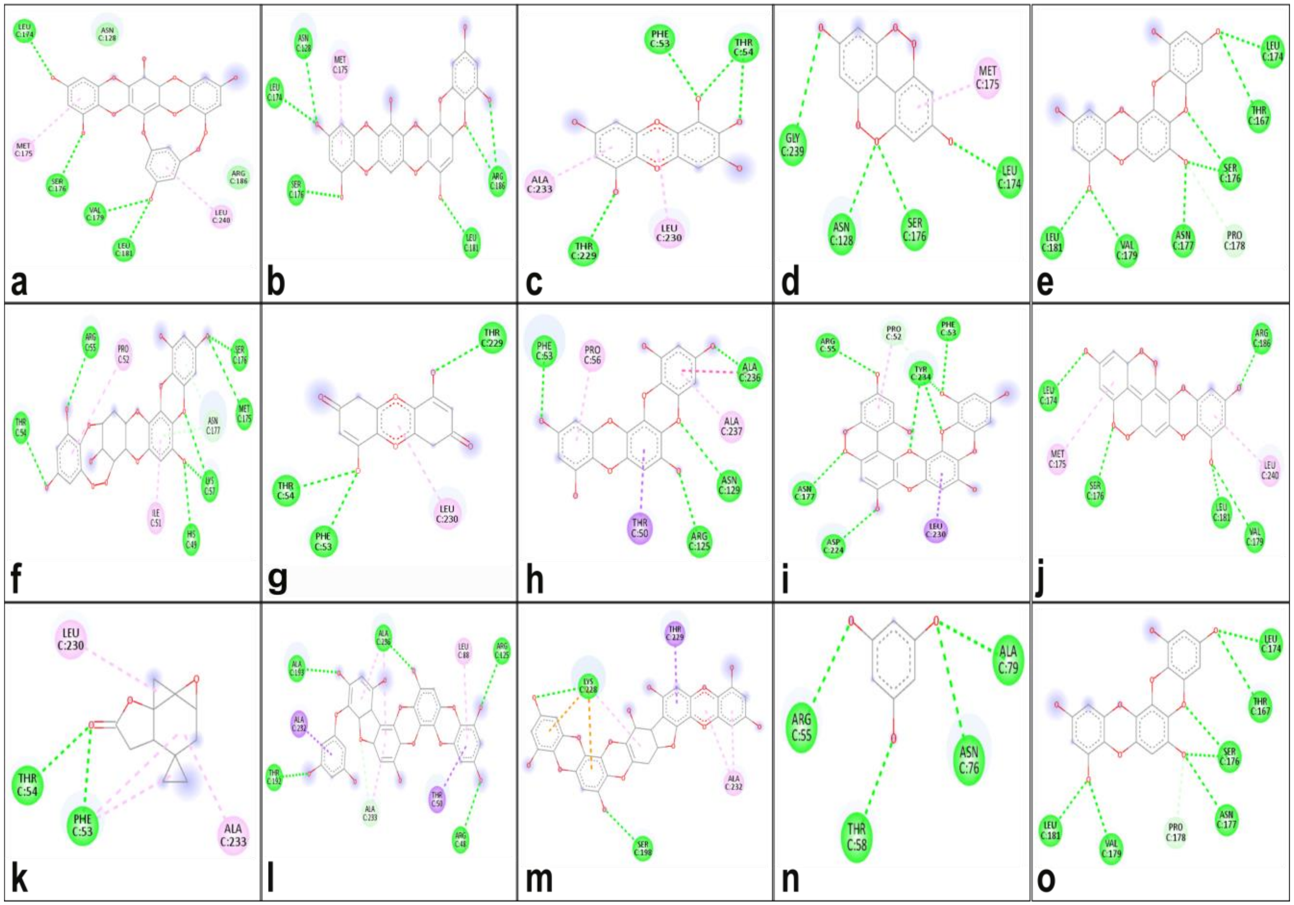

Figure S18. 2D interaction plots of HTH-type quorum-sensing regulator RhlR protein in complex with (a) 2-Phloroeckol **(b)**7-Phloroeckol **(c)** Bifuhalol **(d)** Difucol **(e)** Dioxinodehydroeckol **(f)** Diphlorethohydroxycarmalol **(g)** Diphlorethol **(h)** Eckol **(i)** Fucodiphloroethol-G **(j)** Fucophlorethol-A **(k)** Isololiolide **(l)** Phlorofucofuroeckol-A **(m)** Phlorofucofuroeckol-B **(n)** Phloroglucinol **(o)** Triphloroethol-A showing the residues involved in the interaction along with the interaction types. The 3-letter code of each

amino acid is shown along with the residue position, and the color of the dashed line and the spherical shapes (green: H-bond, orange: Cation/anion charges and salt bridges, pink: π-alkyl, purple: π-π interaction) illustrate the type of interaction.

References

Berni B, Soscia C, Djermoun S, Ize B, Bleves S (2019) A Type VI Secretion System Trans-Kingdom Effector Is Required for the Delivery of a Novel Antibacterial Toxin in *Pseudomonas aeruginosa*. Front Microbiol 10:1218 doi:10.3389/fmicb.2019.01218

Bottomley MJ, Muraglia E, Bazzo R, Carfì A (2007) Molecular Insights into Quorum Sensing in the Human Pathogen *Pseudomonas aeruginosa* from the Structure of the Virulence Regulator LasR Bound to Its Autoinducer*. Journal of Biological Chemistry 282(18):13592-13600 doi:https://doi.org/10.1074/jbc.M700556200

Braun P, de Groot A, Bitter W, Tommassen J (1998) Secretion of elastinolytic enzymes and their propeptides by *Pseudomonas aeruginosa*. J Bacteriol 180(13):3467-9 doi:10.1128/jb.180.13.3467-3469.1998

Brint JM, Ohman DE (1995) Synthesis of multiple exoproducts in *Pseudomonas aeruginosa* is under the control of RhlR-RhlI, another set of regulators in strain PAO1 with homology to the autoinducer-responsive LuxR-LuxI family. J Bacteriol 177(24):7155-63 doi:10.1128/jb.177.24.7155-7163.1995

Cahan R, Axelrad I, Safrin M, Ohman DE, Kessler E (2001) A secreted aminopeptidase of *Pseudomonas aeruginosa*. Identification, primary structure, and relationship to other aminopeptidases. J Biol Chem 276(47):43645-52 doi:10.1074/jbc.M106950200

Cao H, Krishnan G, Goumnerov B, Tsongalis J, Tompkins R, Rahme LG (2001) A quorum sensing-associated virulence gene of *Pseudomonas aeruginosa* encodes a LysR-like transcription regulator with a unique self-regulatory mechanism. Proc Natl Acad Sci U S A 98(25):14613-8 doi:10.1073/pnas.251465298

Cioci G, Mitchell EP, Gautier C, Wimmerová M, Sudakevitz D, Pérez S, Gilboa-Garber N, Imberty A (2003) Structural basis of calcium and galactose recognition by the lectin PA-IL of *Pseudomonas aeruginosa*. FEBS Lett 555(2):297-301 doi:10.1016/s0014-5793(03)01249-3

Cobessi D, Celia H, Pattus F (2005) Crystal structure at high resolution of ferric-pyochelin and its membrane receptor FptA from *Pseudomonas aeruginosa*. J Mol Biol 352(4):893-904 doi:10.1016/j.jmb.2005.08.004

Drees SL, Li C, Prasetya F, Saleem M, Dreveny I, Williams P, Hennecke U, Emsley J, Fetzner S (2016) PqsBC, a Condensing Enzyme in the Biosynthesis of the *Pseudomonas aeruginosa* Quinolone Signal: CRYSTAL STRUCTURE, INHIBITION, AND REACTION MECHANISM. J Biol Chem 291(13):6610-24 doi:10.1074/jbc.M115.708453

Dulcey CE, Dekimpe V, Fauvelle DA, Milot S, Groleau MC, Doucet N, Rahme LG, Lépine F, Déziel E (2013) The end of an old hypothesis: the *Pseudomonas* signaling molecules 4-hydroxy-2-alkylquinolines derive from fatty acids, not 3-ketofatty acids. Chem Biol 20(12):1481-91 doi:10.1016/j.chembiol.2013.09.021

Gould TA, Schweizer HP, Churchill ME (2004) Structure of the *Pseudomonas aeruginosa* acyl-homoserinelactone synthase LasI. Mol Microbiol 53(4):1135-46 doi:10.1111/j.1365-2958.2004.04211.x

Greenwald J, Nader M, Celia H, Gruffaz C, Geoffroy V, Meyer JM, Schalk IJ, Pattus F (2009) FpvA bound to non-cognate pyoverdines: molecular basis of siderophore recognition by an iron transporter. Mol Microbiol 72(5):1246-59 doi:10.1111/j.1365-2958.2009.06721.x

Heiniger RW, Winther-Larsen HC, Pickles RJ, Koomey M, Wolfgang MC (2010) Infection of human mucosal tissue by *Pseudomonas aeruginosa* requires sequential and mutually dependent virulence factors and a novel pilus-associated adhesin. Cell Microbiol 12(8):1158-73 doi:10.1111/j.1462-5822.2010.01461.x

Johnson MD, Garrett CK, Bond JE, Coggan KA, Wolfgang MC, Redinbo MR (2011) *Pseudomonas aeruginosa* PilY1 binds integrin in an RGD- and calcium-dependent manner. PLoS One 6(12):e29629 doi:10.1371/journal.pone.0029629

Latifi A, Winson MK, Foglino M, Bycroft BW, Stewart GS, Lazdunski A, Williams P (1995) Multiple homologues of LuxR and LuxI control expression of virulence determinants and secondary metabolites through quorum sensing in *Pseudomonas aeruginosa* PAO1. Mol Microbiol 17(2):333-43 doi:10.1111/j.1365-2958.1995.mmi_17020333.x

Maura D, Hazan R, Kitao T, Ballok AE, Rahme LG (2016) Evidence for Direct Control of Virulence and Defense Gene Circuits by the *Pseudomonas aeruginosa* Quorum Sensing Regulator, MvfR. Sci Rep 6:34083 doi:10.1038/srep34083

McIver K, Kessler E, Ohman DE (1991) Substitution of active-site His-223 in *Pseudomonas aeruginosa* elastase and expression of the mutated lasB alleles in Escherichia coli show evidence for autoproteolytic processing of proelastase. J Bacteriol 173(24):7781-9 doi:10.1128/jb.173.24.7781-7789.1991

Medina G, Juárez K, Valderrama B, Soberón-Chávez G (2003) Mechanism of *Pseudomonas aeruginosa* RhlR transcriptional regulation of the rhlAB promoter. J Bacteriol 185(20):5976-83 doi:10.1128/jb.185.20.5976-5983.2003

Mitchell EP, Sabin C, Snajdrová L, Pokorná M, Perret S, Gautier C, Hofr C, Gilboa-Garber N, Koca J, Wimmerová M, Imberty A (2005) High affinity fucose binding of *Pseudomonas aeruginosa* lectin PA-IIL: 1.0 A resolution crystal structure of the complex combined with thermodynamics and computational chemistry approaches. Proteins 58(3):735-46 doi:10.1002/prot.20330

Morihara K, Tsuzuki H, Oka T (1973) On the specificity of *Pseudomonas aeruginosa* alkaline proteinase with synthetic peptides. Biochim Biophys Acta 309(2):414-29 doi:10.1016/0005-2744(73)90040-5

Ochsner UA, Koch AK, Fiechter A, Reiser J (1994) Isolation and characterization of a regulatory gene affecting rhamnolipid biosurfactant synthesis in *Pseudomonas aeruginosa*. J Bacteriol 176(7):2044-54 doi:10.1128/jb.176.7.2044-2054.1994

Ochsner UA, Reiser J (1995) Autoinducer-mediated regulation of rhamnolipid biosurfactant synthesis in *Pseudomonas aeruginosa*. Proc Natl Acad Sci U S A 92(14):6424-8 doi:10.1073/pnas.92.14.6424

Olson JC, Ohman DE (1992) Efficient production and processing of elastase and LasA by *Pseudomonas aeruginosa* require zinc and calcium ions. J Bacteriol 174(12):4140-7 doi:10.1128/jb.174.12.4140-4147.1992

Orans J, Johnson MD, Coggan KA, Sperlazza JR, Heiniger RW, Wolfgang MC, Redinbo MR (2010) Crystal structure analysis reveals Pseudomonas PilY1 as an essential calcium-dependent regulator of bacterial surface motility. Proc Natl Acad Sci U S A 107(3):1065-70 doi:10.1073/pnas.0911616107

Parsons JF, Greenhagen BT, Shi K, Calabrese K, Robinson H, Ladner JE (2007) Structural and functional analysis of the pyocyanin biosynthetic protein PhzM from *Pseudomonas aeruginosa*. Biochemistry 46(7):1821-8 doi:10.1021/bi6024403

Riley LM, Weadge JT, Baker P, Robinson H, Codée JD, Tipton PA, Ohman DE, Howell PL (2013) Structural and functional characterization of *Pseudomonas aeruginosa* AlgX: role of AlgX in alginate acetylation. J Biol Chem 288(31):22299-314 doi:10.1074/jbc.M113.484931

Robles-Price A, Wong TY, Sletta H, Valla S, Schiller NL (2004) AlgX is a periplasmic protein required for alginate biosynthesis in *Pseudomonas aeruginosa*. J Bacteriol 186(21):7369-77 doi:10.1128/jb.186.21.7369-7377.2004

Sana TG, Baumann C, Merdes A, Soscia C, Rattei T, Hachani A, Jones C, Bennett KL, Filloux A, Superti-Furga G, Voulhoux R, Bleves S (2015) Internalization of *Pseudomonas aeruginosa* Strain PAO1 into Epithelial Cells Is Promoted by Interaction of a T6SS Effector with the Microtubule Network. mBio 6(3):e00712 doi:10.1128/mBio.00712-15

Snarr BD, Baker P, Bamford NC, Sato Y, Liu H, Lehoux M, Gravelat FN, Ostapska H, Baistrocchi SR, Cerone RP, Filler EE, Parsek MR, Filler SG, Howell PL, Sheppard DC (2017) Microbial glycoside hydrolases as antibiofilm agents with cross-kingdom activity. Proceedings of the National Academy of Sciences 114(27):7124-7129 doi:doi:10.1073/pnas.1702798114

Song WS, Yoon SI (2014) Crystal structure of FliC flagellin from *Pseudomonas aeruginosa* and its implication in TLR5 binding and formation of the flagellar filament. Biochem Biophys Res Commun 444(2):109-15 doi:10.1016/j.bbrc.2014.01.008

Spencer J, Murphy LM, Conners R, Sessions RB, Gamblin SJ (2010) Crystal structure of the LasA virulence factor from *Pseudomonas aeruginosa*: substrate specificity and mechanism of M23 metallopeptidases. J Mol Biol 396(4):908-23 doi:10.1016/j.jmb.2009.12.021

Vessillier S, Delolme F, Bernillon J, Saulnier J, Wallach J (2001) Hydrolysis of glycine-containing elastin pentapeptides by LasA, a metalloelastase from *Pseudomonas aeruginosa*. Eur J Biochem 268(4):1049-57 doi:10.1046/j.1432-1327.2001.01967.x

Wagner VE, Gillis RJ, Iglewski BH (2004) Transcriptome analysis of quorum-sensing regulation and virulence factor expression in *Pseudomonas aeruginosa*. Vaccine 22 Suppl 1:S15-20 doi:10.1016/j.vaccine.2004.08.011

Wood TE, Howard SA, Förster A, Nolan LM, Manoli E, Bullen NP, Yau HCL, Hachani A, Hayward RD, Whitney JC, Vollmer W, Freemont PS, Filloux A (2019) The *Pseudomonas aeruginosa* T6SS Delivers a Periplasmic Toxin that Disrupts Bacterial Cell Morphology. Cell Rep 29(1):187-201.e7 doi:10.1016/j.celrep.2019.08.094

Xiao G, Déziel E, He J, Lépine F, Lesic B, Castonguay MH, Milot S, Tampakaki AP, Stachel SE, Rahme LG (2006) MvfR, a key *Pseudomonas aeruginosa* pathogenicity LTTR-class regulatory protein, has dual ligands. Mol Microbiol 62(6):1689-99 doi:10.1111/j.1365-2958.2006.05462.x
